# Supplementary material for: Impact of targeted diabetic retinopathy training for graders in Vietnam and the implications for future diabetic retinopathy screening programmes: a diagnostic test accuracy study
Source: BMJ Open. 2022 Sep 9;12(9):e059205. doi: 10.1136/bmjopen-2021-059205 (PMC9472142; doi:10.1136/bmjopen-2021-059205)
Supplement: Supplementary data [file bmjopen-2021-059205supp001.pdf]

SUPPLEMENTARY MATERIAL

Figure S1: Training timeframe for graders in Vietnam

September 2017 (5 days)

- Five Vietnamese doctors and medical administrators visited Northern Ireland to receive screener/graders training in administration and failsafe methods.
- These doctors delivered training to new graders in Vietnam following their visit to Northern Ireland.

January 2018 (1 week) – Delivered in person in Vietnam by two UK graders (senior ophthalmic nurse and optometrist, certified in DR grading)

- Observation in retina clinics.
- Hands on training with tabletop CR-2 Canon Fundus Cameras.
- Topics covered: ocular anatomy, retinal disease, DR signs, DR grading (based on the UK DESP grading classification system) and appropriate referral pathways and management (PowerPoint presentation and interactive sessions).
- All graders received a module workbook.
- Certifcation provided by Orbis.

March 2018 - Grading began in Vietnam

- Graders began grading as part of pilot DESP.
- Ongoing training was delivered by the Orbis team and the lead ophthalmologist for DR screening in Vietnam over the course of the following months.

June 2018 (1-2 days) – Delivered by Orbis partners

- UK graders developed a PowerPoint presentation based on DR case examples and this was delivered by Orbis.

March 2019 (2 days) - Delivered by UK grader in Vietnam

- More training on DR case examples.

November 2019 (3 days) - Delivered in person in Vietnam by two UK graders (senior ophthalmic nurse and optometrist, certified in DR grading)

- Refresher DR training, incorporating think-aloud techniques into practical teaching sessions.
- Pre and post training assessments.
- Encouraged use of international test and training (iTAT) for quality assurance purposes. Practical sessions on iTAT.

Table S1: Diabetic retinopathy workshop for graders in Vietnam

**AGENDA**<br><br>DAY 1-3 **Monday 29th January – Wednesday 31st January**		
Visit diabetic retinopathy screening sites to observe and provide hands on training and support.		UK graders and Orbis team
DAY 4**- Thursday 1st February**		
**MORNING**		
**9:00 - 9:30**	Check in	UK graders and Orbis team
**9:30 – 10.30**	Introduction on Diabetic Retinopathy	
**10.30-10.45**	Tea Break	
**10.45-11.30**	Basic Screening Component	
	Lunch	
**AFTERNOON**		
**13.30-14:00**	NHS Grading System	UK graders and Orbis team
**14:00-14:45**	Image Quality	
**14:45-15:00**	Tea Break	
**15:00-16:00**	Image Grading	
**16:0-16:30**	Hospital pathway	
DAY 5**- Friday 2nd February**		
**MORNING**		
**9:00-9:30**	Other ocular findings	UK graders and Orbis team
**9:30-11:30**	Practice on grading	
**11:30-12:00**	Wrap up	

Table S2: Refresher training programme for DR graders in Vietnam (delivered by UK graders and Orbis) in November 2019

Time	Topics	Method	Who	Preparation
	**DAY 1**			
**8:45-9:15**	Introduction/ Pre course quiz		Orbis VN/ UK graders	UK graders
**9:15-9:45**	**Diabetic Retinopathy (DR) - New Challenges of Blindness Prevention** *Objective: Understand the problem of DR and current efforts to manage vision loss. Aim to motivate graders to be involved in DESP*	Presentation	Orbis VN	Orbis
**9:45-10:15**	**Retina Anatomy** *Objective : Understand the pathobiology of diabetic complications and pathogenesis of retinal damage*	Presentation	Ho Chi Minh Eye Hospital	Orbis
**10:30-11:15**	**Diabetic Retinopathy (DR) Pathophysiology** *Objective : Understand Diabetic Retinopathy*	Presentation	UK graders	UK graders
**11:15-12:00**	**Grading system and DR Grading pathway (UK system)/ How to systematically grade a retinal image** *Objective : Understanding the grading system and referral pathway (UK standard)*	Presentation	UK graders	UK graders
**13:30-14:15**	**Image quality** *Objective : Understand the requirements/criteria of image quality for accurate grading*	Presentation	UK graders	UK graders
**14:15-14:45**	**Spectra Software** *Objective : How to use the current Spectra software for uploading, grading, and managing DR cases*	Demonstration	Senior graders of Tien Giang and Ho Chi Minh Eye Hospital	

**15:00-17:00**	**<u>Practical Training</u>**<br>**Parallel session:**<br>**1- Taking retina images of the patients following the procedure ,and provide counseling to the patients**<br><br>*Objective: Practice experience of taking fundus images*	Practical training	Current graders of Tien Giang and Ho Chi Minh Eye Hospital, and UK graders	2 fundus cameras:<br>4 Groups: make sure every participant is able to practice at least once
**15:00-17:00**	**Parallel session :**<br>**1- Grading DR in the Spectra**<br><br>*Objective: Practical experience of how to do DR grading*	Practical training	Current graders of Tien Giang and Ho Chi Minh Eye Hospital, and UK graders	3-4 accounts of Spectra<br>4 Groups : make sure every participant is able to practice at least once
		**DAY 2**		
**08:45-09:00**	*Recap of day 1/ introduction to day 2*		Orbis	
**9:00-9:30**	**Analysis of retinal fundus images for grading of diabetic retinopathy severity**<br>Objective : How to read the image and protocol for retinal image analysis		Ophthalmologist Ho Chi Minh Eye Hospital	
**9:30-10:15**	**DR Screening Procedure: Best Practice**<br>*Objective: Discuss how to build the "best screening procedures" into DESPs.*	Presentation	UK graders	UK graders
**10:30-11:15**	**Other Ocular Findings**<br><br>*Objective: Awareness of other ocular pathology during DR screening*	Presentation	UK graders	UK graders
**11:15-12:15**	**Image grading case studies competition**	Practical	UK graders	We need to organise people into groups of 3 with one experienced grader in each group

**13:30-14:30**	**Counselling and delivering messages to patients during the DR screening** *Objective: Important to provide counselling for the patients and deliver messages effectively.*	Presentation/ practical training	Orbis Vietnam	
**14:30-16:30**	<u>**Practical Training**</u> **Parallel session:** **1- Taking retina images of the patients following the procedure ,and provide counseling to the patients** *Objective: Experience on how to take good fundus photographs*	Practical training	Current graders of Tien Giang and Ho Chi Minh Eye Hospital, and UK graders	2 fundus camera 4 Group: make sure every participants are able to practice at least one time
	**Parallel session :** **1- Grading DR in the Spectra** *Objective: Practical experience on DR grading*	Practical training	Current graders of Tien Giang and Ho Chi Minh Eye Hospital, and UK graders	-4 accounts of Spectra 4 Groups: make sure every participant is able to practice at least one time.
**DAY 3 Part 1 (Final practical training)**				
**08:45-9:00**	Recap of day 2/ introduction to day 3			
**09:00-09:30**	**Quality Assurance in Diabetic Screening** *Objective : Understand why quality assurance is important and the correct steps required to ensure good quality assurance procedures are in place*	Presentation	UK graders	UK graders
**09:30-10:30**	**Practice** : **Grading in iTAT** *Objective: Know the Online training for DR grading and the importance of lifelong learning for DR grading*	Practice	Current graders of Tien Giang and Ho Chi Minh Eye Hospital, and support from UK graders	ITAT accounts for practicing

**10:45-12:30**	**<u>Assessment</u>**<br><br>**Parallel session:**<br>**1- Taking retina images of the patients following the procedure ,and provide counseling to the patients**<br>**2- Grading DR in the Spectra**	Practical Training	Current graders of Tien Giang and Ho Chi Minh Eye Hospital, and UK graders	2 fundus cameras<br>4 Group and 4 accounts of Spectra<br>4 Groups
**DAY 3<br>Part 2 (Future planning)**				
**13:30-14:15**	**Post course Quiz and results**		UK graders	UK graders
**14:15-15:00**	**Teaching Methodology for adults**<br>*Objective : How to train new graders effectively*	Think aloud work shop	Orbis VN Supported by UK graders	UK graders
**15:15-16:00**	**Supportive Supervision Methodology: Developing quality improvement**<br>*Objective: How to plan, implement the supervision trips to correct / improve other graders' performances. Provide checklist tools*	Think aloud work shop	Orbis VN Supported by UK graders	UK graders
**16:00-16:45**	**Feedbacks and Plan for next steps**	Discussion		
**16:45-17:00**	**Certificates for Vietnamese graders in attendance**			

Table S3: UK DR Grading Classification Scale

NSC	International Term	Symptoms	Features	Action
**R0**	No DR	None	No signs of diabetic retinopathy	Annual rescreen
**RI**	Mild none-proliferative (mild pre-proliferative)	None	Haemorrhages & microaneurysms, only	Annual rescreen
**R2**	Moderate none-proliferative, moderate pre-proliferative	None	Extensive Microaneurysms, intraretinal haemorrhages, hard exudates, venous abnormalities, large blot haemorrhages, cotton wool spots (small infarcts), venous beading, venous loop, venous reduplication.	Refer routinely to HES
**R3s**	Stable proliferative diabetic retinopathy		No haemorrhages or exudates or new vessels, laser scars	Annual rescreen
**R3a**	Active proliferative diabetic retinopathy	Floaters, central loss of vision	New vessel formation either at the disc (NVD) or elsewhere (NVE). Extensive fibrovascular proliferation, retinal detachment, pre-retinal or vitreous haemorrhage.	Urgent referral to HES
**M 0**			No maculopathy	Annual rescreen
**M 1**	Diabetic maculopathy	Blurred central vision	The macula is defined as a circle centred on the fovea, with a radius of the distance to the disc margin.<br>If the leakage involves or is near the fovea the condition is termed clinically significant macular oedema (CSME).<br><br>Exudative maculopathy presents with leakage, retinal thickening, microaneurysms, hard exudates at the macula. Ischaemic form can have a featureless macular with NVE and poor vision.<br>Milder forms:<br>• exudate < or = 1DD of centre of fovea<br>• circinate or group of exudates within macula<br>• any microaneurysm or haemorrhage < or = 1DD of centre of fovea only is associated with a best VA of < or = 6/12 retinal thickening < or =	Refer to HES

			1DD of centre of fovea (if stereos available)	
**P**	Photocoagulation	Reduced night vision, glare	Small retinal scars throughout the peripheral retina.	
**U**	Ungradable		Ungradable is usually due to cataract, small pupils, other lesions usually referred for assessment	Refer for slit lamp examination
**Abbreviations:** DR = diabetic retinopathy, NPDR = none-proliferative retinopathy, NVE = new vessels elsewhere, IRMAs = intraretinal microvascular abnormalities (part of severe pre-proliferative retinopathy, vessels will not leak with angiogram, otherwise they would be 'new vessels' making the condition 'proliferative'), MO=macular oedema, MA= microaneurysm, DD=disc diameter, HES= hospital eye service				

Table S4: Reference standards intra-rater agreement score using kappa statistic (first attempt versus second attempt)

	**Intra-rater agreement (reference standard, UK), k (95% CI) (by eyes, n=106)**	**Intra-rater agreement (reference standard, UK), k (95% CI) (by worst eye, n=53)**
**Overall Diabetic Retinopathy Grading:**		
**Any DR**	0.96 (0.91,1.00)	0.92 (0.82, 1.00)
**Treatable DR**	0.81 (0.60, 1.00)	0.74 (0.47, 1.00)
**Referable Maculopathy**	0.97 (0.92, 1.0)0	1.00 (1.00, 1.00)

Abbreviations: CI=confidence interval, k=kappa, DR=Diabetic retinopathy, DMO=diabetic macular oedema
Any DR defined as R1, R2, R3s, R3a and U
Treatable DR defined as R3a
Referable DMO defined as M1 and U

Table S5: Using kappa statistic to determine the inter-rater agreement between the reference standard and one senior grader from QUB grading centre

	**Inter-rater agreement (reference standard vs a senior grader QUB), k (95% CI) (by eyes, n=106)**	**Inter-rater agreement (reference standard vs a senior grader QUB (by worst), k (95% CI) (by worst eye, n=53)**
**Overall Diabetic Retinopathy Grading:**		
**Any DR**	0.79 (0.67, 0.91)	0.74 (0.55, 0.92)
**Treatable DR**	0.71 (0.48, 0.95)	0.68 (0.39, 0.97)
**Referable Maculopathy**	0.75 (0.61, 0.90)	0.74 (0.55, 0.93)

Abbreviations: CI=confidence interval, k=kappa, DR=Diabetic retinopathy, DMO=diabetic macular oedema
Any DR defined as R1, R2, R3s, R3a and U
Treatable DR defined as R3a
Referable DMO defined as M1 and U

Intra and inter-grader agreement

To ensure there was good intra-grader reliability as a reference standard, a stratified random sample of images were regraded. There was approximately one month between the first and second attempts to reduce the possibility of bias caused by memory. Additionally, inter-grader agreement was calculated using kappa to ensure there was good grading agreement between the reference standard and one senior grader from the Ophthalmic Reading Centre at QUB, Belfast. Any disagreements were discussed with

a retinal specialist until consensus was reached. Overall, the intra-grader agreement and inter-grader agreement ranged from substantial to almost perfect.

Table S6. Diagnostic test accuracy of DR graders in Vietnam against a reference standard from the UK, excluding ungradable images (Phase I)

	**Level 1 graders (n=373 patient images)**	**Level 2 graders (n=379 patient images)**	**Level 3 graders (n=240 patient images)**
**Any DR**			
Sensitivity (%) (95% CI)	47.9 (38.8, 57.2)	50.8 (41.6, 60.0)	49.0 (38.7, 59.3)
Specificity (%) (95% CI)	89.7 (85.1, 93.0)	98.8 (96.3, 99.7)	100 (96.8, 100)
PPV (%) (95% CI)	69.0 (57.9, 78.4)	95.3 (86.2, 98.8)	100 (90.6, 100)
NPV (%) (95% CI)	78.2 (72.9, 82.7)	80.9 (76.0, 85.0)	74.6 (67.8, 80.5)
**Referable DR**			
Sensitivity (%) (95% CI)	38.1 (19.0, 61.3)	28.6 (12.2, 52.3)	22.2 (7.4, 48.1)
Specificity (%) (95% CI)	98.9 (96.9, 99.6)	100 (98.7, 100)	99.5 (97.1, 99.9)
PPV (%) (95% CI)	66.7 (35.4, 88.7)	100 (51.7, 100)	80.0 (29.9, 98.9)
NPV (%) (95% CI)	96.4 (93.8, 97.9)	96.0 (93.3, 97.6)	94.0 (89.9, 96.6)
**Referable DMO**			
Sensitivity (%) (95% CI)	9.3 (3.0, 23.1)	37.2 (23.4, 53.3)	26.5 (13.5, 44.7)
Specificity (%) (95% CI)	99.1 (97.0, 99.8)	99.4 (97.5, 99.9)	100 (97.6, 100)
PPV (%) (95% CI)	57.1 (20.2, 88.2)	88.9 (63.9, 98.1)	100 (62.9, 100)
NPV (%) (95% CI)	88.9 (85.1, 92.0)	92.3 (88.9, 94.7)	88.6 (83.5, 92.4)

Abbreviations: UK = United Kingdom, DR = Diabetic Retinopathy, DMO = Diabetic Macular Oedema, CI = Confidence Intervals,
Grading criteria: UK National Diabetic Eye Screening Programme (NDESP) classification system (See supplementary material, Table S1 for more details).
Any DR, is defined as grades R1, R2, R3s and R3a.
Referable DR is defined as grades R2 and R3a.
Referable DMO is defined as grades M1
Sensitivity is the ability of a test to correctly identify patients with a disease and specificity is the ability of a test to correctly identify people without the disease
Positive predictive value (PPV) is the proportion of those who test positive who have the condition (true positives) and negative predictive value (NPV) is the proportion of those who test negative who do not have the condition (true negatives).

Table S7: Diagnostic test accuracy of DR graders in Vietnam against a reference standard from the UK after additional DR training was delivered, excluding ungradable images (Phase II)

	**Level 1 graders (n=109 patient images)**	**Level 2 graders (n=105 patient images)**	**Level 3 graders (n=58 patient images)**
**Any DR**			
Sensitivity (%) (95% CI)	97.6 (85.6, 99.9)	72.5 (55.9 84.9)	55.6 (38.1, 72.1)
Specificity (%) (95% CI)	95.6 (86.8, 99.8)	100 (93.5, 100)	100 (84.6, 100)
PPV (%) (95% CI)	93.0 (79.9, 98.2)	100 (85.4, 100)	100 (80.0, 100)
NPV (%) (95% CI)	98.5 (90.7, 99.9)	85.5 (75.2, 92.2)	57.9 (10.9, 73.2)
**Referable DR**			
Sensitivity (%) (95% CI)	88.9 (50.7, 99.4)	55.6 (22.7, 84.7)	77.8 (40.0, 97.2)
Specificity (%) (95% CI)	90.0 (81.9, 94.8)	96.9 (90.5, 99.2)	100 (92.8, 100)
PPV (%) (95% CI)	44.4 (22.4, 68.7)	62.5 (25.9, 89.8)	100 (56.1, 100)
NPV (%) (95% CI)	98.9 (93.4, 99.9)	95.9 (89.2, 98.7)	96.1 (87.8, 98.8)
**Referable DMO**			
Sensitivity (%) (95% CI)	90.0 (54.1, 99.5)	60.0 (26.4, 86.3)	80.0 (44.4, 97.5)
Specificity (%) (95% CI)	97.0 (91.8, 99.2)	97.9 (91.9, 99.6)	100 (92.6, 100)
PPV (%) (95% CI)	75.0 (42.8, 93.3)	75.0 (35.6, 95.5)	100 (59.8, 100)
NPV (%) (95% CI)	99.0 (93.6, 99.9)	95.9 (89.2, 98.7)	96.0 (87.4, 99.6)

Abbreviations: UK = United Kingdom, DR = Diabetic Retinopathy, DMO = Diabetic Macular Oedema, CI = Confidence Intervals,
Grading criteria: UK National Diabetic Eye Screening Programme (NDESP) classification system (See supplementary material, Table S1 for more details).
Criteria:
Any DR is defined as grades R1, R2, R3s, and R3a.
Referable DR is defined as grades R2 and R3a.
Referable DMO is defined as grades M1.
Sensitivity is the ability of a test to correctly identify patients with a disease and specificity is the ability of a test to correctly identify people without the disease
Positive predictive value (PPV) is the proportion of those who test positive who have the condition (true positives) and negative predictive value (NPV) is the proportion of those who test negative who do not have the condition (true negatives).

Table S8: The prevalence of any diabetic retinopathy (DR), referable DR, any maculopathy and ungradable cases with the reference grader from Phase I and Phase II

Diabetic Retinopathy grades	Phase I	Phase II (post remedial training)	P-Value
R0 (n,%)	257 (62.68)	68 (59.13)	P=0.347
R1 (n,%)	100 (24.39)	32 (27.83)	
R2 (n,%)	11 (2.68)	2 (1.74)	
R3a (n,%)	10 (2.44)	7 (6.09)	
R3s (n,%)	1 (0.24)	0 (0.00)	
U (n,%)	31 (7.56)	6 (5.22)	
**Any DR**			
- Yes (n,%)	153 (37.32)	47 (40.87)	P=0.488
- No (n,%)	257 (62.68)	68(59.12)	
**Referable DR**			
- Yes (n,%)	52 (12.68)	15 (13.04)	P=0.918
- No (n,%)	358 (87.32)	100 (86.96)	
**Any DMO**			
- M0 (n,%)	324 (79.02)	99 (86.09)	
- M1 (n,%)	43 (10.49)	10 (8.70)	P=0.173
- U (n,%)	43 (10.49)	6 (5.22)	

Abbreviations: DR=diabetic retinopathy, DMO=Diabetic Macular Oedema, U=ungradable, Chi-Squares used to test significance.
