# Supplementary material for: Impact of targeted diabetic retinopathy training for graders in Vietnam and the implications for future diabetic retinopathy screening programmes: a diagnostic test accuracy study
Source: BMJ Open. 2022 Sep 9;12(9):e059205. doi: 10.1136/bmjopen-2021-059205 (PMC9472142; doi:10.1136/bmjopen-2021-059205)
Supplement: Author's manuscript [file bmjopen-2021-059205.draft_revisions.pdf]

BMJ Open is committed to open peer review. As part of this commitment we make the peer review history of every article we publish publicly available.

When an article is published we post the peer reviewers' comments and the authors' responses online. We also post the versions of the paper that were used during peer review. These are the versions that the peer review comments apply to.

The versions of the paper that follow are the versions that were submitted during the peer review process. They are not the versions of record or the final published versions. They should not be cited or distributed as the published version of this manuscript.

BMJ Open is an open access journal and the full, final, typeset and author-corrected version of record of the manuscript is available on our site with no access controls, subscription charges or pay-per-view fees (http://bmjopen.bmj.com).

If you have any questions on BMJ Open's open peer review process please email info.bmjopen@bmj.com

BMJ Open

# The impact of targeted diabetic retinopathy training for graders in Vietnam and the implications for future diabetic retinopathy screening programmes

| | |
|---|---|
| Journal: | *BMJ Open* |
| Manuscript ID | bmjopen-2021-059205 |
| Article Type: | Original research |
| Date Submitted by the Author: | 11-Nov-2021 |
| Complete List of Authors: | Curran, Katie; Queen's University Belfast School of Medicine Dentistry and Biomedical Sciences, Centre of Public Health<br>Congdon, Nathan; Queen’s University of Belfast; ORBIS International<br>Hoang, Tung; Hanoi Medical University, Department of Ophthalmology; The University of Sydney Save Sight Institute<br>Lohfeld, Lynne; Queen’s University of Belfast; Eye Hospital of Wenzhou Medical University<br>Nguyen, Van; Orbis International in Vietnam<br>Nguyen, Hue; Orbis International in Vietnam<br>Nguyen, Quan; Ho Chi Minh Eye Hospital, Vitreo-Retina Department<br>Dardis, Catherine; Belfast Health and Social Care Trust, Department of Ophthalmology<br>Virgili, Gianni ; Queen’s University of Belfast; Belfast Health and Social Care Trust, Department of Ophthalmology<br>Piyasena, Prabhath; Queen’s University of Belfast<br>Tran, Huong; Orbis International in Vietnam<br>Salongcay, Recivall; Queen's University Belfast<br>Tung, Mai; Ho Chi Minh Eye Hospital, Vitreo-Retina Department<br>Peto, Tunde; Queen’s University of Belfast; Belfast Health and Social Care Trust, Department of Ophthalmology |
| Keywords: | Diabetic retinopathy < DIABETES & ENDOCRINOLOGY, OPHTHALMOLOGY, PUBLIC HEALTH, Quality in health care < HEALTH SERVICES ADMINISTRATION & MANAGEMENT |
| | |

SCHOLARONE™
Manuscripts

I, the Submitting Author has the right to grant and does grant on behalf of all authors of the Work (as defined in the below author licence), an exclusive licence and/or a non-exclusive licence for contributions from authors who are: i) UK Crown employees; ii) where BMJ has agreed a CC-BY licence shall apply, and/or iii) in accordance with the terms applicable for US Federal Government officers or employees acting as part of their official duties; on a worldwide, perpetual, irrevocable, royalty-free basis to BMJ Publishing Group Ltd ("BMJ") its licensees and where the relevant Journal is co-owned by BMJ to the co-owners of the Journal, to publish the Work in this journal and any other BMJ products and to exploit all rights, as set out in our licence.*

*The Submitting Author accepts and understands that any supply made under these terms is made by BMJ to the Submitting Author unless you are acting as an employee on behalf of your employer or a postgraduate student of an affiliated institution which is paying any applicable article publishing charge ("APC") for Open Access articles. Where the Submitting Author wishes to make the Work available on an Open Access basis (and intends to pay the relevant APC), the terms of reuse of such Open Access shall be governed by a Creative Commons licence – details of these licences and which Creative Commons licence will apply to this Work are set out in our licence referred to above.*

*Other than as permitted in any relevant BMJ Author's Self Archiving Policies, I confirm this Work has not been accepted for publication elsewhere, is not being considered for publication elsewhere and does not duplicate material already published. I confirm all authors consent to publication of this Work and authorise the granting of this licence.*

For peer review only

**Title: The impact of targeted diabetic retinopathy training for graders in Vietnam and the implications for future diabetic retinopathy screening programmes**

**Short title:** Training diabetic retinopathy graders in Vietnam

**Authors:**

*Katie Curran, MPH, PhD[1] (k.curran@qub.ac.uk)

Nathan Congdon, MD, MPH[1,2,3] (Nathan.Congdon@orbis.org)

Tung Thanh Hoang[4,5] (tung.hoang@sydney.edu.au)

Lynne Lohfeld, MPH, PhD[1,6] (l.lohfeld@qub.ac.uk)

Van Thu Nguyen, BA[7] (van.nguyen@orbis.org)

Hue Thi Nguyen, MD, MPH[7] (hue.nguyen@orbis.org)

Quan Nhu Nguyen, MD, PhD[8] (bsquan2009@gmail.com)

Catherine Dardis, MSc[9] (cathy831oph@gmail.com)

Gianni Virgili MD[1,9] (G.Virgili@qub.ac.uk)

Prabhath Piyasena, MB BS, PhD[1], (m.piyasena@qub.ac.uk)

Huong Tran, MPA[7] (Huong.Tran@orbis.org)

Recivall Salongcay, MD, MPM[1] (R.SALONGCAY@qub.ac.uk)

Mai Quoc Tung, MD, PhD[8] (maitungtn@gmail.com)

For peer review only



For peer review only - http://bmjopen.bmj.com/site/about/guidelines.xhtml

Tunde Peto, MD, PhD[1,9] (T.Peto@qub.ac.uk)

**Affiliations:**

[1]Centre for Public Health, Queen's University of Belfast, Belfast, UK

[2]Orbis International, New York, USA

[3]Zhongshan Ophthalmic Centre, Guangzhou, China

[4]Ophthalmology Department, Hanoi Medical University, Hanoi, Vietnam

[5]Save Sight Institute, The University of Sydney School of Medicine, Sydney, Australia

[6]Eye Hospital of Wenzhou Medical University, Wenzhou, Zhejiang Province, China

[7]Orbis International in Vietnam, Hanoi, Vietnam

[8]Vitreo-Retina Department, Ho Chi Minh Eye Hospital, Ho Chi Minh City, Vietnam

[9]Department of Ophthalmology, Belfast Health and Social Care Trust, NI

***Corresponding author**

Katie Curran

Centre for Public Health

Institute of Clinical Sciences, A

Queen's University Belfast

BT12 6BA

Email: k.curran@qub.ac.uk

For peer review only



For peer review only - http://bmjopen.bmj.com/site/about/guidelines.xhtml

**Keywords:** Diabetic retinopathy, diabetic retinopathy training, screening

**Word Count:** 2265

**Abstract**

**Objectives:** To compare the accuracy of trained level 1 diabetic retinopathy (DR) graders (nurses, endocrinologists, one general practitioner), level 2 graders (mid-level ophthalmologists) and level 3 graders (senior ophthalmologists) in Vietnam against a reference standard from the UK, and assess the impact of supplementary targeted grader training.

**Methods:** DR training was delivered to new Vietnamese graders in February 2018 by National Health System (NHS) UK graders. Two-field retinal images were taken by trained screeners and graded by 14 trained graders in Vietnam between August-October 2018 and then re-graded retrospectively by an NHS-certified reference standard UK optometrist (Phase I). Further directed DR training based on Phase I results was delivered to Vietnamese graders between March-November 2019. After training was delivered, a randomised subset of images from January-October 2020 was graded by 6 of the original cohort (Phase II). The reference grader re-graded all images from Phase I and II retrospectively in masked fashion. Sensitivity was calculated at the two different time points and Chi Squared was used to test significance.

**Results:** In Phase I, the sensitivity for detecting any DR for all grader groups in Vietnam was low and improved in Phase II after additional training was delivered. The greatest improvement was seen among level 1 graders ($P<0.001$) and the lowest improvement was observed among level 3 graders ($P=0.326$). There was an improvement in sensitivity for detecting DR and referable diabetic macular oedema between all grader levels and whilst the



For peer review only - http://bmjopen.bmj.com/site/about/guidelines.xhtml

differences were statistically significant, the post-training values were suboptimal (41.8% to 61.5%). The main disagreement was the detection of ungradable images.

**Conclusions:** This is among the first studies to demonstrate that targeted training interventions can improve accuracy of DR grading in a low-middle income country. These findings have important implications for improving service delivery in DR screening programmes in low-resource settings.

## Article Summary

### Strengths and limitations of this study

- This is the first study describing the impact of a training intervention to improve the quality of DR grading in an LMIC.
- Reinforcing training to identify ungradable images has been acknowledged.
- The sample size was smaller in Phase II compared to Phase I, however, there were no statistically significant differences between the groups.

For peer review only



For peer review only - http://bmjopen.bmj.com/site/about/guidelines.xhtml

## Introduction

The prevalence of diabetes among adults in Vietnam is approximately 6% and has almost doubled over the past decade.[1] Early detection through diabetic eye screening programmes (DESPs) is important to reduce the risk of avoidable blindness due to diabetic retinopathy (DR). Since the introduction of systematic DESPs in the UK, a high-income country (HIC), diabetic retinopathy (DR) is no longer the leading cause of blindness among working age adults.[2] The key to such successful DESPs is implementing accurate, innovative and cost-effective models tailored to fit healthcare systems and contexts.

Investing in training personnel to increase human resources and procuring appropriate diagnostic and treatment equipment are essential to ensure that service providers can deliver optimum care for people with DR. In low-middle income countries (LMICs), there is often insufficient capacity to implement robust DESPs due to the lack of skilled human resources and infrastructure.[3,4] In Vietnam, there are only 14 ophthalmologists per million population compared to 49 per million in the UK.[5]

All screening programmes must provide evidence of their ability to detect the targeted condition and ensure that the service performs efficiently to improve screening accuracy when it falls short. To date, there is insufficient evidence on DR grading accuracy using non-mydriatic digital imaging by trained graders in LMICs, and even less about the capacity of DESPs in LMICs to improve where poor accuracy is detected. The current retrospective study is designed to assess accuracy of a range of graders in a non-governmental organisation (NGO)-supported DESP in Vietnam, and to study the efficacy of a quality-improvement intervention.

## Methods



For peer review only - http://bmjopen.bmj.com/site/about/guidelines.xhtml

**Study participants**: The 14 participants from Vietnam in Phase I included: Level 1 DR graders (6 nurses, 1 general practitioner and 2 endocrinologists, all with < 1 year grading experience, 55.6% female), Level 2 DR graders (3 newly-qualified ophthalmologists with < 1 year formal DR grading experience, 100% female), and Level 2 DR graders (2 senior ophthalmologists with >5 years' experience providing treatment for sight threatening DR, but with <1 year formal DR grading experience, 100% male). In Phase II, 6/14 graders (3 Level 1, 2 Level 2, 1 Level 3) from Phase I were included. The reference standard from the UK (KC) was a fully-qualified optometrist trained in DR grading and certified by the UK NHS DESP.[6] Vietnamese Level 1, 2 and 3 graders are equivalent to primary, secondary and arbitration graders, respectively, in UK DESPs.[7] In the current study, Vietnamese Level 1 and Level 2 graders graded all fundus images for DR. All images having disagreement between graders, and an additional randomly-selected 40% of all images, were sent for arbitration grading by Level 3 graders in Vietnam. All graders in Vietnam were masked to any prior diagnoses or grades of the reference standard, while the reference standard was also masked to results of grading in Vietnam.

**DR training for graders in Vietnam:** As part of a DESP project supported by NGO Orbis International, a team of five Vietnamese doctors and medical administrators visited a Northern Ireland (NI) DESP in September 2017 to receive training on screening, programme administration and quality control methods. In February 2018, a senior UK NHS grader from the Belfast Trust (CD) and a fully-qualified optometrist, trained in DR grading and certified by the NHS (KC), visited Vietnam to deliver DR training to graders involved in the DESPs. Training focused on ocular anatomy, retinal diseases, DR signs and grading (based on the UK National Screening Committee (NSC) classification system), and appropriate referral pathways and management.[8]



For peer review only - http://bmjopen.bmj.com/site/about/guidelines.xhtml

**Image acquisition and management:** Images were captured by trained nurses and technicians in Vietnam. Two-field, 45° digital colour photographs (one disc-centred and one macula-centred) were taken using a non-mydriatic camera (Canon CR2-AF, Canon Medical Systems. Europe), in accordance with the UK's NHS DESP.[9] Nurses and technicians were trained to repeat inadequate images as a quality control measure and take anterior segment photographs where adequate fundus images were not possible. Images were anonymised and uploaded to a cloud-based software system (Spectra)® for analysis by trained DR graders in Vietnam. The images were transferred to a Queen's University Belfast (QUB) server for re-grading by the reference standard.

**Assessment of gradeability:** Image quality was defined as 'adequate' or 'inadequate' in accordance with NHS DESP guidelines as outlined below;

- Adequate disc-centred image: complete optic disc >2DD from edge of image and fine vessels visible on surface of the disc.[9]
- Adequate macula-centred image: centre of fovea >2DD from edge of image and vessels visible within 1DD of centre of fovea.[9]

The disc-centred and macula-centred images for each eye were viewed as a pair and graded at an individual eye level. The presence of DR and diabetic macular oedema (DMO) was also determined at a patient level and based on the worst affected eye. Ungradable images were referred for further slit-lamp examination. Where images were considered inadequate but referable disease was detectable, the referable grade was recorded and the patients were moved onto the appropriate referable grade pathway.[9]

Consecutive patients diagnosed with diabetes and undergoing evaluation for possible DR at Ho Chi Minh City General Hospital and Ho Chi Minh Eye Hospital (tertiary hospitals), Tien



For peer review only - http://bmjopen.bmj.com/site/about/guidelines.xhtml

Giang General Hospital (provincial hospital) and Cai Ba General Hospital (district hospital) in Vietnam were recruited. Fundus images from August to October 2018 (Phase I) were graded by 14 graders in Vietnam and then re-graded retrospectively by a reference standard from the UK in Phase I. Targeted remedial training, based on specific findings from the Phase I analysis, was delivered in March 2019 and November 2019 by UK graders and Orbis. Additionally, regular testing and training for quality assurance purposes was also introduced, similar to UK DESP models. To evaluate the impact of this quality-improvement intervention, a new subset of images was graded by six of the original cohort of graders between January-October 2020 (Phase II) and re-graded by the reference standard from the UK (KC) in September 2021.

**Statistical analysis:** Data were entered into Microsoft Excel version 16.0 and then transferred to Stata 16.0 (StataCorp LLC) for analysis. Intra and inter-grader agreement was calculated using kappa and a stratified random sampling technique was utilised to ensure a representative sample of images was re-graded (Supplementary Files S1 and S2). Diagnostic test accuracy (DTA) comparing graders in Vietnam with the UK reference standard was assessed by calculating sensitivity, specificity, positive predicative values (PPV) and negative predictive values (NPV). Sensitivity was calculated at the two different time points (Phase I and Phase II) and Chi Squared was used to test significance.

Patients or the public were not involved in the design, or conduct, or reporting, or dissemination plans of our research.

## Results

**Patient characteristics:** In Phase I, 65.4% of patients were female with a mean age 59.4 years. In Phase II, 40.0% were female with a mean age of 59.8 years. Figures 1 and 2



For peer review only - http://bmjopen.bmj.com/site/about/guidelines.xhtml

describe enrolment of patients and capture and grading of images in Phase I and II of the study respectively.

Figure 1



For peer review only - http://bmjopen.bmj.com/site/about/guidelines.xhtml

Figure 2

For peer review only



For peer review only - http://bmjopen.bmj.com/site/about/guidelines.xhtml

173 **Initial grading performance analysis (Phase I):** The sensitivity for detecting any DR was low against the reference standard in the UK for all grader groups in Vietnam. The sensitivity for detecting referable DR and referable DMO was even lower for all grader groups (Table 1).

177 **Table 1. Diagnostic test accuracy of DR graders in Vietnam against a reference standard from the UK, including ungradable images.**

| | **Level 1 graders (n=410 patient images)*** | **Level 2 graders (n=410 patient images)*** | **Level 3 graders (n=260 patient images )†** |
|---|---|---|---|
| **Any DR** | | | |
| Sensitivity (%) (95% CI) | 41.8 (33.9, 50.1) | 42.5 (34.5, 50.7) | 42.2 (33.1, 51.8) |
| Specificity (%) (95% CI) | 87.9 (83.3, 91.7) | 98.8 (96.6, 99.8) | 100 (97.5, 100) |
| PPV (%) (95% CI) | 67.4 (57.0, 76.6) | 95.6 (87.6, 99.1) | 100 (92.7, 100) |
| NPV (%) (95% CI) | 71.7 (66.4, 76.7) | 74.3 (69.3, 78.8) | 68.2 (61.5, 74.5) |
| **Referable DR** | | | |
| Sensitivity (%) (95% CI) | 19.2 (9.63, 32.5) | 13.5 (5.59, 25.8) | 10.5 (2.94, 24.8) |
| Specificity (%) (95% CI) | 97.2 (94.9, 98.7) | 100 (99.0, 100) | 99.5 (97.5, 100) |
| PPV (%) (95% CI) | 50.0 (27.2, 72.8) | 100 (59.0, 100) | 80.0 (28.4, 99.5) |
| NPV (%) (95% CI) | 89.2 (85.7, 92.1) | 88.8 (85.3, 91.7) | 86.7 (81.9, 90.6) |
| **Referable DMO** | | | |
| Sensitivity (%) (95% CI) | 5.8 (1.91, 13.0) | 20.9 (12.9, 31.0) | 16.9 (8.76, 28.3) |
| Specificity (%) (95% CI) | 97.2 (94.8, 98.7) | 99.4 (97.8, 99.9) | 100 (98.1, 100) |
| PPV (%) (95% CI) | 35.7 (12.8, 64.9) | 90.0 (68.3, 98.8) | 100 (71.5, 100) |
| NPV (%) (95% CI) | 79.5 (75.2, 83.4) | 82.6 (78.4, 86.2) | 78.3 (72.7, 83.3) |

**Abbreviations:** UK = United Kingdom, DR = Diabetic Retinopathy, DMO = Diabetic Macular Oedema, CI = Confidence Intervals,
Grading criteria: UK National Diabetic Eye Screening Programme (NDESP) classification system.
Criteria:
Any DR, is defined as grades R1, R2, R3s, R3a and U.
Referable DR is defined as grades R2, R3a and U
Referable DMO is defined as grades M1 and U
*Missing (n=2, 0.5%), †missing (n=2, 0.8%)

179



For peer review only - http://bmjopen.bmj.com/site/about/guidelines.xhtml

**Subsequent grading performance after retraining (Phase II):** Subsequently, a further subset of images from 115 consecutive patients from January to October 2020 were graded by 6 of the original cohort of 14 Vietnamese graders, and were regraded in the UK to evaluate graders' performance after targeted training was delivered and quality control measures were instituted. The greatest improvement in sensitivity for detecting any DR was seen among level 1 graders (difference: +45.4%, 95%CI +33.1% to +57.8%; P<0.001). The specificity increased from 87.9% in phase I to 95.6% in phase II which helps to avoid over referrals (difference: +7.7%, 95%CI +1.4% to +13.9%; p=0.069). The lowest improvement in sensitivity for detecting any DR was observed between level 3 graders in Vietnam (difference; +9.0%, 95%CI: -9.0% to +27.1%; p=0.326), although their specificity remained 100% at phase I and phase II. There was an improvement in sensitivity for detecting DR and referable DMO between all grader levels and whilst there were statistically significant differences, sensitivities after training were still insufficient and comprised between about 40% and 61.5% (Table 2).

**Table 2: Diagnostic test accuracy of DR graders in Vietnam against a reference standard from the UK after additional DR training was delivered.**

| | **Level 1 graders (n=115 patient images)** | **Level 2 graders (n=115 patient images)** | **Level 3 graders (n=62 patient images)** |
|---|---|---|---|
| **Any DR** | | | |
| Sensitivity (%) (95% CI) | 87.2 (74.3, 95.2) | 68.1 (52.9, 80.9) | 51.3 (34.8, 67.6) |
| Specificity (%) (95% CI) | 95.6 (87.6, 99.1) | 95.6 (87.6, 99.1) | 100 (84.6, 100) |
| PPV (%) (95% CI) | 93.2 (81.3, 98.6) | 91.4 (76.9, 98.2) | 100 (83.2, 100) |
| NPV (%) (95% CI) | 91.5 (82.5, 96.8) | 81.3 (71.0, 89.1) | 53.7 (37.4, 69.3) |
| P-value comparing sensitivity to Phase I | P=0.000 | P=0.002 | P=0.326 |
| **Referable DR** | | | |
| Sensitivity (%) (95% CI) | 53.3 (26.6, 78.7) | 40.0 (16.3, 67.7) | 58.3 (27.7, 84.8) |
| Specificity (%) (95% CI) | 90.0 (82.4, 95.1) | 93.0 (86.1, 97.1) | 100 (92.7, 100) |
| PPV (%) (95% CI) | 44.4 (21.5, 69.2) | 46.2 (19.2, 74.9) | 100 (59.0, 100) |
| NPV (%) (95% CI) | 92.8 (85.7, 97.0) | 91.2 (83, 95.9) | 90.7 (79.7, 96.9) |
| P-value comparing sensitivity to Phase I | P=0.009 | P=0.022 | P=0.001 |
| **Referable DMO** | | | |

For peer review only

| | | | |
|---|---|---|---|
| Sensitivity (%) (95% CI) | 56.3 (29.9, 80.2) | 43.8 (19.8, 70.1) | 61.5 (31.6, 86.1) |
| Specificity (%) (95% CI) | 97.0 (91.4, 99.4) | 93.9 (87.3, 97.7) | 100 (92.6, 100) |
| PPV (%) (95% CI) | 75.0 (42.8 94.5) | 53.8 (25.1, 80.8) | 100 (63.1, 100) |
| NPV (%) (95% CI) | 93.2 (86.5, 97.2) | 91.2 (83.9, 95.9) | 90.6 (79.3, 96.9) |
| P-value comparing sensitivity to Phase I | P=0.000 | P=0.051 | P=0.002 |

**Abbreviations:** UK = United Kingdom, DR = Diabetic Retinopathy, DMO = Diabetic Macular Oedema, CI = Confidence Intervals,
Grading criteria: UK National Diabetic Eye Screening Programme (NDESP) classification system.
Criteria:
Any DR is defined as grades R1, R2, R3s, R3a and U.
Referable DR is defined as grades R2, R3a and U
Referable DMO is defined as grades M1 and U
Chi-squared used to compare sensitivity between Phase I and II.

The main discordance is detecting ungradable images, which does not improve much with training; therefore, training should be reinforced to ungradable images for the future (Supplementary File S3).

## Discussion

Results from our study demonstrate extremely poor sensitivity and specificity for detecting all levels of DR, especially referable DR, in the early stages of programme delivery. This translates into increased costs to the health care system due to missed opportunities for early treatment and un-necessary examinations for false-positive referrals. The quality of patient care also suffers. Didactic DR training was delivered to graders in Vietnam over a two-year period by trained DR graders from the UK and Vietnam. Training was specifically targeted to address problems identified in the Phase I testing, and quality control testing using international test and training (iTAT) were also undertaken. This study demonstrates that these steps led to improved grading accuracy for all classes of patients and graders; however, results remain suboptimal for a screening programme. The main discordance was detection of ungradable images, therefore, training should be reinforced to detect ungradable images.



For peer review only - http://bmjopen.bmj.com/site/about/guidelines.xhtml

Results can be poor in these settings for a variety of reasons, quality assessment is crucial, and programmatic changes based on models such as the UK DESP can be successful in enhancing grader accuracy in LMICs settings. However, it is fundamental for countries to adapt their own DR classification system and referral pathways to meet their requirements. Most importantly, the role of affiliated hospitals (and partnerships, coordination among training institutions and practical hospitals) are crucial for DR grading quality improvement

Studies in LMICs and HICs have assessed the accuracy of non-medical graders and medical graders in the detection of DR and found that both grader types are capable of achieving moderate-high sensitivity for detecting DR.[7, 10-13] Previous studies have briefly described what training interventions were used to train their graders, although no study has outlined whether additional training methods were employed to improve grading accuracy if needed. In the UK, the DR grading course by the Gloucestershire Retinal Education Group is compulsory for graders in addition to monthly iTAT.[6] This formal training qualification and continuous monitoring and evaluation are crucial to achieve optimal sensitivity, which may be more challenging in terms of costs and capacity for LMICs.

For peer review only

**Strengths:** To our knowledge, this is the first study describing the impact of a training intervention to improve the quality of DR grading in an LMIC. The inclusion of ungradable images in this study was a logical decision, particularly when the prevalence of cataract (which often renders DR images ungradable) is high in LMICs.[10] Dense cataracts normally obstruct the view of the fundus, making it difficult to obtain clear fundus photographs and assign a DR grade. In these instances, referring patients to an eye clinic for further assessment and treatment as needed is required. Determining sensitivity and specificity at the patient level is also important from a DESP implementation perspective. In the UK and Vietnam, both eyes are typically examined for DR and a single outcome is assigned to the



For peer review only - http://bmjopen.bmj.com/site/about/guidelines.xhtml

patient, as was done here. For these reasons, we feel our analytic approach, and thus results, are relevant to these settings.

**Limitations:** Limitations for this study have also been acknowledged. Data from this study represent routine clinical practice. In daily DR screening, not all patients undergoing primary (Level 1) and secondary (Level 2) grading proceed to arbitration grading (Level 3). This means a proportion of images were not graded by arbitration graders as outlined in figure 1 and figure 2. Second, only 6/14 graders from Phase I were included in Phase II grading; however the distribution of grader levels was similar. Third, though the proportion of patients excluded was small, we are unable to fully characterise the reasons for these exclusions, due to the nature of the study as a programmatic evaluation. Some potential reasons for this are a patient's unwillingness to participate in the study, graders having forgotten to ask for patient consent to participate in the study, and patient inability to comply with image capture. Fourth, pupil status was not recorded in this study and this can be important for LMICs. Finally, it was not practical for the UK reference standard to examine patients clinically in Vietnam; however, the method of grading by a certified DR grader or clinical specialist is widely used as the reference standard in many screening programmes.

**Conclusions:** This paper shows how grading accuracy was particularly low among all grader groups in Vietnam in the first six months of DESP implementation. Many factors may have contributed to poor grader performance, including inadequate training and feedback, insufficient time to participate in quality assurance testing and competing work responsibilities. After additional training, testing and quality assurance systems were implemented in Vietnam, DTA improved among all grader groups, however a significant amount of work is still needed. In particular, training graders to detect ungradable cases is crucial. A qualitative study to determine why the initial training intervention was less

For peer review only

successful should be explored. Since further improvements are required, understanding how other countries implement such programmes would be beneficial. Future studies should outline what DR training interventions were used, state relevant training courses and explain what quality assurance measures are in place. The findings from this study are important for DESP programme planners in Vietnam and other LMICs, highlighting the importance of quality monitoring and directed re-training as needed.

**Acknowledgements:** The authors are very grateful to all participants in Vietnam and Northern Ireland who generously gave their time for this study and to Orbis International.

**Author contributions:** All authors have made substantial contributions to the conception of the design of the work. KC and GV analysed and interpreted the data. All authors read and approved the final manuscript.

**Funding:** The project was funded by the Department for the Economy (DfE) – Global Challenges Research Fund (GCRF) Awards (Grant number N/A). None of the funders were involved in the design or conduct of the study; preparation, review or approval of the manuscript; or decision to submit the manuscript for publication. Nathan Congdon is supported by the Ulverscroft Foundation (UK). Katie Curran is funded by the Wellcome Trust.

**Competing Interests:** Nathan Congdon is employed as a Research Director by Orbis International.

**Patient consent for publication:** Not required.

**Ethics approval:** This research adhered to the tenets of the Declaration of Helsinki. Ethical approval was granted by the Hanoi Medical University Institutional Review Board in Bio-



For peer review only - http://bmjopen.bmj.com/site/about/guidelines.xhtml

Medical Research, Vietnam (No. 0518/HMU IRB). Written informed consent was obtained from all participants prior to their being interviewed.

**Data sharing statement:** All datasets relevant to the study are included in the article or uploaded as supplementary information.

**References**


1. Ngoc NB, Lin ZL, Ahmed W. Diabetes: What challenges lie ahead for Vietnam? *Ann Glob Heal.* 2020;86(1):1–9.

2. Liew G, Michaelides M, Bunce C. A comparison of the causes of blindness certifications in England and Wales in working age adults (16-64 years), 1999-2000 with 2009-2010. *BMJ* Open. 2014;4(2):1–6.

3. Burgess PI, Msukwa G, Beare NAV. Diabetic retinopathy in sub-Saharan Africa: Meeting the challenges of an emerging epidemic. *BMC Med.* 2013;11(1):1.

4. Adriono G, Wang D, Octavianus C, Congdon N. Use of eye care services among diabetic patients in urban Indonesia. *Arch Ophthalmol* (Chicago, Ill 1960). 2011;129(7):930–5.

5. International Council of Ophthalmology. Number of Ophthalmologists in Practic and Training Worldwide [Internet]. 2012 [cited 2020 Aug 13]. Available from: http://www.icoph.org/ophthalmologists-worldwide.html

6. Gloucestershire Retinal Education Group. Diabetic Retinopathy Grading Course [Internet]. 2020. [cited 2021 April] Available from: https://drscreening.org/diabetic-retinopathy-grading-course

7. Widdowson S, Taylor D. The management of grading quality: Good practice in the




For peer review only - http://bmjopen.bmj.com/site/about/guidelines.xhtml

quality assurance of grading. [Internet] 2015. [cited 2021 April] Available from: https://assets.publishing.service.gov.uk/government/uploads/system/uploads/attachment_data/file/512832/The_Management_of_Grading.pdf

8. Public Health England. NHS Diabetic Eye Screening Programme Grading definitions for referable disease. [Internet] 2021 (cited 2021 September] Available from: https://www.gov.uk/government/publications/diabetic-eye-screening-retinal-image-grading-criteria/nhs-diabetic-eye-screening-programme-grading-definitions-for-referable-disease

9. NHS DESP Core Team. Diabetic Eye Screening Programme Pathway for Adequate / Inadequate Images and where images cannot be taken Outlines pathway and business rules for image capture exceptions and ungradable images. [Internet] 2013.[cited 2021 April] Available from: https://www.gov.uk/government/publications/diabetic-eye-screening-pathway-for-images-and-where-images-cannot-be-taken

10. Murthy GVS, John N, Shamanna BR, Pant HB. Elimination of avoidable blindness due to cataract: Where do we prioritize and how should we monitor this decade. *Indian J Ophthalmol.* 2012;60(5):438–45.

11. McKenna M, Chen T, McAneney H, Membrillo MAV, Jin L, Xiao W, et al. Accuracy of trained rural ophthalmologists versus non-medical image graders in the diagnosis of diabetic retinopathy in rural China. *Br J Ophthalmol*. 2018;102(11):1471–6.

12. Amirul Islam FM. Accuracy and reliability of retinal photo grading for diabetic retinopathy: Remote graders from a developing country and standard retinal photo grader in Australia. *PLoS One.* 2017;12(6):1–12.

13. Bhargava M, Cheung CYL, Sabanayagam C, Kawasaki R, Harper CA, Lamoureux EL, et al. Accuracy of diabetic retinopathy screening by trained non-physician graders



For peer review only - http://bmjopen.bmj.com/site/about/guidelines.xhtml

using non-mydriatic fundus camera. *Singapore Med J*. 2012;53(11):715–9.

Figure 1: Flow diagram to illustrate enrolment of patients and management of images in Phase I from August to October 2018 (Initial grading performance analysis). Level 1 and level 2 graders graded the same set of photographs and level 3 graders graded a subset of these photographs: All disagreements between Level 1 and 2 graders and a 40% random sample of all images.

Figure 2: Flow diagram illustrating the enrolment of patients and management of images included in Phase II from January 2020 to October 2020 (Follow-up grading performance analysis after re-training). Level 1 and level 2 graders graded the same set of photographs and level 3 graders graded a subset of these images.

For peer review only



For peer review only - http://bmjopen.bmj.com/site/about/guidelines.xhtml

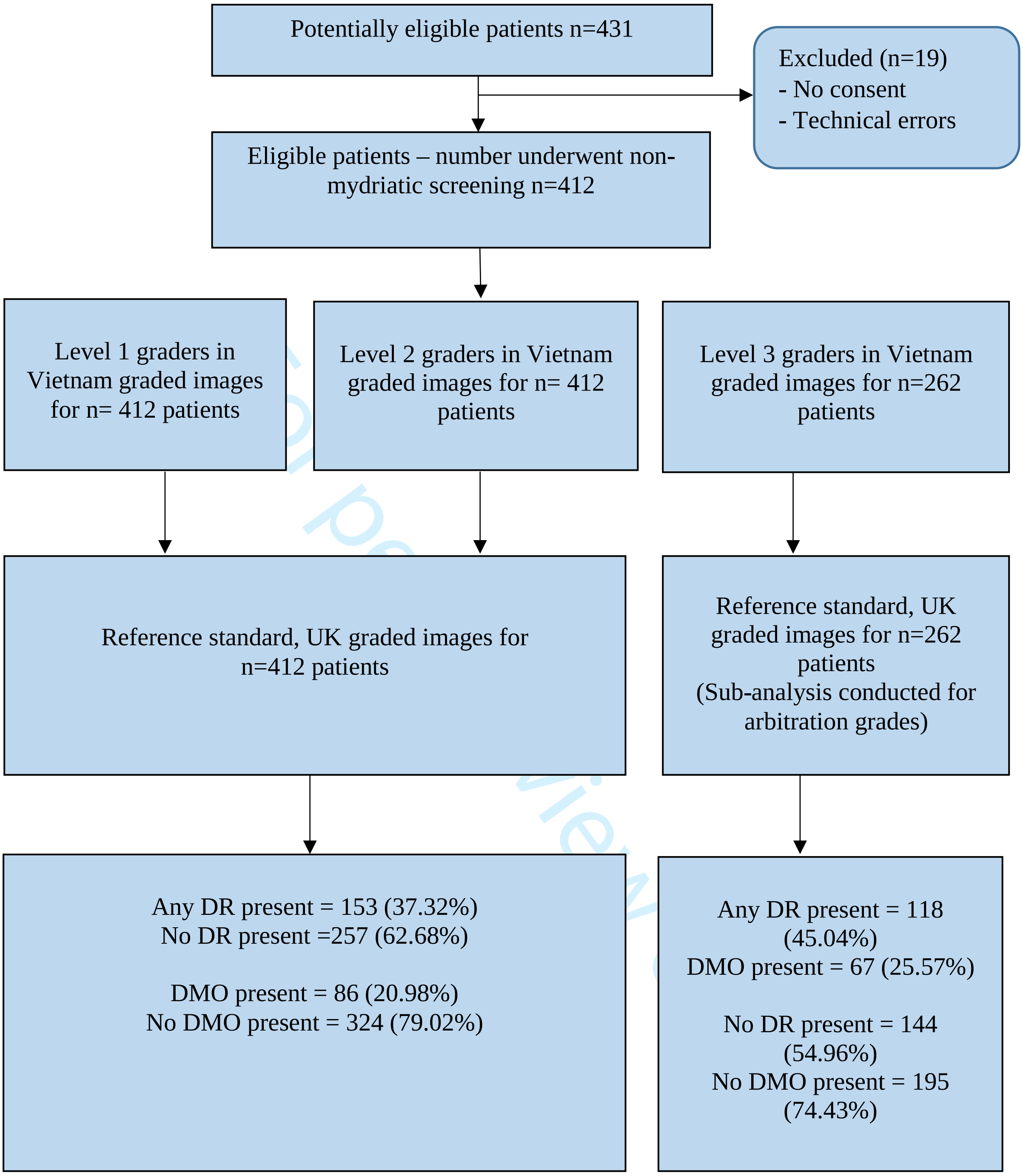


Figure 1: Flow diagram to illustrate enrolment of patients and management of images in Phase I from August to October 2018 (Initial grading performance analysis). Level 1 and level 2 graders graded the same set of photographs and level 3 graders graded a subset of these photographs: All disagreements between Level 1 and 2 graders and a 40% random sample of all images.

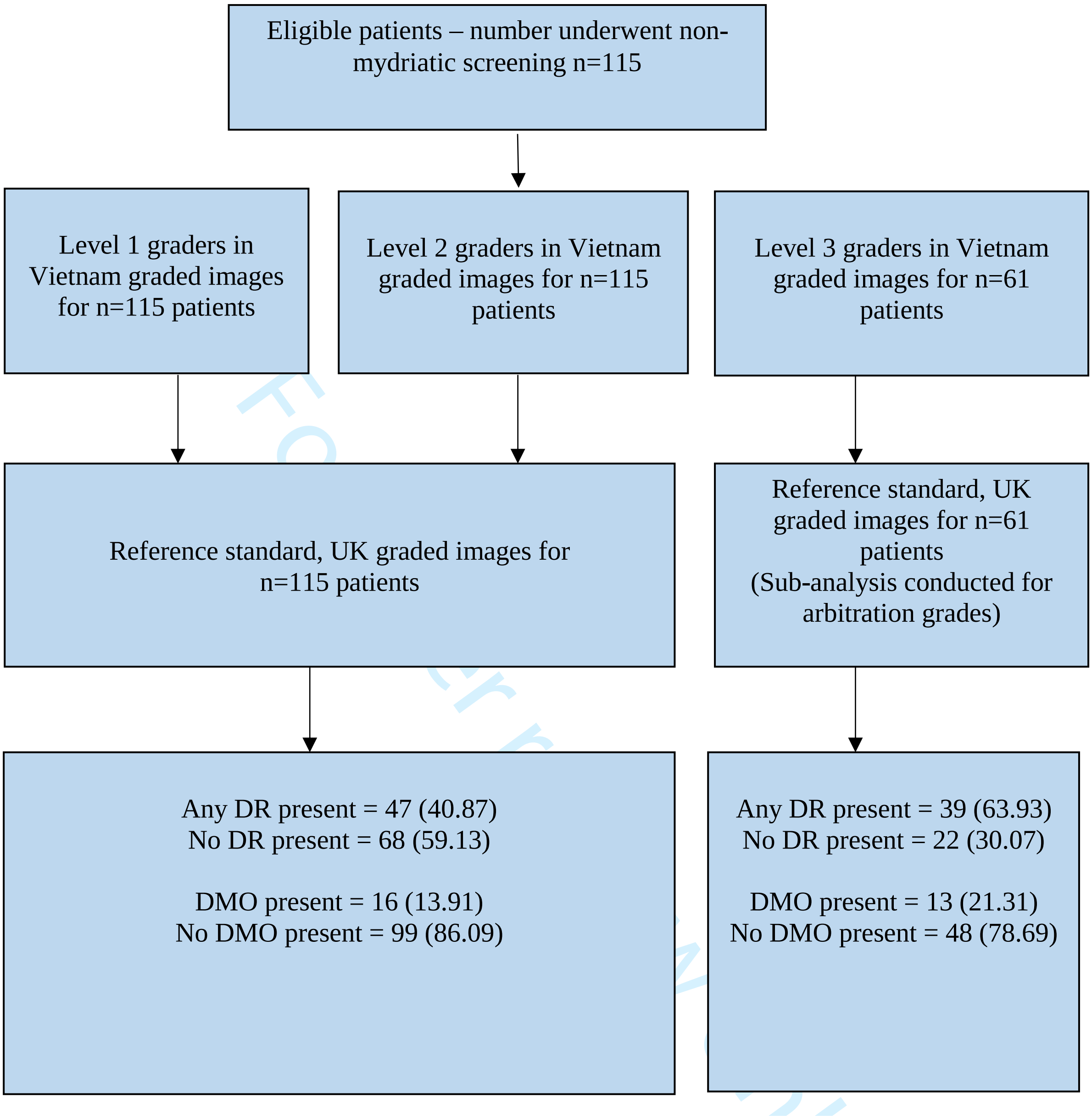


Figure 2: Flow diagram illustrating the enrolment of patients and management of images included in Phase II from January 2020 to October 2020 (Follow-up grading performance analysis after re-training). Level 1 and level 2 graders graded the same set of photographs and level 3 graders graded a subset of these images.

**Supplementary Material**

**Table S1: Reference standards intra-rater agreement score using kappa statistic (first attempt versus second attempt)**

| | **Intra-rater agreement (reference standard, UK), k (95% CI) (by eyes, n=106)** | **Intra-rater agreement (reference standard, UK), k (95% CI) (by worst eye, n=53)** |
|---|---|---|
| **Overall Diabetic Retinopathy Grading:** | | |
| **Any DR** | 0.96 (0.91,1.00) | 0.92 (0.82, 1.00) |
| **Treatable DR** | 0.81 (0.60, 1.00) | 0.74 (0.47, 1.00) |
| **Referable Maculopathy** | 0.97 (0.92, 1.0)0 | 1.00 (1.00, 1.00) |
| Abbreviations: CI=confidence interval, k=kappa, DR=Diabetic retinopathy, DMO=diabetic macular oedema<br>Any DR defined as R1, R2, R3s, R3a and U<br>Treatable DR defined as R3a<br>Referable DMO defined as M1 and U | | |

**Table S2 Using kappa statistic to determine the inter-rater agreement between the reference standard and one senior grader from QUB grading centre**

| | **Inter-rater agreement (reference standard vs a senior grader QUB), k (95% CI) (by eyes, n=106)** | **Inter-rater agreement (reference standard vs a senior grader QUB (by worst), k (95% CI) (by worst eye, n=53)** |
|---|---|---|
| **Overall Diabetic Retinopathy Grading:** | | |
| **Any DR** | 0.79 (0.67, 0.91) | 0.74 (0.55, 0.92) |
| **Treatable DR** | 0.71 (0.48, 0.95) | 0.68 (0.39, 0.97) |
| **Referable Maculopathy** | 0.75 (0.61, 0.90) | 0.74 (0.55, 0.93) |
| Abbreviations: CI=confidence interval, k=kappa, DR=Diabetic retinopathy, DMO=diabetic macular oedema<br>Any DR defined as R1, R2, R3s, R3a and U<br>Treatable DR defined as R3a<br>Referable DMO defined as M1 and U | | |

**Intra and inter-grader agreement**

To ensure there was good intra-grader reliability as a reference standard, a stratified random sample of images were regraded. There was approximately one month between the first and second attempts to reduce the possibility of bias caused by memory. Additionally, inter-grader agreement was calculated using kappa to ensure there was good grading agreement between the reference standard and one senior grader from the Ophthalmic Reading Centre at QUB, Belfast. Any disagreements were discussed with

For peer review only

a retinal specialist until consensus was reached. Overall, the intra-grader agreement and inter-grader agreement ranged from substantial to almost perfect.

**Table S3: The prevalence of any diabetic retinopathy (DR), referable DR, any maculopathy and ungradable cases with the reference grader from Phase I and Phase II**

| Diabetic Retinopathy grades | Phase I | Phase II (post remedial training) | P-Value |
|---|---|---|---|
| R0 (n,%) | 257 (62.68) | 68 (59.13) | P=0.347 |
| R1 (n,%) | 100 (24.39) | 32 (27.83) | |
| R2 (n,%) | 11 (2.68) | 2 (1.74) | |
| R3a (n,%) | 10 (2.44) | 7 (6.09) | |
| R3s (n,%) | 1 (0.24) | 0 (0.00) | |
| U (n,%) | 31 (7.56) | 6 (5.22) | |
| **Any DR** | | | |
| - Yes (n,%) | 153 (37.32) | 47 (40.87) | P=0.488 |
| - No (n,%) | 257 (62.68) | 68(59.12) | |
| **Referable DR** | | | |
| - Yes (n,%) | 52 (12.68) | 15 (13.04) | P=0.918 |
| - No (n,%) | 358 (87.32) | 100 (86.96) | |
| **Any DMO** | | | |
| - M0 (n,%) | 324 (79.02) | 99 (86.09) | |
| - M1 (n,%) | 43 (10.49) | 10 (8.70) | P=0.173 |
| - U (n,%) | 43 (10.49) | 6 (5.22) | |
| **Abbreviations:** DR=diabetic retinopathy, DMO=Diabetic Macular Oedema, U=ungradable<br>Chi-Squares used to test significance. | | | |

For peer review only

| Section & Topic | No | Item | Reported on page # |
|---|---|---|---|
| **TITLE OR ABSTRACT** | | | |
| | 1 | Identification as a study of diagnostic accuracy using at least one measure of accuracy (such as sensitivity, specificity, predictive values, or AUC) | 1 |
| **ABSTRACT** | | | |
| | 2 | Structured summary of study design, methods, results, and conclusions (for specific guidance, see STARD for Abstracts) | 3-4 |
| **INTRODUCTION** | | | |
| | 3 | Scientific and clinical background, including the intended use and clinical role of the index test | 5-6 |
| | 4 | Study objectives and hypotheses | 5 |
| **METHODS** | | | |
| *Study design* | 5 | Whether data collection was planned before the index test and reference standard were performed (prospective study) or after (retrospective study) | 5 |
| *Participants* | 6 | Eligibility criteria | 5-6 |
| | 7 | On what basis potentially eligible participants were identified (such as symptoms, results from previous tests, inclusion in registry) | 5-6 |
| | 8 | Where and when potentially eligible participants were identified (setting, location and dates) | 5-6 |
| | 9 | Whether participants formed a consecutive, random or convenience series | 6 |
| *Test methods* | 10a | Index test, in sufficient detail to allow replication | 5-6 |
| | 10b | Reference standard, in sufficient detail to allow replication | 5-6 |
| | 11 | Rationale for choosing the reference standard (if alternatives exist) | 5-6 |
| | 12a | Definition of and rationale for test positivity cut-offs or result categories of the index test, distinguishing pre-specified from exploratory | 8 |
| | 12b | Definition of and rationale for test positivity cut-offs or result categories of the reference standard, distinguishing pre-specified from exploratory | 8 |
| | 13a | Whether clinical information and reference standard results were available to the performers/readers of the index test | 6 |
| | 13b | Whether clinical information and index test results were available to the assessors of the reference standard | 6 |
| *Analysis* | 14 | Methods for estimating or comparing measures of diagnostic accuracy | 8 |
| | 15 | How indeterminate index test or reference standard results were handled | Supplementary file |
| | 16 | How missing data on the index test and reference standard were handled | NA |
| | 17 | Any analyses of variability in diagnostic accuracy, distinguishing pre-specified from exploratory | 8 |
| | 18 | Intended sample size and how it was determined | NA |
| **RESULTS** | | | |
| *Participants* | 19 | Flow of participants, using a diagram | 9-10 |
| | 20 | Baseline demographic and clinical characteristics of participants | 5-6,8 |
| | 21a | Distribution of severity of disease in those with the target condition | 9-10, Supplementary file |
| | 21b | Distribution of alternative diagnoses in those without the target condition | 9-10, Supplementary file |
| | 22 | Time interval and any clinical interventions between index test and reference standard | 6-7 |
| *Test results* | 23 | Cross tabulation of the index test results (or their distribution) by the results of the reference standard | 10-11 |
| | 24 | Estimates of diagnostic accuracy and their precision (such as 95% confidence intervals) | 10-11 |
| | 25 | Any adverse events from performing the index test or the reference standard | NA |
| **DISCUSSION** | | | |
| | 26 | Study limitations, including sources of potential bias, statistical uncertainty, and generalisability | 15 |
| | 27 | Implications for practice, including the intended use and clinical role of the index test | 15-16 |
| **OTHER INFORMATION** | | | |
| | 28 | Registration number and name of registry | NA |

For peer review only - http://bmjopen.bmj.com/site/about/guidelines.xhtml

cc BY SA

| | | | |
|---|---|---|---|
| | **29** | Where the full study protocol can be accessed | NA |
| | **30** | Sources of funding and other support; role of funders | 16 |

For peer review only

BMJ Open

# The impact of targeted diabetic retinopathy training for graders in Vietnam and the implications for future diabetic retinopathy screening programmes: a diagnostic test accuracy study

| | |
|---|---|
| Journal: | *BMJ Open* |
| Manuscript ID | bmjopen-2021-059205.R1 |
| Article Type: | Original research |
| Date Submitted by the Author: | 13-May-2022 |
| Complete List of Authors: | Curran, Katie; Queen's University Belfast School of Medicine Dentistry and Biomedical Sciences, Centre of Public Health<br>Congdon, Nathan; Queen's University of Belfast; ORBIS International<br>Hoang, Tung; Hanoi Medical University, Department of Ophthalmology; The University of Sydney Save Sight Institute<br>Lohfeld, Lynne; Queen's University of Belfast; Eye Hospital of Wenzhou Medical University<br>Nguyen, Van; Orbis International in Vietnam<br>Nguyen, Hue; Orbis International in Vietnam<br>Nguyen, Quan; Ho Chi Minh Eye Hospital, Vitreo-Retina Department<br>Dardis, Catherine; Belfast Health and Social Care Trust, Department of Ophthalmology<br>Virgili, Gianni ; Queen's University of Belfast; Belfast Health and Social Care Trust, Department of Ophthalmology<br>Piyasena, Prabhath; Queen's University of Belfast<br>Tran, Huong; Orbis International in Vietnam<br>Salongcay, Recivall; Queen's University Belfast<br>Tung, Mai; Ho Chi Minh Eye Hospital, Vitreo-Retina Department<br>Peto, Tunde; Queen's University of Belfast; Belfast Health and Social Care Trust, Department of Ophthalmology |
| <b>Primary Subject Heading</b>: | Ophthalmology |
| Secondary Subject Heading: | Diabetes and endocrinology, Medical education and training |
| Keywords: | Diabetic retinopathy < DIABETES & ENDOCRINOLOGY, OPHTHALMOLOGY, PUBLIC HEALTH, Quality in health care < HEALTH SERVICES ADMINISTRATION & MANAGEMENT |
| | |

SCHOLARONE™
Manuscripts

*I, the Submitting Author has the right to grant and does grant on behalf of all authors of the Work (as defined in the below author licence), an exclusive licence and/or a non-exclusive licence for contributions from authors who are: i) UK Crown employees; ii) where BMJ has agreed a CC-BY licence shall apply, and/or iii) in accordance with the terms applicable for US Federal Government officers or employees acting as part of their official duties; on a worldwide, perpetual, irrevocable, royalty-free basis to BMJ Publishing Group Ltd ("BMJ") its licensees and where the relevant Journal is co-owned by BMJ to the co-owners of the Journal, to publish the Work in this journal and any other BMJ products and to exploit all rights, as set out in our licence.*

*The Submitting Author accepts and understands that any supply made under these terms is made by BMJ to the Submitting Author unless you are acting as an employee on behalf of your employer or a postgraduate student of an affiliated institution which is paying any applicable article publishing charge ("APC") for Open Access articles. Where the Submitting Author wishes to make the Work available on an Open Access basis (and intends to pay the relevant APC), the terms of reuse of such Open Access shall be governed by a Creative Commons licence – details of these licences and which Creative Commons licence will apply to this Work are set out in our licence referred to above.*

*Other than as permitted in any relevant BMJ Author's Self Archiving Policies, I confirm this Work has not been accepted for publication elsewhere, is not being considered for publication elsewhere and does not duplicate material already published. I confirm all authors consent to publication of this Work and authorise the granting of this licence.*

For peer review only

**Title: The impact of targeted diabetic retinopathy training for graders in Vietnam and the implications for future diabetic retinopathy screening programmes: a diagnostic test accuracy study**

**Short title:** Training diabetic retinopathy graders in Vietnam

**Authors:**

*Katie Curran, MPH, PhD[1] (k.curran@qub.ac.uk)

Nathan Congdon, MD, MPH[1,2,3] (Nathan.Congdon@orbis.org)

Tung Thanh Hoang[4,5] (tung.hoang@sydney.edu.au)

Lynne Lohfeld, MPH, PhD[1,6] (l.lohfeld@qub.ac.uk)

Van Thu Nguyen, BA[7] (van.nguyen@orbis.org)

Hue Thi Nguyen, MD, MPH[7] (hue.nguyen@orbis.org)

Quan Nhu Nguyen, MD, PhD[8] (bsquan2009@gmail.com)

Catherine Dardis, MSc[9] (cathy831oph@gmail.com)

Gianni Virgili MD[1,9] (G.Virgili@qub.ac.uk)

Prabhath Piyasena, MB BS, PhD[1], (m.piyasena@qub.ac.uk)

Huong Tran, MPA[7] (Huong.Tran@orbis.org)

Recivall Salongcay, MD, MPM[1] (R.SALONGCAY@qub.ac.uk)

Mai Quoc Tung, MD, PhD[8] (maitungtn@gmail.com)

For peer review only

Tunde Peto, MD, PhD[1,9] (T.Peto@qub.ac.uk)

**Affiliations:**

[1]Centre for Public Health, Queen's University of Belfast, Belfast, UK

[2]Orbis International, New York, USA

[3]Zhongshan Ophthalmic Centre, Guangzhou, China

[4]Ophthalmology Department, Hanoi Medical University, Hanoi, Vietnam

[5]Save Sight Institute, The University of Sydney School of Medicine, Sydney, Australia

[6]Eye Hospital of Wenzhou Medical University, Wenzhou, Zhejiang Province, China

[7]Orbis International in Vietnam, Hanoi, Vietnam

[8]Vitreo-Retina Department, Ho Chi Minh Eye Hospital, Ho Chi Minh City, Vietnam

[9]Department of Ophthalmology, Belfast Health and Social Care Trust, NI

***Corresponding author**

Katie Curran

Centre for Public Health

Institute of Clinical Sciences, A

Queen's University Belfast

BT12 6BA

Email: k.curran@qub.ac.uk

For peer review only

**Keywords:** Diabetic retinopathy, diabetic retinopathy training, screening

**Word Count:** 2545

**Abstract**

**Objectives:** To compare the accuracy of trained level 1 diabetic retinopathy (DR) graders (nurses, endocrinologists, one general practitioner), level 2 graders (mid-level ophthalmologists) and level 3 graders (senior ophthalmologists) in Vietnam against a reference standard from the UK, and assess the impact of supplementary targeted grader training.

**Design:** Diagnostic test accuracy study.

**Setting:** Secondary care hospitals in Southern Vietnam

**Participants:** DR training was delivered to Vietnamese graders in February 2018 by National Health System (NHS) UK graders. Two-field retinal images (412 patient images) were graded by 14 trained graders in Vietnam between August-October 2018 and then re-graded retrospectively by an NHS-certified reference standard UK optometrist (Phase I). Further directed DR training based on Phase I results was delivered to graders in November 2019. After training, a randomised subset of images from January-October 2020 (115 patient images) was graded by 6 of the original cohort (Phase II). The reference grader re-graded all images from Phase I and II retrospectively in masked fashion.

**Primary and secondary outcome measures:** Sensitivity was calculated at the two different time points and Chi-Squared was used to test significance.



For peer review only - http://bmjopen.bmj.com/site/about/guidelines.xhtml

**Results:** In Phase I, the sensitivity for detecting any DR for all grader groups in Vietnam was low and improved in Phase II after additional training was delivered. The greatest improvement was seen among level 1 graders ($P<0.001$) and the lowest improvement was observed among level 3 graders ($P=0.326$). There was an improvement in sensitivity for detecting any DR and referable diabetic macular oedema between all grader levels and whilst the differences were statistically significant, the post-training values were suboptimal (41.8% to 61.5%). The main disagreement was the detection of ungradable images.

**Conclusions:** This study demonstrates that targeted training interventions can improve accuracy of DR grading in a low-middle income country. These findings have important implications for improving service delivery in DR screening programmes in low-resource settings.

**Article Summary**

**Strengths and limitations of this study**

- Graders in Vietnam were trained to detect DR based on the UK's DR screening model
- This study describes the impact of a training intervention to improve DR grading in Vietnam
- Reinforcing training to identify ungradable images has been acknowledged.



For peer review only - http://bmjopen.bmj.com/site/about/guidelines.xhtml

## Introduction

The prevalence of diabetes among adults in Vietnam is approximately 6% and has almost doubled over the past decade.[1] Early detection through diabetic eye screening programmes (DESPs) is important to reduce the risk of avoidable blindness due to diabetic retinopathy (DR). Since the introduction of systematic DESPs in the UK, a high-income country (HIC), diabetic retinopathy (DR) is no longer the leading cause of blindness among working age adults.[2] The key to such successful DESPs is implementing accurate, innovative and cost-effective models tailored to fit healthcare systems and contexts.

Investing in training personnel to increase human resources and procuring appropriate diagnostic and treatment equipment are essential to ensure that service providers can deliver optimum care for people with DR. In low-middle income countries (LMICs), there is often insufficient capacity to implement robust DESPs due to the lack of skilled human resources and infrastructure.[3,4] In Vietnam, there are only 14 ophthalmologists per million population compared to 49 per million in the UK.[5]

All screening programmes must provide evidence of their ability to detect the targeted condition and ensure that the service performs efficiently to improve screening accuracy when it falls short. To date, there is insufficient evidence on DR grading accuracy using non-mydriatic digital imaging by trained graders in LMICs, and even less about the capacity of DESPs in LMICs to improve where poor accuracy is detected. The current retrospective study is designed to assess accuracy of a range of graders in a non-governmental organisation (NGO)-supported DESP in Vietnam, and to study the efficacy of a quality-improvement intervention.

## Methods



For peer review only - http://bmjopen.bmj.com/site/about/guidelines.xhtml

**Study participants**: The 14 participants from Vietnam in Phase I included: Level 1 DR graders (6 nurses, 1 general practitioner and 2 endocrinologists, all with < 1 year grading experience, 55.6% female), Level 2 DR graders (3 newly-qualified ophthalmologists with < 1 year formal DR grading experience, 100% female), and Level 3 DR graders (2 senior ophthalmologists with >5 years' experience providing treatment for sight threatening DR, but with <1 year formal DR grading experience, 100% male). In Phase II, 6/14 graders (3 Level 1, 2 Level 2, 1 Level 3) from Phase I were included. The reference standard from the UK (KC) was a fully-qualified optometrist trained in DR grading and certified by the UK NHS DESP.[6] Vietnamese Level 1, 2 and 3 graders are equivalent to primary, secondary and arbitration graders, respectively, in UK DESPs.[7] In the current study, Vietnamese Level 1 and Level 2 graders graded all fundus images for DR. All images having disagreement between graders, and an additional randomly-selected 40% of all images, were sent for arbitration grading by Level 3 graders in Vietnam. All graders in Vietnam were masked to any prior diagnoses or grades of the reference standard, while the reference standard was also masked to results of grading in Vietnam. Fundus images were graded for 412 patients in phase I and 115 patients in phase II (Figure 1 and figure 2).

**DR training for graders in Vietnam:** As part of a DESP project supported by NGO Orbis International, a team of five Vietnamese doctors and medical administrators visited a Northern Ireland (NI) DESP in September 2017 to receive training on screening, programme administration and quality control methods. In February 2018, a senior UK NHS grader from the Belfast Trust (CD) and a fully-qualified optometrist, trained in DR grading and certified by the NHS (KC), visited Vietnam to deliver DR training to graders involved in the DESPs. (See supplementary material, figure S1 for training timeline).Training focused on ocular anatomy, retinal diseases, DR signs and grading (based on the UK National Screening



For peer review only - http://bmjopen.bmj.com/site/about/guidelines.xhtml

Committee (NSC) classification system), and appropriate referral pathways and management (Supplementary material, Table S1).[8]

**Image acquisition and management:** Images were captured by trained nurses and technicians in Vietnam. Two-field, 45° digital colour photographs (one disc-centred and one macula-centred) were taken using a tabletop non-mydriatic fundus camera (Canon CR2-AF, Canon Medical Systems. Europe), in accordance with the UK's NHS DESP.[9] Nurses and technicians were trained to repeat inadequate images as a quality control measure and take anterior segment photographs where adequate fundus images were not possible. Images were anonymised and uploaded to a cloud-based software system (Spectra)® for analysis by trained DR graders in Vietnam. The images were transferred to a Queen's University Belfast (QUB) server for re-grading by the reference standard.

**Assessment of gradeability:** Image quality was defined as 'adequate' or 'inadequate' in accordance with NHS DESP guidelines as outlined below;

- Adequate disc-centred image: complete optic disc >2DD from edge of image and fine vessels visible on surface of the disc.[9]
- Adequate macula-centred image: centre of fovea >2DD from edge of image and vessels visible within 1DD of centre of fovea.[9]

The disc-centred and macula-centred images for each eye were viewed as a pair and graded at an individual eye level. The presence of DR and diabetic macular oedema (DMO) was also determined at a patient level and based on the worst affected eye. Participants with ungradable images were referred for further slit-lamp examination. Where images were considered inadequate but referable disease was detectable, the referable grade was recorded and the patients were moved onto the appropriate referable grade pathway.[9]

For peer review only



For peer review only - http://bmjopen.bmj.com/site/about/guidelines.xhtml

Consecutive patients diagnosed with diabetes and undergoing evaluation for possible DR at Ho Chi Minh City General Hospital and Ho Chi Minh Eye Hospital (tertiary hospitals), Tien Giang General Hospital (provincial hospital) and Cai Ba General Hospital (district hospital) in Vietnam were recruited. Fundus images from August to October 2018 (Phase I) were graded by 14 graders in Vietnam and then re-graded retrospectively by a reference standard from the UK in Phase I. Targeted remedial training, based on specific findings from the Phase I analysis, was delivered in March 2019 and November 2019 by UK graders and Orbis. (Supplementary material, figure S1) Additionally, regular testing and training for quality assurance purposes was also introduced, similar to UK DESP models. To evaluate the impact of this quality-improvement intervention, a new subset of images was graded by six of the original cohort of graders between January-October 2020 (Phase II) and re-graded by the reference standard from the UK (KC) in September 2021.

**Statistical analysis:** Data were entered into Microsoft Excel version 16.0 and then transferred to Stata 17.0 (StataCorp LLC) for analysis. Intra and inter-grader agreement was calculated using kappa and a stratified random sampling technique was utilised to ensure a representative sample of images was re-graded (Supplementary tables S2 and S3). Diagnostic test accuracy (DTA) comparing graders in Vietnam with the UK reference standard was assessed by calculating sensitivity, specificity, positive predicative values (PPV) and negative predictive values (NPV). Sensitivity was calculated at the two different time points (Phase I and Phase II) and Chi Squared was used to test significance.

Patients or the public were not involved in the design, or conduct, or reporting, or dissemination plans of our research.

**Results**

For peer review only

**Patient characteristics:** In Phase I, 65.4% of patients were female with a mean age 59.4 years. In Phase II, 40.0% were female with a mean age of 59.8 years. Figures 1 and 2 describe enrolment of patients and capture and grading of images in Phase I and II of the study respectively.

For peer review only



For peer review only - http://bmjopen.bmj.com/site/about/guidelines.xhtml

Figure 1

For peer review only



For peer review only - http://bmjopen.bmj.com/site/about/guidelines.xhtml

177 Figure 2

178

For peer review only



For peer review only - http://bmjopen.bmj.com/site/about/guidelines.xhtml

**Initial grading performance analysis (Phase I):** The sensitivity for detecting any DR was low against the reference standard in the UK for all grader groups in Vietnam. The sensitivity for detecting referable DR and referable DMO was even lower for all grader groups (Table 1). (See supplementary table S4 for prevalence of DR and table S5 for grading performance, excluding ungradable images)

**Table 1. Diagnostic test accuracy of DR graders in Vietnam against a reference standard from the UK, including ungradable images.**

| | **Level 1 graders (n=410 patient images)*** | **Level 2 graders (n=410 patient images)*** | **Level 3 graders (n=260 patient images )†** |
|---|---|---|---|
| **Any DR** | | | |
| Sensitivity (%) (95% CI) | 41.8 (33.9, 50.1) | 42.5 (34.5, 50.7) | 42.2 (33.1, 51.8) |
| Specificity (%) (95% CI) | 87.9 (83.3, 91.7) | 98.8 (96.6, 99.8) | 100 (97.5, 100) |
| PPV (%) (95% CI) | 67.4 (57.0, 76.6) | 95.6 (87.6, 99.1) | 100 (92.7, 100) |
| NPV (%) (95% CI) | 71.7 (66.4, 76.7) | 74.3 (69.3, 78.8) | 68.2 (61.5, 74.5) |
| **Referable DR** | | | |
| Sensitivity (%) (95% CI) | 19.2 (9.63, 32.5) | 13.5 (5.59, 25.8) | 10.5 (2.94, 24.8) |
| Specificity (%) (95% CI) | 97.2 (94.9, 98.7) | 100 (99.0, 100) | 99.5 (97.5, 100) |
| PPV (%) (95% CI) | 50.0 (27.2, 72.8) | 100 (59.0, 100) | 80.0 (28.4, 99.5) |
| NPV (%) (95% CI) | 89.2 (85.7, 92.1) | 88.8 (85.3, 91.7) | 86.7 (81.9, 90.6) |
| **Referable DMO** | | | |
| Sensitivity (%) (95% CI) | 5.8 (1.91, 13.0) | 20.9 (12.9, 31.0) | 16.9 (8.76, 28.3) |
| Specificity (%) (95% CI) | 97.2 (94.8, 98.7) | 99.4 (97.8, 99.9) | 100 (98.1, 100) |
| PPV (%) (95% CI) | 35.7 (12.8, 64.9) | 90.0 (68.3, 98.8) | 100 (71.5, 100) |
| NPV (%) (95% CI) | 79.5 (75.2, 83.4) | 82.6 (78.4, 86.2) | 78.3 (72.7, 83.3) |

**Abbreviations:** UK = United Kingdom, DR = Diabetic Retinopathy, DMO = Diabetic Macular Oedema, CI = Confidence Intervals,
Grading criteria: UK National Diabetic Eye Screening Programme (NDESP) classification system (See supplementary material, Table S1 for more details).
Any DR, is defined as grades R1, R2, R3s, R3a and U.
Referable DR is defined as grades R2, R3a and U
Referable DMO is defined as grades M1 and U
*Missing (n=2, 0.5%), †missing (n=2, 0.8%)
Sensitivity is the ability of a test to correctly identify patients with a disease and specificity is the ability of a test to correctly identify people without the disease

For peer review only



For peer review only - http://bmjopen.bmj.com/site/about/guidelines.xhtml

Positive predictive value (PPV) is the proportion of those who test positive who have the condition (true positives) and negative predictive value (NPV) is the proportion of those who test negative who do not have the condition (true negatives).

**Subsequent grading performance after retraining (Phase II):** Subsequently, a further subset of images from 115 consecutive patients from January to October 2020 were graded by 6 of the original cohort of 14 Vietnamese graders, and were regraded in the UK to evaluate graders' performance after targeted training was delivered and quality control measures were instituted. The greatest improvement in sensitivity for detecting any DR was seen among level 1 graders (difference: +45.4%, 95%CI +33.1% to +57.8%; P<0.001). The specificity increased from 87.9% in phase I to 95.6% in phase II which helps to avoid over referrals (difference: +7.7%, 95%CI +1.4% to +13.9%; p=0.069). The lowest improvement in sensitivity for detecting any DR was observed between level 3 graders in Vietnam (difference; +9.0%, 95%CI: -9.0% to +27.1%; p=0.326), although their specificity remained 100% at phase I and phase II. There was an improvement in sensitivity for detecting DR and referable DMO between all grader levels and whilst there were statistically significant differences, sensitivities after training were still insufficient and comprised between about 40% and 61.5% (Table 2). (See supplementary table S4 for prevalence of DR and table S6 for grading performance, excluding ungradable images).



For peer review only - http://bmjopen.bmj.com/site/about/guidelines.xhtml

**Table 2: Diagnostic test accuracy of DR graders in Vietnam against a reference standard from the UK after additional DR training was delivered.**

| | Level 1 graders (n=115 patient images) | Level 2 graders (n=115 patient images) | Level 3 graders (n=62 patient images) |
|---|---|---|---|
| **Any DR** | | | |
| Sensitivity (%) (95% CI) | 87.2 (74.3, 95.2) | 68.1 (52.9, 80.9) | 51.3 (34.8, 67.6) |
| Specificity (%) (95% CI) | 95.6 (87.6, 99.1) | 95.6 (87.6, 99.1) | 100 (84.6, 100) |
| PPV (%) (95% CI) | 93.2 (81.3, 98.6) | 91.4 (76.9, 98.2) | 100 (83.2, 100) |
| NPV (%) (95% CI) | 91.5 (82.5, 96.8) | 81.3 (71.0, 89.1) | 53.7 (37.4, 69.3) |
| P-value comparing sensitivity to Phase I | P=0.000 | P=0.002 | P=0.326 |
| **Referable DR** | | | |
| Sensitivity (%) (95% CI) | 53.3 (26.6, 78.7) | 40.0 (16.3, 67.7) | 58.3 (27.7, 84.8) |
| Specificity (%) (95% CI) | 90.0 (82.4, 95.1) | 93.0 (86.1, 97.1) | 100 (92.7, 100) |
| PPV (%) (95% CI) | 44.4 (21.5, 69.2) | 46.2 (19.2, 74.9) | 100 (59.0, 100) |
| NPV (%) (95% CI) | 92.8 (85.7, 97.0) | 91.2 (83, 95.9) | 90.7 (79.7, 96.9) |
| P-value comparing sensitivity to Phase I | P=0.009 | P=0.022 | P=0.001 |
| **Referable DMO** | | | |
| Sensitivity (%) (95% CI) | 56.3 (29.9, 80.2) | 43.8 (19.8, 70.1) | 61.5 (31.6, 86.1) |
| Specificity (%) (95% CI) | 97.0 (91.4, 99.4) | 93.9 (87.3, 97.7) | 100 (92.6, 100) |
| PPV (%) (95% CI) | 75.0 (42.8 94.5) | 53.8 (25.1, 80.8) | 100 (63.1, 100) |
| NPV (%) (95% CI) | 93.2 (86.5, 97.2) | 91.2 (83.9, 95.9) | 90.6 (79.3, 96.9) |
| P-value comparing sensitivity to Phase I | P=0.000 | P=0.051 | P=0.002 |

**Abbreviations:** UK = United Kingdom, DR = Diabetic Retinopathy, DMO = Diabetic Macular Oedema, CI = Confidence Intervals,
Grading criteria: UK National Diabetic Eye Screening Programme (NDESP) classification system (See supplementary material, Table S1 for more details).
Criteria:
Any DR is defined as grades R1, R2, R3s, R3a and U.
Referable DR is defined as grades R2, R3a and U
Referable DMO is defined as grades M1 and U
Chi-squared used to compare sensitivity between Phase I and II.
Sensitivity is the ability of a test to correctly identify patients with a disease and specificity is the ability of a test to correctly identify people without the disease
Positive predictive value (PPV) is the proportion of those who test positive who have the condition (true positives) and negative predictive value (NPV) is the proportion of those who test negative who do not have the condition (true negatives).

## Discussion

For peer review only



For peer review only - http://bmjopen.bmj.com/site/about/guidelines.xhtml

Results from our study demonstrate extremely poor sensitivity and specificity for detecting all levels of DR, especially referable DR, in the early stages of programme delivery. This translates into increased costs to the health care system due to missed opportunities for early treatment and un-necessary examinations for false-positive referrals. The quality of patient care also suffers. Didactic DR training was delivered to graders in Vietnam over a two-year period by trained DR graders from the UK and Vietnam. Training was specifically targeted to address problems identified in the Phase I testing [10], and quality control testing using international test and training (iTAT) were also undertaken. The iTAT is an online platform offering monthly quality assurance and training for graders who work in DR screening. It is a useful platform for graders to improve their skills in the detection of DR from ophthalmic images. In the UK, it is compulsory for graders to complete monthly test sets (each set consisting of 20 retinal images with a range of DR severities). If graders fall below the agreed threshold, additional training and support is provided.[7] This study demonstrates that these steps led to improved grading accuracy for all classes of patients and graders; however, results remain suboptimal for a screening programme. We found that the main discordance was the graders' ability to detect ungradable images, therefore, targeted training must be given to ensure patients are referred to the next level (slit-lamp examination). According to the UK National Institute for Clinical Excellence (NICE) guidelines, DR screening tests must have at least 80% sensitivity and 95% specificity with a technical failure of 5% or less. These requirements may not be applicable to LMICs, especially at the start of the programme where a relatively low number of patients are being screened.

Results can be poor in these settings for a variety of reasons, quality assessment is crucial, and programmatic changes based on models such as the UK DESP can be successful in enhancing grader accuracy in LMICs settings. However, it is fundamental for countries to adapt their own DR classification system and referral pathways to meet their requirements.



For peer review only - http://bmjopen.bmj.com/site/about/guidelines.xhtml

As an example, the UK system (England, Wales and Northern Ireland) uses the grade M0 for no maculopathy and M1 for referable maculopathy. In Scotland, M0 denotes no maculopathy, M1 observable maculopathy and M2 referable maculopathy allowing some monitoring of maculopathy to take place on screening level. This reduces the burden on the hospital system. The implication for LMICs is that being aware of hospital capacity at the planning stage might mean that they need to safely adapt an accepted grading system to their needs. Most importantly, the role of affiliated hospitals (and partnerships, coordination among training institutions and practical hospitals) are crucial for DR grading quality improvement.

Studies in LMICs and HICs have assessed the accuracy of non-medical graders and medical graders in the detection of DR and found that both grader types are capable of achieving moderate-high sensitivity for detecting DR.[11-15] Some studies have described what training interventions were used to train their graders and key elements may be incorporated into our training programme in the future.[15-16]

In the UK, the DR grading course by the Gloucestershire Retinal Education Group is is required for grader certication. The high costs of this course may be more challenging in LMICs due to limited funding.[6]

**Strengths:** This study describes the impact of a training intervention to improve the quality of DR grading in an LMIC. The inclusion of ungradable images in this study was a logical decision, particularly when the prevalence of cataract (which often renders DR images ungradable) is high in LMICs.[11] Dense cataracts normally obstruct the view of the fundus, making it difficult to obtain clear fundus photographs and assign a DR grade. In these instances, referring patients to an eye clinic for further assessment and treatment as needed is required. Determining sensitivity and specificity at the patient level is also important from a



For peer review only - http://bmjopen.bmj.com/site/about/guidelines.xhtml

DESP implementation perspective. In the UK and Vietnam, both eyes are typically examined for DR and a single outcome is assigned to the patient, as was done here. For these reasons, we feel our analytic approach, and thus results, are relevant to these settings.

**Limitations:** Limitations for this study have also been acknowledged. Data from this study represent routine clinical practice. In daily DR screening, not all patients undergoing primary (Level 1) and secondary (Level 2) grading proceed to arbitration grading (Level 3). This means a proportion of images were not graded by arbitration graders as outlined in figure 1 and figure 2. Second, only 6/14 graders from Phase I were included in Phase II grading; however the distribution of grader levels was similar. Third, though the proportion of patients excluded was small, we are unable to fully characterise the reasons for these exclusions, due to the nature of the study as a programmatic evaluation. Some potential reasons for this are a patient's unwillingness to participate in the study, graders having forgotten to ask for patient consent to participate in the study, and patient inability to comply with image capture. Fourth, pupil status (size and cataract status) was not recorded in this study and this can be important for LMICs. Finally, it was not practical for the UK reference standard to examine patients clinically in Vietnam; however, the method of grading by a certified DR grader or clinical specialist is widely used as the reference standard in many screening programmes.

**Conclusions:** This paper shows how grading accuracy was particularly low among all grader groups in Vietnam in the first six months of DESP implementation. Many factors may have contributed to poor grader performance, including inadequate training and feedback, insufficient time to participate in quality assurance testing and competing work responsibilities. After additional training, testing and quality assurance systems were implemented in Vietnam, DTA improved among all grader groups, however a significant amount of work is still needed. In particular, training graders to detect ungradable cases is



For peer review only - http://bmjopen.bmj.com/site/about/guidelines.xhtml

crucial. With continuous quality improvement, monthly international test and training, periodic DR workshops and reviewal of certification, we would expect the DR sensitivity and specificity to improve. A qualitative study to determine why the initial training intervention was less successful should be explored. Since further improvements are required, understanding how other countries implement such programmes would be beneficial. Future studies should outline what DR training interventions were used, state relevant training courses and explain what quality assurance measures are in place. The findings from this study are important for DESP programme planners in Vietnam and other LMICs, highlighting the importance of quality monitoring and directed re-training as needed.

**Acknowledgements:** The authors are very grateful to all participants in Vietnam and Northern Ireland who generously gave their time for this study and to Orbis International.

**Author contributions:** All authors (KC, NC, TTH, LL, VTN, HTN, QHN, CD, GV, PP, HT, RS, MQT, TP) have made substantial contributions to the conception of the design of the work. KC and GV analysed and interpreted the data. All authors (KC, NC, TTH, LL, VTN, HTN, QHN, CD, GV, PP, HT, RS, MQT, TP) read and approved the final manuscript.

**Funding:** The project was funded by the Department for the Economy (DfE) – Global Challenges Research Fund (GCRF) Awards (Grant number: DFEGCRF17-18/Peto). None of the funders were involved in the design or conduct of the study; preparation, review or approval of the manuscript; or decision to submit the manuscript for publication. Nathan Congdon is supported by the Ulverscroft Foundation (UK) (No award/grant number). Katie Curran is funded by the Wellcome Trust (No award/grant number).

**Competing Interests:** Nathan Congdon is employed as a Research Director by Orbis International.

For peer review only

**Patient consent for publication:** Not required.

**Ethics approval:** This research adhered to the tenets of the Declaration of Helsinki. Ethical approval was granted by the Hanoi Medical University Institutional Review Board in Bio-Medical Research, Vietnam (No. 0518/HMU IRB). Written informed consent was obtained from all participants prior to their being interviewed.

**Data sharing statement:** All datasets relevant to the study are included in the article or uploaded as supplementary information.

For peer review only

## References

1. Ngoc NB, Lin ZL, Ahmed W. Diabetes: What challenges lie ahead for Vietnam? *Ann Glob Heal.* 2020;86(1):1–9.

2. Liew G, Michaelides M, Bunce C. A comparison of the causes of blindness certifications in England and Wales in working age adults (16-64 years), 1999-2000 with 2009-2010. *BMJ* Open. 2014;4(2):1–6.

3. Burgess PI, Msukwa G, Beare NAV. Diabetic retinopathy in sub-Saharan Africa: Meeting the challenges of an emerging epidemic. *BMC Med.* 2013;11(1):1.

4. Adriono G, Wang D, Octavianus C, Congdon N. Use of eye care services among diabetic patients in urban Indonesia. *Arch Ophthalmol* (Chicago, Ill 1960). 2011;129(7):930–5.

5. International Council of Ophthalmology. Number of Ophthalmologists in Practice and Training Worldwide [Internet]. 2012 [cited 2020 Aug 13]. Available from: http://www.icoph.org/ophthalmologists-worldwide.html

6. Gloucestershire Retinal Education Group. Diabetic Retinopathy Grading Course



For peer review only - http://bmjopen.bmj.com/site/about/guidelines.xhtml

[Internet]. 2020. [cited 2021 April] Available from: https://drscreening.org/diabetic-retinopathy-grading-course

7. Widdowson S, Taylor D. The management of grading quality: Good practice in the quality assurance of grading. [Internet] 2015. [cited 2021 April] Available from: https://assets.publishing.service.gov.uk/government/uploads/system/uploads/attachment_data/file/512832/The_Management_of_Grading.pdf

8. Public Health England. NHS Diabetic Eye Screening Programme Grading definitions for referable disease. [Internet] 2021 (cited 2021 September] Available from: https://www.gov.uk/government/publications/diabetic-eye-screening-retinal-image-grading-criteria/nhs-diabetic-eye-screening-programme-grading-definitions-for-referable-disease

9. NHS DESP Core Team. Diabetic Eye Screening Programme Pathway for Adequate / Inadequate Images and where images cannot be taken Outlines pathway and business rules for image capture exceptions and ungradable images. [Internet] 2013.[cited 2021 April] Available from: https://www.gov.uk/government/publications/diabetic-eye-screening-pathway-for-images-and-where-images-cannot-be-taken

10. Curran K, Congdon N, Peto T, Dardis C, Nguyen NN, Hoange TT et al. Capturing the clinical decision-making processes of expert and novice diabetic retinal graders using a 'think-aloud' approach. *Eye.* 2022;36:1019-1026

11. Murthy GVS, John N, Shamanna BR, Pant HB. Elimination of avoidable blindness due to cataract: Where do we prioritize and how should we monitor this decade. *Indian J Ophthalmol.* 2012;60(5):438–45.

12. McKenna M, Chen T, McAneney H, Membrillo MAV, Jin L, Xiao W, et al. Accuracy of trained rural ophthalmologists versus non-medical image graders in the diagnosis of



For peer review only - http://bmjopen.bmj.com/site/about/guidelines.xhtml

diabetic retinopathy in rural China. *Br J Ophthalmol*. 2018;102(11):1471–6.

13. Amirul Islam FM. Accuracy and reliability of retinal photo grading for diabetic retinopathy: Remote graders from a developing country and standard retinal photo grader in Australia. *PLoS One.* 2017;12(6):1–12.

14. Bhargava M, Cheung CYL, Sabanayagam C, Kawasaki R, Harper CA, Lamoureux EL, et al. Accuracy of diabetic retinopathy screening by trained non-physician graders using non-mydriatic fundus camera. *Singapore Med J*. 2012;53(11):715–9.

15. Rani PK, Peguda HK, Chandrashekher M, Swarna S, Jonnadula GB, James J et al. Capacity building for diabetic retinopathy screening by optometrists in India: Model description and pilot results. Indian J Ophthalmol 2021.69:655-659.

16. Srinivasan S, Shetty S, Natarajan V, Sharma T, Raman R. Development and Validation of a Diabetic Retinopathy Referral Algorithm Based on Single-Field Fundus Photography. PLoS One 2016.11:e0163108.

Figure 1: Flow diagram to illustrate enrolment of patients and management of images in Phase I from August to October 2018 (Initial grading performance analysis). Level 1 and level 2 graders graded the same set of photographs and level 3 graders graded a subset of these photographs: All disagreements between Level 1 and 2 graders and a 40% random sample of all images.

Figure 2: Flow diagram illustrating the enrolment of patients and management of images included in Phase II from January 2020 to October 2020 (Follow-up grading performance analysis after re-training). Level 1 and level 2 graders graded the same set of photographs and level 3 graders graded a subset of these images.

For peer review only

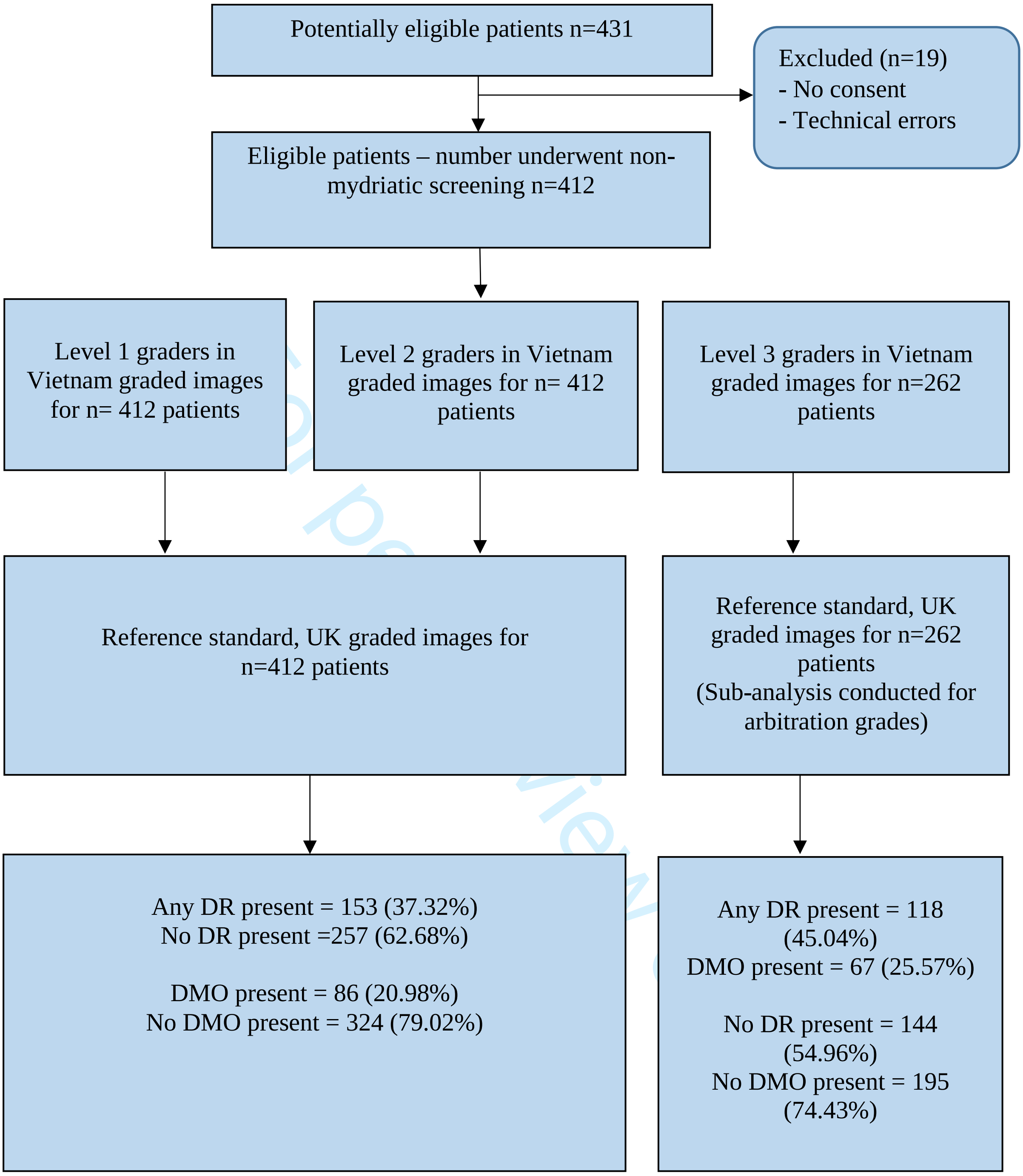


Figure 1: Flow diagram to illustrate enrolment of patients and management of images in Phase I from August to October 2018 (Initial grading performance analysis). Level 1 and level 2 graders graded the same set of photographs and level 3 graders graded a subset of these photographs: All disagreements between Level 1 and 2 graders and a 40% random sample of all images.

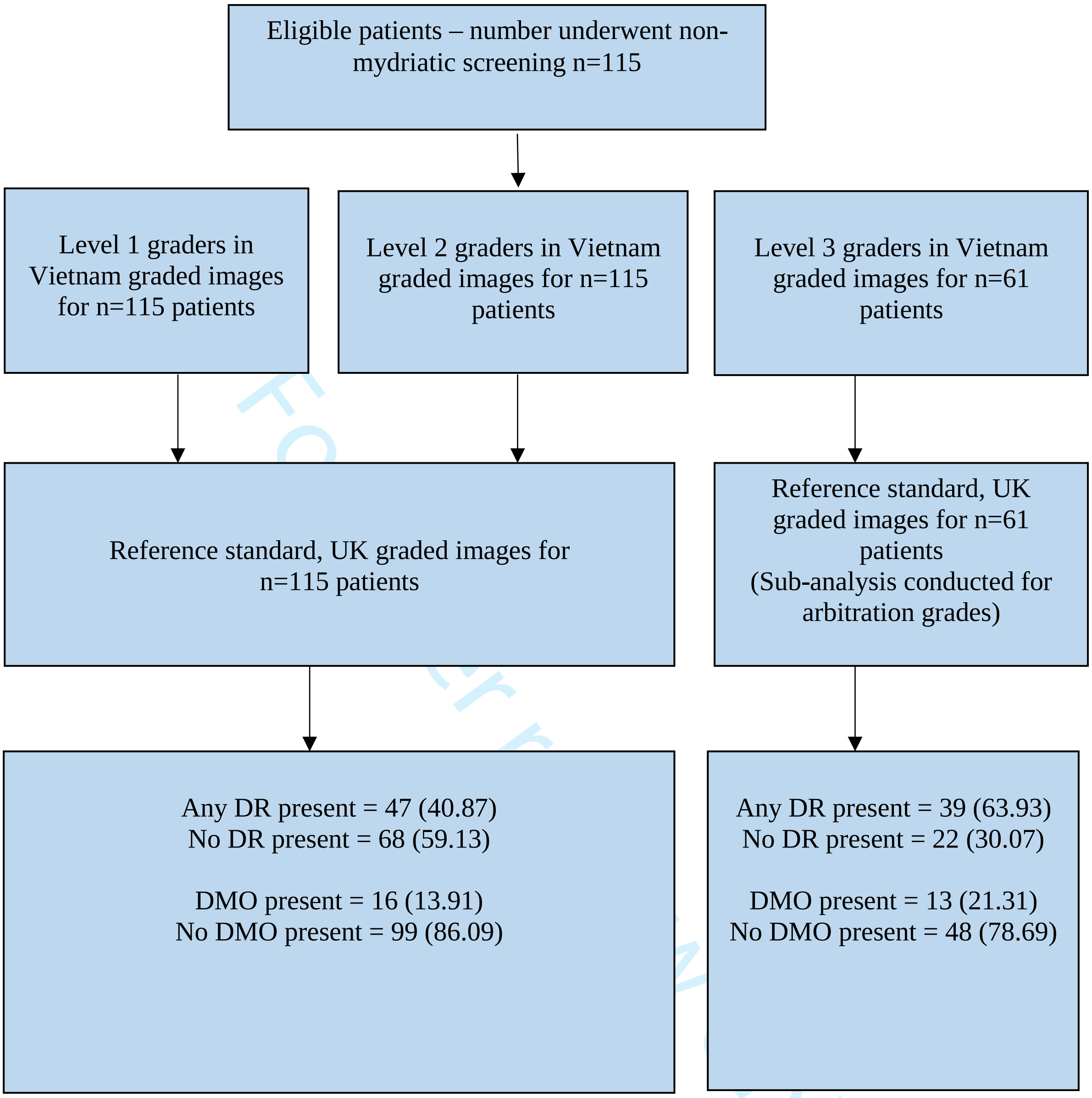


Figure 2: Flow diagram illustrating the enrolment of patients and management of images included in Phase II from January 2020 to October 2020 (Follow-up grading performance analysis after re-training). Level 1 and level 2 graders graded the same set of photographs and level 3 graders graded a subset of these images.

# SUPPLEMENTARY MATERIAL

**September 2017 (5 days)**

- Five Vietnamese doctors and medical administrators visited Northern Ireland to receive screener/graders training in administration and failsafe methods.
- These doctors delivered training to new graders in Vietnam following their visit to Northern Ireland.

**January 2018 (1 week) – Delivered in person in Vietnam by two UK graders (senior ophthalmic nurse and optometrist, certified in DR grading)**

- Observation in retina clinics.
- Hands on training with tabletop CR-2 Canon Fundus Cameras.
- Topics covered: ocular anatomy, retinal disease, DR signs, DR grading (based on the UK DESP grading classification system) and appropriate referral pathways and management (PowerPoint presentation and interactive sessions).
- All graders received a module workbook.
- Certifcation provided by Orbis.

**March 2018 - Grading began in Vietnam**

- Graders began grading as part of pilot DESP.
- Ongoing training was delivered by the Orbis team and the lead ophthalmologist for DR screening in Vietnam over the course of the following months.

**June 2018 (1-2 days) – Delivered by Orbis partners**

- UK graders developed a PowerPoint presentation based on DR case examples and this was delivered by Orbis.

**March 2019 (2 days) - Delivered by UK grader in Vietnam**

- More training on DR case examples.

**November 2019 (3 days) - Delivered in person in Vietnam by two UK graders (senior ophthalmic nurse and optometrist, certified in DR grading)**

- Refresher DR training, incorporating think-aloud techniques into practical teaching sessions.
- Pre and post training assessments.
- Encouraged use of international test and training (iTAT) for quality assurance purposes. Practical sessions on iTAT.

*Figure S1: A flow chart to highlight the training timeframe for graders in Vietnam*

For peer review only

**Diabetic retinopathy workshop for graders in Vietnam**

| | | |
|---|---|---|
| **AGENDA**<br><br>DAY 1-3 **Monday 29th January – Wednesday 31st January** | | |
| Visit diabetic retinopathy screening sites to observe and provide hands on training and support. | | UK graders and Orbis team |
| DAY 4- **Thursday 1st February** | | |
| **MORNING** | | |
| **9:00 - 9:30** | Check in | UK graders and Orbis team |
| **9:30 – 10.30** | Introduction on Diabetic Retinopathy | |
| **10.30-10.45** | Tea Break | |
| **10.45-11.30** | Basic Screening Component | |
| | Lunch | |
| **AFTERNOON** | | |
| **13.30-14:00** | NHS Grading System | UK graders and Orbis team |
| **14:00-14:45** | Image Quality | |
| **14:45-15:00** | Tea Break | |
| **15:00-16:00** | Image Grading | |
| **16:0-16:30** | Hospital pathway | |
| DAY 5- **Friday 2nd February** | | |
| **MORNING** | | |
| **9:00-9:30** | Other ocular findings | UK graders and Orbis team |
| **9:30-11:30** | Practice on grading | |
| **11:30-12:00** | Wrap up | |

For peer review only

**Refresher training programme for DR graders in Vietnam (delivered by UK graders and Orbis) in November 2019**

| Time | Topics | Method | Who | Preparation |
|---|---|---|---|---|
| **DAY 1** | | | | |
| **8:45-9:15** | Introduction/ Pre course quiz | | Orbis VN/ UK graders | UK graders |
| **9:15-9:45** | **Diabetic Retinopathy (DR) - New Challenges of Blindness Prevention** *Objective: Understand the problem of DR and current efforts to manage vision loss. Aim to motivate graders to be involved in DESP* | Presentation | Orbis VN | Orbis |
| **9:45-10:15** | **Retina Anatomy** *Objective : Understand the pathobiology of diabetic complications and pathogenesis of retinal damage* | Presentation | Ho Chi Minh Eye Hospital | Orbis |
| **10:30-11:15** | **Diabetic Retinopathy (DR) Pathophysiology** *Objective : Understand Diabetic Retinopathy* | Presentation | UK graders | UK graders |
| **11:15-12:00** | **Grading system and DR Grading pathway ( UK system)/ How to systematically grade a retinal image** *Objective : Understanding the grading system and referral pathway ( UK standard)* | Presentation | UK graders | UK graders |
| **13:30-14:15** | **Image quality** *Objective : Understand the requirements/criteria of image quality for accurate grading* | Presentation | UK graders | UK graders |
| **14:15-14:45** | **Spectra Software** *Objective : How to use the current Spectra software for uploading, grading, and managing DR cases* | Demonstration | Senior graders of Tien Giang and Ho Chi Minh Eye Hospital | |

| | | | | |
|---|---|---|---|---|
| **15:00-17:00** | **<u>Practical Training</u>**<br>**Parallel session:**<br>**1- Taking retina images of the patients following the procedure ,and provide counseling to the patients**<br><br>*Objective: Practice experience of taking fundus images* | Practical training | Current graders of Tien Giang and Ho Chi Minh Eye Hospital, and UK graders | 2 fundus cameras:<br>4 Groups: make sure every participant is able to practice at least once |
| **15:00-17:00** | **Parallel session :**<br>**1- Grading DR in the Spectra**<br><br>*Objective: Practical experience of how to do DR grading* | Practical training | Current graders of Tien Giang and Ho Chi Minh Eye Hospital, and UK graders | 3-4 accounts of Spectra<br>4 Groups : make sure every participant is able to practice at least once |
| | | **DAY 2** | | |
| **08:45-09:00** | *Recap of day 1/ introduction to day 2* | | Orbis | |
| **9:00-9:30** | **Analysis of retinal fundus images for grading of diabetic retinopathy severity**<br>Objective : How to read the image and protocol for retinal image analysis | | Ophthalmologist Ho Chi Minh Eye Hospital | |
| **9:30-10:15** | **DR Screening Procedure: Best Practice**<br>*Objective: Discuss how to build the "best screening procedures" into DESPs.* | Presentation | UK graders | UK graders |
| **10:30-11:15** | **Other Ocular Findings**<br><br>*Objective: Awareness of other ocular pathology during DR screening* | Presentation | UK graders | UK graders |
| **11:15-12:15** | **Image grading case studies competition** | Practical | UK graders | We need to organise people into groups of 3 with one experienced grader in each group |

| | | | | |
|---|---|---|---|---|
| **13:30-14:30** | **Counselling and delivering messages to patients during the DR screening**<br>*Objective: Important to provide counselling for the patients and deliver messages effectively.* | Presentation/ practical training | Orbis Vietnam | |
| **14:30-16:30** | **<u>Practical Training</u>**<br>**Parallel session:**<br>**1- Taking retina images of the patients following the procedure ,and provide counseling to the patients**<br><br>*Objective: Experience on how to take good fundus photographs* | Practical training | Current graders of Tien Giang and Ho Chi Minh Eye Hospital, and UK graders | 2 fundus camera<br>4 Group: make sure every participants are able to practice at least one time |
| | **Parallel session :**<br>**1- Grading DR in the Spectra**<br><br>*Objective: Practical experience on DR grading* | Practical training | Current graders of Tien Giang and Ho Chi Minh Eye Hospital, and UK graders | -4 accounts of Spectra<br>4 Groups: make sure every participant is able to practice at least one time. |
| **DAY 3**<br>**Part 1 (Final practical training)** | | | | |
| **08:45-9:00** | Recap of day 2/ introduction to day 3 | | | |
| **09:00-09:30** | **Quality Assurance in Diabetic Screening**<br>*Objective : Understand why quality assurance is important and the correct steps required to ensure good quality assurance procedures are in place* | Presentation | UK graders | UK graders |
| **09:30-10:30** | **Practice : Grading in iTAT**<br>*Objective: Know the Online training for DR grading and the importance of lifelong learning for DR grading* | Practice | Current graders of Tien Giang and Ho Chi Minh Eye Hospital, and support from UK graders | ITAT accounts for practicing |

| | | | | |
|---|---|---|---|---|
| **10:45-12:30** | **<u>Assessment</u>**<br><br>**Parallel session:**<br>**1- Taking retina images of the patients following the procedure ,and provide counseling to the patients**<br>**2- Grading DR in the Spectra** | Practical Training | Current graders of Tien Giang and Ho Chi Minh Eye Hospital, and UK graders | 2 fundus cameras<br>4 Group and 4 accounts of Spectra<br>4 Groups |
| **DAY 3**<br>**Part 2 (Future planning)** | | | | |
| **13:30-14:15** | **Post course Quiz and results** | | UK graders | UK graders |
| **14:15-15:00** | **Teaching Methodology for adults**<br>*Objective : How to train new graders effectively* | Think aloud work shop | Orbis VN Supported by UK graders | UK graders |
| **15:15-16:00** | **Supportive Supervision Methodology: Developing quality improvement**<br>*Objective: How to plan, implement the supervision trips to correct / improve other graders' performances. Provide checklist tools* | Think aloud work shop | Orbis VN Supported by UK graders | UK graders |
| **16:00-16:45** | **Feedbacks and Plan for next steps** | Discussion | | |
| **16:45-17:00** | **Certificates for Vietnamese graders in attendance** | | | |

For peer review only

**Table S1: UK DR Grading Classification Scale**

| NSC | International Term | Symptoms | Features | Action |
|---|---|---|---|---|
| **R0** | No DR | None | No signs of diabetic retinopathy | Annual rescreen |
| **RI** | Mild none-proliferative (mild pre-proliferative) | None | Haemorrhages & microaneurysms, only | Annual rescreen |
| **R2** | Moderate none-proliferative, moderate pre-proliferative | None | Extensive Microaneurysms, intraretinal haemorrhages, hard exudates, venous abnormalities, large blot haemorrhages, cotton wool spots (small infarcts), venous beading, venous loop, venous reduplication. | Refer routinely to HES |
| **R3s** | Stable proliferative diabetic retinopathy | | No haemorrhages or exudates or new vessels, laser scars | Annual rescreen |
| **R3a** | Active proliferative diabetic retinopathy | Floaters, central loss of vision | New vessel formation either at the disc (NVD) or elsewhere (NVE). Extensive fibrovascular proliferation, retinal detachment, pre-retinal or vitreous haemorrhage. | Urgent referral to HES |
| **M 0** | | | No maculopathy | Annual rescreen |
| **M 1** | Diabetic maculopathy | Blurred central vision | The macula is defined as a circle centred on the fovea, with a radius of the distance to the disc margin.<br>If the leakage involves or is near the fovea the condition is termed clinically significant macular oedema (CSME).<br><br>Exudative maculopathy presents with leakage, retinal thickening, microaneurysms, hard exudates at the macula. Ischaemic form can have a featureless macular with NVE and poor vision.<br>Milder forms:<br>• exudate < or = 1DD of centre of fovea<br>• circinate or group of exudates within macula<br>• any microaneurysm or haemorrhage < or = 1DD of centre of fovea only is associated with a best VA of < or = 6/12 retinal thickening < or = | Refer to HES |

| | | | | |
|---|---|---|---|---|
| | | | 1DD of centre of fovea (if stereos available) | |
| **P** | Photocoagulation | Reduced night vision, glare | Small retinal scars throughout the peripheral retina. | |
| **U** | Ungradable | | Ungradable is usually due to cataract, small pupils, other lesions usually referred for assessment | Refer for slit lamp examination |
| **Abbreviations:** DR = diabetic retinopathy, NPDR = none-proliferative retinopathy, NVE = new vessels elsewhere, IRMAs = intraretinal microvascular abnormalities (part of severe pre-proliferative retinopathy, vessels will not leak with angiogram, otherwise they would be 'new vessels' making the condition 'proliferative'), MO=macular oedema, MA= microaneurysm, DD=disc diameter, HES= hospital eye service | | | | |

For peer review only

**Table S2: Reference standards intra-rater agreement score using kappa statistic (first attempt versus second attempt)**

| | Intra-rater agreement (reference standard, UK), k (95% CI) (by eyes, n=106) | Intra-rater agreement (reference standard, UK), k (95% CI) (by worst eye, n=53) |
|---|---|---|
| **Overall Diabetic Retinopathy Grading:** | | |
| **Any DR** | 0.96 (0.91,1.00) | 0.92 (0.82, 1.00) |
| **Treatable DR** | 0.81 (0.60, 1.00) | 0.74 (0.47, 1.00) |
| **Referable Maculopathy** | 0.97 (0.92, 1.0)0 | 1.00 (1.00, 1.00) |
| Abbreviations: CI=confidence interval, k=kappa, DR=Diabetic retinopathy, DMO=diabetic macular oedema<br>Any DR defined as R1, R2, R3s, R3a and U<br>Treatable DR defined as R3a<br>Referable DMO defined as M1 and U | | |

**Table S3: Using kappa statistic to determine the inter-rater agreement between the reference standard and one senior grader from QUB grading centre**

| | Inter-rater agreement (reference standard vs a senior grader QUB), k (95% CI) (by eyes, n=106) | Inter-rater agreement (reference standard vs a senior grader QUB (by worst), k (95% CI) (by worst eye, n=53) |
|---|---|---|
| **Overall Diabetic Retinopathy Grading:** | | |
| **Any DR** | 0.79 (0.67, 0.91) | 0.74 (0.55, 0.92) |
| **Treatable DR** | 0.71 (0.48, 0.95) | 0.68 (0.39, 0.97) |
| **Referable Maculopathy** | 0.75 (0.61, 0.90) | 0.74 (0.55, 0.93) |
| Abbreviations: CI=confidence interval, k=kappa, DR=Diabetic retinopathy, DMO=diabetic macular oedema<br>Any DR defined as R1, R2, R3s, R3a and U<br>Treatable DR defined as R3a<br>Referable DMO defined as M1 and U | | |

**Intra and inter-grader agreement**

To ensure there was good intra-grader reliability as a reference standard, a stratified random sample of images were regraded. There was approximately one month between the first and second attempts to reduce the possibility of bias caused by memory. Additionally, inter-grader agreement was calculated using kappa to ensure there was good grading agreement between the reference standard and one senior grader from the Ophthalmic Reading Centre at QUB, Belfast. Any disagreements were discussed with

For peer review only

a retinal specialist until consensus was reached. Overall, the intra-grader agreement and inter-grader agreement ranged from substantial to almost perfect.

**Table S4: The prevalence of any diabetic retinopathy (DR), referable DR, any maculopathy and ungradable cases with the reference grader from Phase I and Phase II**

| Diabetic Retinopathy grades | Phase I | Phase II (post remedial training) | P-Value |
|---|---|---|---|
| R0 (n,%) | 257 (62.68) | 68 (59.13) | P=0.347 |
| R1 (n,%) | 100 (24.39) | 32 (27.83) | |
| R2 (n,%) | 11 (2.68) | 2 (1.74) | |
| R3a (n,%) | 10 (2.44) | 7 (6.09) | |
| R3s (n,%) | 1 (0.24) | 0 (0.00) | |
| U (n,%) | 31 (7.56) | 6 (5.22) | |
| **Any DR** | | | |
| - Yes (n,%) | 153 (37.32) | 47 (40.87) | P=0.488 |
| - No (n,%) | 257 (62.68) | 68(59.12) | |
| **Referable DR** | | | |
| - Yes (n,%) | 52 (12.68) | 15 (13.04) | P=0.918 |
| - No (n,%) | 358 (87.32) | 100 (86.96) | |
| **Any DMO** | | | |
| - M0 (n,%) | 324 (79.02) | 99 (86.09) | |
| - M1 (n,%) | 43 (10.49) | 10 (8.70) | P=0.173 |
| - U (n,%) | 43 (10.49) | 6 (5.22) | |
| **Abbreviations:** DR=diabetic retinopathy, DMO=Diabetic Macular Oedema, U=ungradable<br>Chi-Squares used to test significance. | | | |

For peer review only

**Table S5. Diagnostic test accuracy of DR graders in Vietnam against a reference standard from the UK, excluding ungradable images.**

| | Level 1 graders (n=373 patient images) | Level 2 graders (n=373 patient images)* | Level 3 graders (n=235 patient images )† |
|---|---|---|---|
| **Any DR** | | | |
| Sensitivity (%) (95% CI) | 47.9 (38.8, 57.2) | 50.8 (41.6, 60.0) | 49.0 (38.7, 59.3) |
| Specificity (%) (95% CI) | 89.7 (85.1, 93.0) | 98.8 (96.3, 99.7) | 100 (96.8, 100) |
| PPV (%) (95% CI) | 69.0 (57.9, 78.4) | 95.3 (86.2, 98.8) | 100 (90.6, 100) |
| NPV (%) (95% CI) | 78.2 (72.9, 82.7) | 80.9 (76.0, 85.0) | 74.6 (67.8, 80.5) |
| **Referable DR** | | | |
| Sensitivity (%) (95% CI) | 38.1 (19.0, 61.3) | 28.6 (12.2, 52.3) | 22.2 (7.4, 48.1) |
| Specificity (%) (95% CI) | 98.9 (96.9, 99.6) | 100 (98.7, 100) | 99.5 (97.1, 99.9) |
| PPV (%) (95% CI) | 66.7 (35.4, 88.7) | 100 (51.7, 100) | 80.0 (29.9, 98.9) |
| NPV (%) (95% CI) | 96.4 (93.8, 97.9) | 96.0 (93.3, 97.6) | 94.0 (89.9, 96.6) |
| **Referable DMO** | | | |
| Sensitivity (%) (95% CI) | 9.3 (3.0, 23.1) | 37.2 (23.4, 53.3) | 26.5 (13.5, 44.7) |
| Specificity (%) (95% CI) | 99.1 (97.0, 99.8) | 99.4 (97.5, 99.9) | 100 (97.6, 100) |
| PPV (%) (95% CI) | 57.1 (20.2, 88.2) | 88.9 (63.9, 98.1) | 100 (62.9, 100) |
| NPV (%) (95% CI) | 88.9 (85.1, 92.0) | 92.3 (88.9, 94.7) | 88.6 (83.5, 92.4) |

**Abbreviations:** UK = United Kingdom, DR = Diabetic Retinopathy, DMO = Diabetic Macular Oedema, CI = Confidence Intervals,
Grading criteria: UK National Diabetic Eye Screening Programme (NDESP) classification system (See supplementary material, Table S1 for more details).
Any DR, is defined as grades R1, R2, R3s and R3a.
Referable DR is defined as grades R2 and R3a.
Referable DMO is defined as grades M1
Sensitivity is the ability of a test to correctly identify patients with a disease and specificity is the ability of a test to correctly identify people without the disease
Positive predictive value (PPV) is the proportion of those who test positive who have the condition (true positives) and negative predictive value (NPV) is the proportion of those who test negative who do not have the condition (true negatives).

For peer review only

**Table S6: Diagnostic test accuracy of DR graders in Vietnam against a reference standard from the UK after additional DR training was delivered, excluding ungradable images**

| | Level 1 graders (n=115 patient images) | Level 2 graders (n=115 patient images) | Level 3 graders (n=62 patient images) |
|---|---|---|---|
| **Any DR** | | | |
| Sensitivity (%) (95% CI) | 97.6 (85.6, 99.9) | 72.5 (55.9 84.9) | 55.6 (38.1, 72.1) |
| Specificity (%) (95% CI) | 95.6 (86.8, 99.8) | 100 (93.5, 100) | 100 (84.6, 100) |
| PPV (%) (95% CI) | 93.0 (79.9, 98.2) | 100 (85.4, 100) | 100 (80.0, 100) |
| NPV (%) (95% CI) | 98.5 (90.7, 99.9) | 85.5 (75.2, 92.2) | 57.9 (10.9, 73.2) |
| **Referable DR** | | | |
| Sensitivity (%) (95% CI) | 88.9 (50.7, 99.4) | 55.6 (22.7, 84.7) | 77.8 (40.0, 97.2) |
| Specificity (%) (95% CI) | 90.0 (81.9, 94.8) | 96.9 (90.5, 99.2) | 100 (92.8, 100) |
| PPV (%) (95% CI) | 44.4 (22.4, 68.7) | 62.5 (25.9, 89.8) | 100 (56.1, 100) |
| NPV (%) (95% CI) | 98.9 (93.4, 99.9) | 95.9 (89.2, 98.7) | 96.1 (87.8, 98.8) |
| **Referable DMO** | | | |
| Sensitivity (%) (95% CI) | 90.0 (54.1, 99.5) | 60.0 (26.4, 86.3) | 80.0 (44.4, 97.5) |
| Specificity (%) (95% CI) | 97.0 (91.8, 99.2) | 97.9 (91.9, 99.6) | 100 (92.6, 100) |
| PPV (%) (95% CI) | 75.0 (42.8, 93.3) | 75.0 (35.6, 95.5) | 100 (59.8, 100) |
| NPV (%) (95% CI) | 99.0 (93.6, 99.9) | 95.9 (89.2, 98.7) | 96.0 (87.4, 99.6) |

**Abbreviations:** UK = United Kingdom, DR = Diabetic Retinopathy, DMO = Diabetic Macular Oedema, CI = Confidence Intervals,
Grading criteria: UK National Diabetic Eye Screening Programme (NDESP) classification system (See supplementary material, Table S1 for more details).
Criteria:
Any DR is defined as grades R1, R2, R3s, and R3a.
Referable DR is defined as grades R2 and R3a.
Referable DMO is defined as grades M1.
Sensitivity is the ability of a test to correctly identify patients with a disease and specificity is the ability of a test to correctly identify people without the disease
Positive predictive value (PPV) is the proportion of those who test positive who have the condition (true positives) and negative predictive value (NPV) is the proportion of those who test negative who do not have the condition (true negatives).

For peer review only

| Section & Topic | No | Item | Reported on page # |
|---|---|---|---|
| **TITLE OR ABSTRACT** | | | |
| | **1** | Identification as a study of diagnostic accuracy using at least one measure of accuracy (such as sensitivity, specificity, predictive values, or AUC) | 1 |
| **ABSTRACT** | | | |
| | **2** | Structured summary of study design, methods, results, and conclusions (for specific guidance, see STARD for Abstracts) | 3-4 |
| **INTRODUCTION** | | | |
| | **3** | Scientific and clinical background, including the intended use and clinical role of the index test | 5-6 |
| | **4** | Study objectives and hypotheses | 5 |
| **METHODS** | | | |
| *Study design* | **5** | Whether data collection was planned before the index test and reference standard were performed (prospective study) or after (retrospective study) | 5 |
| *Participants* | **6** | Eligibility criteria | 6-7 |
| | **7** | On what basis potentially eligible participants were identified (such as symptoms, results from previous tests, inclusion in registry) | 6-7 |
| | **8** | Where and when potentially eligible participants were identified (setting, location and dates) | 6-7 |
| | **9** | Whether participants formed a consecutive, random or convenience series | 7 |
| *Test methods* | **10a** | Index test, in sufficient detail to allow replication | 6-7 |
| | **10b** | Reference standard, in sufficient detail to allow replication | 6 |
| | **11** | Rationale for choosing the reference standard (if alternatives exist) | 6 |
| | **12a** | Definition of and rationale for test positivity cut-offs or result categories of the index test, distinguishing pre-specified from exploratory | |
| | **12b** | Definition of and rationale for test positivity cut-offs or result categories of the reference standard, distinguishing pre-specified from exploratory | |
| | **13a** | Whether clinical information and reference standard results were available to the performers/readers of the index test | 6-7 |
| | **13b** | Whether clinical information and index test results were available to the assessors of the reference standard | 6-7 |
| *Analysis* | **14** | Methods for estimating or comparing measures of diagnostic accuracy | 12-14 |
| | **15** | How indeterminate index test or reference standard results were handled | 12-14 Supplementary file |
| | **16** | How missing data on the index test and reference standard were handled | 12-14 |
| | **17** | Any analyses of variability in diagnostic accuracy, distinguishing pre-specified from exploratory | 12-14 |
| | **18** | Intended sample size and how it was determined | NA |
| **RESULTS** | | | |
| *Participants* | **19** | Flow of participants, using a diagram | 10-11 |
| | **20** | Baseline demographic and clinical characteristics of participants | 9 |
| | **21a** | Distribution of severity of disease in those with the target condition | Supplementary file |
| | **21b** | Distribution of alternative diagnoses in those without the target condition | Supplementary file |
| | **22** | Time interval and any clinical interventions between index test and reference standard | Supplementary file |
| *Test results* | **23** | Cross tabulation of the index test results (or their distribution) by the results of the reference standard | 12-14 and supplementary |
| | **24** | Estimates of diagnostic accuracy and their precision (such as 95% confidence intervals) | 12-14 |
| | **25** | Any adverse events from performing the index test or the reference standard | NA |
| **DISCUSSION** | | | |
| | **26** | Study limitations, including sources of potential bias, statistical uncertainty, and generalisability | 17 |
| | **27** | Implications for practice, including the intended use and clinical role of the index test | 17-18 |
| **OTHER INFORMATION** | | | |
| | **28** | Registration number and name of registry | NA |
| | **29** | Where the full study protocol can be accessed | NA |

For peer review only - http://bmjopen.bmj.com/site/about/guidelines.xhtml

cc BY SA

| | | | |
|---|---|---|---|
| | 30 | Sources of funding and other support; role of funders | 18 |

For peer review only

# The impact of targeted diabetic retinopathy training for graders in Vietnam and the implications for future diabetic retinopathy screening programmes: a diagnostic test accuracy study

| | |
|---|---|
| Journal: | *BMJ Open* |
| Manuscript ID | bmjopen-2021-059205.R2 |
| Article Type: | Original research |
| Date Submitted by the Author: | 25-Jul-2022 |
| Complete List of Authors: | Curran, Katie; Queen's University Belfast School of Medicine Dentistry and Biomedical Sciences, Centre of Public Health<br>Congdon, Nathan; Queen’s University of Belfast; ORBIS International<br>Hoang, Tung; Hanoi Medical University, Department of Ophthalmology; The University of Sydney Save Sight Institute<br>Lohfeld, Lynne; Queen’s University of Belfast; Eye Hospital of Wenzhou Medical University<br>Nguyen, Van; Orbis International in Vietnam<br>Nguyen, Hue; Orbis International in Vietnam<br>Nguyen, Quan; Ho Chi Minh Eye Hospital, Vitreo-Retina Department<br>Dardis, Catherine; Belfast Health and Social Care Trust, Department of Ophthalmology<br>Virgili, Gianni ; Queen’s University of Belfast; Belfast Health and Social Care Trust, Department of Ophthalmology<br>Piyasena, Prabhath; Queen’s University of Belfast<br>Tran, Huong; Orbis International in Vietnam<br>Salongcay, Recivall; Queen's University Belfast<br>Tung, Mai; Ho Chi Minh Eye Hospital, Vitreo-Retina Department<br>Peto, Tunde; Queen’s University of Belfast; Belfast Health and Social Care Trust, Department of Ophthalmology |
| <b>Primary Subject Heading</b>: | Ophthalmology |
| Secondary Subject Heading: | Diabetes and endocrinology, Medical education and training |
| Keywords: | Diabetic retinopathy < DIABETES & ENDOCRINOLOGY, OPHTHALMOLOGY, PUBLIC HEALTH, Quality in health care < HEALTH SERVICES ADMINISTRATION & MANAGEMENT |
| | |

SCHOLARONE™ Manuscripts

For peer review only - http://bmjopen.bmj.com/site/about/guidelines.xhtml

*I, the Submitting Author has the right to grant and does grant on behalf of all authors of the Work (as defined in the below author licence), an exclusive licence and/or a non-exclusive licence for contributions from authors who are: i) UK Crown employees; ii) where BMJ has agreed a CC-BY licence shall apply, and/or iii) in accordance with the terms applicable for US Federal Government officers or employees acting as part of their official duties; on a worldwide, perpetual, irrevocable, royalty-free basis to BMJ Publishing Group Ltd ("BMJ") its licensees and where the relevant Journal is co-owned by BMJ to the co-owners of the Journal, to publish the Work in this journal and any other BMJ products and to exploit all rights, as set out in our licence.*

*The Submitting Author accepts and understands that any supply made under these terms is made by BMJ to the Submitting Author unless you are acting as an employee on behalf of your employer or a postgraduate student of an affiliated institution which is paying any applicable article publishing charge ("APC") for Open Access articles. Where the Submitting Author wishes to make the Work available on an Open Access basis (and intends to pay the relevant APC), the terms of reuse of such Open Access shall be governed by a Creative Commons licence – details of these licences and which Creative Commons licence will apply to this Work are set out in our licence referred to above.*

*Other than as permitted in any relevant BMJ Author's Self Archiving Policies, I confirm this Work has not been accepted for publication elsewhere, is not being considered for publication elsewhere and does not duplicate material already published. I confirm all authors consent to publication of this Work and authorise the granting of this licence.*

For peer review only

**Title: The impact of targeted diabetic retinopathy training for graders in Vietnam and the implications for future diabetic retinopathy screening programmes: a diagnostic test accuracy study**

**Short title:** Training diabetic retinopathy graders in Vietnam

**Authors:**

*Katie Curran, MPH, PhD[1] (k.curran@qub.ac.uk)

Nathan Congdon, MD, MPH[1,2,3] (Nathan.Congdon@orbis.org)

Tung Thanh Hoang[4,5] (tung.hoang@sydney.edu.au)

Lynne Lohfeld, MPH, PhD[1,6] (l.lohfeld@qub.ac.uk)

Van Thu Nguyen, BA[7] (van.nguyen@orbis.org)

Hue Thi Nguyen, MD, MPH[7] (hue.nguyen@orbis.org)

Quan Nhu Nguyen, MD, PhD[8] (bsquan2009@gmail.com)

Catherine Dardis, MSc[9] (cathy831oph@gmail.com)

Gianni Virgili MD[1,9] (G.Virgili@qub.ac.uk)

Prabhath Piyasena, MB BS, PhD[1], (m.piyasena@qub.ac.uk)

Huong Tran, MPA[7] (Huong.Tran@orbis.org)

Recivall Salongcay, MD, MPM[1] (R.SALONGCAY@qub.ac.uk)

Mai Quoc Tung, MD, PhD[8] (maitungtn@gmail.com)

For peer review only

Tunde Peto, MD, PhD[1,9] (T.Peto@qub.ac.uk)

**Affiliations:**

[1]Centre for Public Health, Queen's University of Belfast, Belfast, UK

[2]Orbis International, New York, USA

[3]Zhongshan Ophthalmic Centre, Guangzhou, China

[4]Ophthalmology Department, Hanoi Medical University, Hanoi, Vietnam

[5]Save Sight Institute, The University of Sydney School of Medicine, Sydney, Australia

[6]Eye Hospital of Wenzhou Medical University, Wenzhou, Zhejiang Province, China

[7]Orbis International in Vietnam, Hanoi, Vietnam

[8]Vitreo-Retina Department, Ho Chi Minh Eye Hospital, Ho Chi Minh City, Vietnam

[9]Department of Ophthalmology, Belfast Health and Social Care Trust, NI

***Corresponding author**

Katie Curran

Centre for Public Health

Institute of Clinical Sciences, A

Queen's University Belfast

BT12 6BA

Email: k.curran@qub.ac.uk

For peer review only

**Keywords:** Diabetic retinopathy, diabetic retinopathy training, screening

**Word Count:** 2785

**Abstract**

**Objectives:** To compare the accuracy of trained level 1 diabetic retinopathy (DR) graders (nurses, endocrinologists, one general practitioner), level 2 graders (mid-level ophthalmologists) and level 3 graders (senior ophthalmologists) in Vietnam against a reference standard from the UK, and assess the impact of supplementary targeted grader training.

**Design:** Diagnostic test accuracy study.

**Setting:** Secondary care hospitals in Southern Vietnam.

**Participants:** DR training was delivered to Vietnamese graders in February 2018 by National Health System (NHS) UK graders. Two-field retinal images (412 patient images) were graded by 14 trained graders in Vietnam between August-October 2018 and then re-graded retrospectively by an NHS-certified reference standard UK optometrist (Phase I). Further DR training based on Phase I results was delivered to graders in November 2019. After training, a randomised subset of images from January-October 2020 (115 patient images) was graded by six of the original cohort (Phase II). The reference grader re-graded all images from Phase I and II retrospectively in masked fashion.

**Primary and secondary outcome measures:** Sensitivity was calculated at the two different time points and Chi-Squared was used to test significance.



For peer review only - http://bmjopen.bmj.com/site/about/guidelines.xhtml

**Results:** In Phase I, the sensitivity for detecting any DR for all grader groups in Vietnam was low (41.8-42.2%) and improved in Phase II after additional training was delivered (51.2-87.3%). The greatest improvement was seen among level 1 graders ($P<0.001$) and the lowest improvement was observed among level 3 graders (P=0.326). There was a statistically-significant improvement in sensitivity for detecting referable DR and referable diabetic macular oedema between all grader levels. The post-training values ranged from 40.0-61.5% (including ungradable images) and 55.6%-90.0% (excluding ungradable images).

**Conclusions:** This study demonstrates that targeted training interventions can improve accuracy of DR grading. These findings have important implications for improving service delivery in DR screening programmes in low-resource settings.

**Article Summary**

**Strengths and limitations of this study**

- Graders in Vietnam were trained to detect DR based on the UK's DR screening model.
- This study describes the impact of a training intervention to improve DR grading in Vietnam
- Gradable and ungradable fundus image grading were included in the analysis.
- The sample size was smaller in Phase II compared to Phase I.

For peer review only

## Introduction

The prevalence of diabetes among adults in Vietnam is approximately 6% and has almost doubled over the past decade.[1] Early detection through diabetic eye screening programmes (DESPs) is important to reduce the risk of avoidable blindness due to diabetic retinopathy (DR). Since the introduction of systematic DESPs in the UK, a high-income country (HIC), diabetic retinopathy (DR) is no longer the leading cause of blindness among working age adults.[2] The key to such successful DESPs is implementing accurate, innovative and cost-effective models tailored to fit healthcare systems and contexts.

Investing in training personnel to increase human resources and procuring appropriate diagnostic and treatment equipment are essential to ensure that service providers can deliver optimum care for people with DR. In low-middle income countries (LMICs), there is often insufficient capacity to implement robust DESPs due to the lack of skilled human resources and infrastructure.[3,4] In Vietnam, there are only 14 ophthalmologists per million population compared to 49 per million in the UK.[5]

All screening programmes must provide evidence of their ability to detect the targeted condition and ensure that the service performs efficiently to improve screening accuracy when it falls short. To date, there is insufficient evidence on DR grading accuracy using non-mydriatic digital imaging by trained graders in LMICs, and even less about the capacity of DESPs in LMICs to improve where poor accuracy is detected. The current retrospective study is designed to assess accuracy of a range of graders in a non-governmental organisation (NGO)-supported DESP in Vietnam, and to study the efficacy of a quality-improvement intervention.

## Methods



For peer review only - http://bmjopen.bmj.com/site/about/guidelines.xhtml

**Study participants**: The 14 participants from Vietnam in Phase I included: Level 1 DR graders (6 nurses, 1 general practitioner and 2 endocrinologists, all with < 1 year grading experience, 55.6% female), Level 2 DR graders (3 newly-qualified ophthalmologists with < 1 year formal DR grading experience, 100% female), and Level 3 DR graders (2 senior ophthalmologists with >5 years' experience providing treatment for sight threatening DR, but with <1 year formal DR grading experience, 100% male). In Phase II, 6/14 graders (3 Level 1, 2 Level 2, 1 Level 3) from Phase I were included. The reference standard from the UK (KC) was a fully-qualified optometrist trained in DR grading and certified by the UK NHS DESP.[6] Vietnamese Level 1, 2 and 3 graders are equivalent to primary, secondary and arbitration graders, respectively, in UK DESPs.[7] In the current study, Vietnamese Level 1 and Level 2 graders graded all fundus images for DR. All images having disagreement between graders, and an additional randomly-selected 40% of all images, were sent for arbitration grading by Level 3 graders in Vietnam. All graders in Vietnam were masked to any prior diagnoses or grades of the reference standard, while the reference standard was also masked to results of grading in Vietnam. Fundus images were graded for 412 patients in phase I and 115 patients in phase II (Figure 1 and figure 2).

**DR training for graders in Vietnam:** As part of a DESP project supported by NGO Orbis International, a team of five Vietnamese doctors and medical administrators visited a Northern Ireland (NI) DESP in September 2017 to receive training on screening, programme administration and quality control methods. In February 2018, a senior UK NHS grader from the Belfast Trust (CD) and a fully-qualified optometrist, trained in DR grading and certified by the NHS (KC), visited Vietnam to deliver DR training to graders involved in the DESPs. (Supplementary, Figure S1 for training timeline). Training focused on ocular anatomy, retinal diseases, DR signs and grading (based on the UK National Screening Committee (NSC)

For peer review only



For peer review only - http://bmjopen.bmj.com/site/about/guidelines.xhtml

classification system), and appropriate referral pathways and management (Supplementary, Tables S1-S3).[8]

**Image acquisition and management:** Images were captured by trained nurses and technicians in Vietnam. Two-field, 45° digital colour photographs (one disc-centred and one macula-centred) were taken using a tabletop non-mydriatic fundus camera (Canon CR2-AF, Canon Medical Systems. Europe), in accordance with the UK's NHS DESP.[9] Nurses and technicians were trained to repeat inadequate images as a quality control measure and take anterior segment photographs where adequate fundus images were not possible. Images were anonymised and uploaded to a cloud-based software system (Spectra)® for analysis by trained DR graders in Vietnam. The images were transferred to a Queen's University Belfast (QUB) server for re-grading by the reference standard.

**Assessment of gradeability:** Image quality was defined as 'adequate' or 'inadequate' in accordance with NHS DESP guidelines as outlined below;

- Adequate disc-centred image: complete optic disc >2DD from edge of image and fine vessels visible on surface of the disc.[9]
- Adequate macula-centred image: centre of fovea >2DD from edge of image and vessels visible within 1DD of centre of fovea.[9]

The disc-centred and macula-centred images for each eye were viewed as a pair and graded at an individual eye level. The presence of DR and diabetic macular oedema (DMO) was also determined at a patient level and based on the worst affected eye. Participants with ungradable images were referred for further slit-lamp examination. Where images were considered inadequate but referable disease was detectable, the referable grade was recorded and the patients were moved onto the appropriate referable grade pathway.[9]



For peer review only - http://bmjopen.bmj.com/site/about/guidelines.xhtml

Consecutive patients diagnosed with diabetes and undergoing evaluation for possible DR at Ho Chi Minh City General Hospital and Ho Chi Minh Eye Hospital (tertiary hospitals), Tien Giang General Hospital (provincial hospital) and Cai Ba General Hospital (district hospital) in Vietnam were recruited. Fundus images from August to October 2018 (Phase I) were graded by 14 graders in Vietnam and then re-graded retrospectively by a reference standard from the UK in Phase I. Targeted remedial training, based on specific findings from the Phase I analysis, was delivered in March 2019 and November 2019 by UK graders and Orbis. (Supplementary material, figure S1) Additionally, regular testing and training for quality assurance purposes was also introduced, similar to UK DESP models. To evaluate the impact of this quality-improvement intervention, a new subset of images was graded by six of the original cohort of graders between January-October 2020 (Phase II) and re-graded by the reference standard from the UK (KC) in September 2021.

**Statistical analysis:** Data were entered into Microsoft Excel version 16.0 and then transferred to Stata 17.0 (StataCorp LLC) for analysis. Intra and inter-grader agreement was calculated using kappa and a stratified random sampling technique was utilised to ensure a representative sample of images was re-graded (Supplementary, Tables S4 and S5). Diagnostic test accuracy (DTA) comparing graders in Vietnam with the UK reference standard was assessed by calculating sensitivity, specificity, positive predicative values (PPV) and negative predictive values (NPV). Sensitivity was calculated at the two different time points (Phase I and Phase II) and Chi Squared was used to test significance.

**Patient and Public Involvement:** Patients or the public were not involved in the design, or conduct, or reporting, or dissemination plans of our research

For peer review only



For peer review only - http://bmjopen.bmj.com/site/about/guidelines.xhtml

## Results

**Patient characteristics:** In Phase I, 65.4% of patients were female with a mean age 59.4 years. In Phase II, 40.0% were female with a mean age of 59.8 years. Figures 1 and 2 describe enrolment of patients and capture and grading of images in Phase I and II of the study respectively.

For peer review only



For peer review only - http://bmjopen.bmj.com/site/about/guidelines.xhtml

177 Figure 1

178

For peer review only

179 Figure 2

180

For peer review only



For peer review only - http://bmjopen.bmj.com/site/about/guidelines.xhtml

**Initial grading performance analysis (Phase I):** The sensitivity for detecting any DR was low against the reference standard in the UK for all grader groups in Vietnam. The sensitivity for detecting referable DR and referable DMO was even lower for all grader groups (Table 1). The sensitivity increased when ungradable images were excluded from the analysis, though it still remained low (47.9-50.8% for any DR; 22.2%-38.1% for referable DR and 9.3-26.5% for referable DMO) (Supplementary, Table S6).

**Table 1. Diagnostic test accuracy of DR graders in Vietnam against a reference standard from the UK, including ungradable images.**

| | Level 1 graders (n=410 patient images)* | Level 2 graders (n=410 patient images)* | Level 3 graders (n=260 patient images )† |
|---|---|---|---|
| **Any DR** | | | |
| Sensitivity (%) (95% CI) | 41.8 (33.9, 50.1) | 42.5 (34.5, 50.7) | 42.2 (33.1, 51.8) |
| Specificity (%) (95% CI) | 87.9 (83.3, 91.7) | 98.8 (96.6, 99.8) | 100 (97.5, 100) |
| PPV (%) (95% CI) | 67.4 (57.0, 76.6) | 95.6 (87.6, 99.1) | 100 (92.7, 100) |
| NPV (%) (95% CI) | 71.7 (66.4, 76.7) | 74.3 (69.3, 78.8) | 68.2 (61.5, 74.5) |
| **Referable DR** | | | |
| Sensitivity (%) (95% CI) | 19.2 (9.63, 32.5) | 13.5 (5.59, 25.8) | 10.5 (2.94, 24.8) |
| Specificity (%) (95% CI) | 97.2 (94.9, 98.7) | 100 (99.0, 100) | 99.5 (97.5, 100) |
| PPV (%) (95% CI) | 50.0 (27.2, 72.8) | 100 (59.0, 100) | 80.0 (28.4, 99.5) |
| NPV (%) (95% CI) | 89.2 (85.7, 92.1) | 88.8 (85.3, 91.7) | 86.7 (81.9, 90.6) |
| **Referable DMO** | | | |
| Sensitivity (%) (95% CI) | 5.8 (1.91, 13.0) | 20.9 (12.9, 31.0) | 16.9 (8.76, 28.3) |
| Specificity (%) (95% CI) | 97.2 (94.8, 98.7) | 99.4 (97.8, 99.9) | 100 (98.1, 100) |
| PPV (%) (95% CI) | 35.7 (12.8, 64.9) | 90.0 (68.3, 98.8) | 100 (71.5, 100) |
| NPV (%) (95% CI) | 79.5 (75.2, 83.4) | 82.6 (78.4, 86.2) | 78.3 (72.7, 83.3) |

**Abbreviations:** UK = United Kingdom, DR = Diabetic Retinopathy, DMO = Diabetic Macular Oedema, CI = Confidence Intervals,
Grading criteria: UK National Diabetic Eye Screening Programme (NDESP) classification system (See supplementary material, Table S1 for more details).
Any DR, is defined as grades R1, R2, R3s, R3a and U.
Referable DR is defined as grades R2, R3a and U
Referable DMO is defined as grades M1 and U
*Missing (n=2, 0.5%), †missing (n=2, 0.8%)

For peer review only



For peer review only - http://bmjopen.bmj.com/site/about/guidelines.xhtml

Sensitivity is the ability of a test to correctly identify patients with a disease and specificity is the ability of a test to correctly identify people without the disease
Positive predictive value (PPV) is the proportion of those who test positive who have the condition (true positives) and negative predictive value (NPV) is the proportion of those who test negative who do not have the condition (true negatives).

**Subsequent grading performance after retraining (Phase II):** Subsequently, a further subset of images from 115 consecutive patients from January to October 2020 were graded by six of the original cohort of 14 Vietnamese graders, and were regraded in the UK to evaluate graders' performance after targeted training was delivered and quality control measures were instituted. The greatest improvement in sensitivity for detecting any DR was seen among level 1 graders (difference: +45.4%, 95%CI +33.1% to +57.8%; P<0.001). The specificity increased from 87.9% in phase I to 95.6% in phase II which helps to avoid over referrals (difference: +7.7%, 95%CI +1.4% to +13.9%; p=0.069). The lowest improvement in sensitivity for detecting any DR was observed between level 3 graders in Vietnam (difference; +9.0%, 95%CI: -9.0% to +27.1%; p=0.326), although their specificity remained 100% at phase I and phase II. There was a significant improvement in sensitivity for detecting DR and referable DMO at all grader levels: sensitivities after training ranged between 40% and 61.5% (Table 2). Further improvement in sensitivity was observed when ungradable images were excluded from the analysis in Phase II: sensitivities ranged from 55.6 to 97.6% for any DR, 55.6%-88.9% for referable DR and 60.0-90.0% for referable DMO (Supplementary, Table S7). The overall prevalence of DR in this study can be found in Supplementary, Table S8.



For peer review only - http://bmjopen.bmj.com/site/about/guidelines.xhtml

**Table 2: Diagnostic test accuracy of DR graders in Vietnam against a reference standard from the UK after additional DR training was delivered.**

| | Level 1 graders (n=115 patient images) | Level 2 graders (n=115 patient images) | Level 3 graders (n=62 patient images) |
|---|---|---|---|
| **Any DR** | | | |
| Sensitivity (%) (95% CI) | 87.2 (74.3, 95.2) | 68.1 (52.9, 80.9) | 51.3 (34.8, 67.6) |
| Specificity (%) (95% CI) | 95.6 (87.6, 99.1) | 95.6 (87.6, 99.1) | 100 (84.6, 100) |
| PPV (%) (95% CI) | 93.2 (81.3, 98.6) | 91.4 (76.9, 98.2) | 100 (83.2, 100) |
| NPV (%) (95% CI) | 91.5 (82.5, 96.8) | 81.3 (71.0, 89.1) | 53.7 (37.4, 69.3) |
| P-value comparing sensitivity to Phase I | P=0.000 | P=0.002 | P=0.326 |
| **Referable DR** | | | |
| Sensitivity (%) (95% CI) | 53.3 (26.6, 78.7) | 40.0 (16.3, 67.7) | 58.3 (27.7, 84.8) |
| Specificity (%) (95% CI) | 90.0 (82.4, 95.1) | 93.0 (86.1, 97.1) | 100 (92.7, 100) |
| PPV (%) (95% CI) | 44.4 (21.5, 69.2) | 46.2 (19.2, 74.9) | 100 (59.0, 100) |
| NPV (%) (95% CI) | 92.8 (85.7, 97.0) | 91.2 (83, 95.9) | 90.7 (79.7, 96.9) |
| P-value comparing sensitivity to Phase I | P=0.009 | P=0.022 | P=0.001 |
| **Referable DMO** | | | |
| Sensitivity (%) (95% CI) | 56.3 (29.9, 80.2) | 43.8 (19.8, 70.1) | 61.5 (31.6, 86.1) |
| Specificity (%) (95% CI) | 97.0 (91.4, 99.4) | 93.9 (87.3, 97.7) | 100 (92.6, 100) |
| PPV (%) (95% CI) | 75.0 (42.8 94.5) | 53.8 (25.1, 80.8) | 100 (63.1, 100) |
| NPV (%) (95% CI) | 93.2 (86.5, 97.2) | 91.2 (83.9, 95.9) | 90.6 (79.3, 96.9) |
| P-value comparing sensitivity to Phase I | P=0.000 | P=0.051 | P=0.002 |

**Abbreviations:** UK = United Kingdom, DR = Diabetic Retinopathy, DMO = Diabetic Macular Oedema, CI = Confidence Intervals,
Grading criteria: UK National Diabetic Eye Screening Programme (NDESP) classification system (See supplementary material, Table S1 for more details).
Criteria:
Any DR is defined as grades R1, R2, R3s, R3a and U.
Referable DR is defined as grades R2, R3a and U
Referable DMO is defined as grades M1 and U
Chi-squared used to compare sensitivity between Phase I and II.
Sensitivity is the ability of a test to correctly identify patients with a disease and specificity is the ability of a test to correctly identify people without the disease
Positive predictive value (PPV) is the proportion of those who test positive who have the condition (true positives) and negative predictive value (NPV) is the proportion of those who test negative who do not have the condition (true negatives).

## Discussion

For peer review only



For peer review only - http://bmjopen.bmj.com/site/about/guidelines.xhtml

Results from our study demonstrated poor sensitivity and specificity for detecting all levels of DR, especially referable DR, in the early stages of programme delivery. This translates into increased costs to the health care system due to missed opportunities for early treatment and un-necessary examinations for false-positive referrals. The quality of patient care also suffers. Didactic DR training was delivered to graders in Vietnam over a two-year period by trained DR graders from the UK and Vietnam. Training was specifically targeted to address problems identified in the Phase I testing [10], and quality control testing using international test and training (iTAT) were also undertaken. The iTAT is an online platform offering monthly quality assurance and training for graders who work in DR screening. It is a useful platform for graders to improve their skills in the detection of DR from ophthalmic images. In the UK, it is compulsory for graders to complete monthly test sets (each set consisting of 20 retinal images with a range of DR severities). If graders fall below the agreed threshold, additional training and support is provided.[7] This study demonstrates that these steps led to improved grading accuracy for all classes of patients and graders. We found that the main discordance between graders lay in their ability to detect ungradable images; therefore, targeted training must be given to ensure such patients are referred to the next level (slit-lamp examination).

According to the UK National Institute for Clinical Excellence (NICE) guidelines, DR screening tests must have at least 80% sensitivity and 95% specificity with a technical failure of 5% or less.[11]These requirements were not met here for sensitivity, but may not be applicable to LMICs. Results can be poor in these settings for a variety of reasons, including higher prevalence of un-operated lens opacity impacting clarity of photographs, use of nurses rather than professional photographers for image capture and poor compliance with photography among patients who have not previously undergone such examinations.



For peer review only

Quality assessment in such settings is crucial, and programmatic changes based on models such as the UK DESP can be successful in enhancing grader accuracy in LMICs settings. However, it is important for countries to adapt their own DR classification system and referral pathways to meet their requirements. As an example, the UK system (England, Wales and Northern Ireland) uses the grade M0 for no maculopathy and M1 for referable maculopathy. In Scotland, M0 denotes no maculopathy, M1 observable maculopathy and M2 referable maculopathy allowing some monitoring of maculopathy to take place on screening level. This reduces the burden on the hospital system. The implication for LMICs is that being aware of hospital capacity at the planning stage might mean that they need to safely adapt an accepted grading system to their needs. Most importantly, the role of affiliated hospitals (and partnerships, coordination among training institutions and practical hospitals) are crucial for DR grading quality improvement.

Studies in LMICs have assessed the accuracy of non-medical graders and medical graders in the detection of DR and found that both grader types are capable of achieving moderate-high sensitivity for detecting DR.[12-15] Comparable with our findings, a study in China found that non-medical DR graders achieved higher sensitivity (0.82-0.94%) and specificity (0.91-0.98%) compared to rural ophthalmologists (sensitivity=0.65-0.95%, specificity=0.59-0.95%) [16]. In DR screening, it is vital to detect referable and STDR to prevent blindness, but it is equally important to detect normal cases to prevent unnecessary referrals to already overburdened hospital clinics. Screening provides an opportunity for graders to discuss with patients the importance of managing diabetes to reduce the risk of visual impairment from DR.

Some studies have described what training interventions were used to train their graders and key elements may be incorporated into our training programme in the future.[15, 17-18] In



For peer review only - http://bmjopen.bmj.com/site/about/guidelines.xhtml

the UK, the DR grading course by the Gloucestershire Retinal Education Group is required for grader certication. The high costs of this course may be more challenging in LMICs due to limited funding.[6]

**Strengths:** This study describes the impact of a training intervention to improve the quality of DR grading in an LMIC. The inclusion of ungradable images in this study was a logical decision, particularly when the prevalence of cataract (which often renders DR images ungradable) is high in LMICs.[19] Dense cataracts normally obstruct the view of the fundus, making it difficult to obtain clear fundus photographs and assign a DR grade. In these instances, referring patients to an eye clinic for further assessment and treatment as needed is required. Determining sensitivity and specificity at the patient level is also important from a DESP implementation perspective. In the UK and Vietnam, both eyes are typically examined for DR and a single outcome is assigned to the patient, as was done here. For these reasons, we feel our analytic approach, and thus results, are relevant to these settings.

**Limitations:** Limitations for this study have also been acknowledged. Data from this study represent routine clinical practice. In daily DR screening, not all patients undergoing primary (Level 1) and secondary (Level 2) grading proceed to arbitration grading (Level 3). This means a proportion of images were not graded by arbitration graders as outlined in figure 1 and figure 2. Second, only 6/14 graders from Phase I were included in Phase II grading; however the distribution of grader levels was similar. Third, though the proportion of patients excluded was small, we are unable to fully characterise the reasons for these exclusions, due to the nature of the study as a programmatic evaluation. Some potential reasons for this are a patient's unwillingness to participate in the study, graders having forgotten to ask for patient consent to participate in the study, and patient inability to comply with image capture. Fourth, pupil status (size and cataract status) was not recorded in this study and this can be important

For peer review only



For peer review only - http://bmjopen.bmj.com/site/about/guidelines.xhtml

for LMICs. Finally, it was not practical for the UK reference standard to examine patients clinically in Vietnam; however, the method of grading by a certified DR grader or clinical specialist is widely used as the reference standard in many screening programmes.

**Conclusions:** This paper shows how grading accuracy was particularly low among all grader groups in Vietnam in the first six months of DESP implementation. Many factors may have contributed to poor grader performance, including inadequate training and feedback, insufficient time to participate in quality assurance testing and competing work responsibilities. After additional training, testing and quality assurance systems were implemented in Vietnam, DTA improved among all grader groups, however more work is still needed. In particular, training graders to detect ungradable cases is crucial. With continuous quality improvement, monthly international test and training, periodic DR workshops and reviewal of certification, we would expect the DR sensitivity and specificity to improve further. A qualitative study to determine why the initial training intervention was less successful should be explored. Since further improvements are required, understanding how other countries implement such programmes would be beneficial. Future studies should outline what DR training interventions were used, state relevant training courses and explain what quality assurance measures are in place. The findings from this study are important for DESP programme planners in Vietnam and other LMICs, highlighting the importance of quality monitoring and directed re-training as needed.

Artificial intelligence (AI) is likely to significantly change future approaches to DR grading. Continued attention to maximising accuracy of human graders is still highly relevant today, especially for low-resource settings, as AI systems must be validated locally against a gold standard of proven expert human graders. Differences between the high-quality images used to train most existing AI systems and the types of images encountered in low-resource



For peer review only - http://bmjopen.bmj.com/site/about/guidelines.xhtml

settings, with high rates of prevalent lens opacity, less-well-trained photographers, and lower-cost cameras, mean that such validation must almost certainly occur at the local level. The continued importance of reliable human graders in low-resource settings is further underscored by the fact that few systems are able to function fully autonomously without input from existing graders.

**Acknowledgements:** The authors are very grateful to all participants in Vietnam and Northern Ireland who generously gave their time for this study and to Orbis International.

**Author contributions:** All authors (KC, NC, TTH, LL, VTN, HTN, QHN, CD, GV, PP, HT, RS, MQT, TP) have made substantial contributions to the conception of the design of the work. KC and GV analysed and interpreted the data. All authors (KC, NC, TTH, LL, VTN, HTN, QHN, CD, GV, PP, HT, RS, MQT, TP) read and approved the final manuscript.

**Funding:** The project was funded by the Department for the Economy (DfE) – Global Challenges Research Fund (GCRF) Awards (Grant number: DFEGCRF17-18/Peto). None of the funders were involved in the design or conduct of the study; preparation, review or approval of the manuscript; or decision to submit the manuscript for publication. Nathan Congdon is supported by the Ulverscroft Foundation (UK) (Grant number: N/A). Katie Curran is funded by the Wellcome Trust (Grant number: 204835/Z/16/A).

**Competing Interests:** Nathan Congdon is employed as a Research Director by Orbis International.

**Patient consent for publication:** Not required.

**Ethics approval:** This study involves human participants and was approved by an Ethics Committee or Institutional Board. This research adhered to the tenets of the Declaration of

For peer review only



For peer review only - http://bmjopen.bmj.com/site/about/guidelines.xhtml

Helsinki. Ethical approval was granted by the Hanoi Medical University Institutional Review Board in Bio-Medical Research, Vietnam (No. 0518/HMU IRB). Written informed consent was obtained from all participants prior to their being interviewed.

**Data sharing statement:** All data relevant to the study are included in the article or uploaded as supplementary information.

**References**


1. Ngoc NB, Lin ZL, Ahmed W. Diabetes: What challenges lie ahead for Vietnam? *Ann Glob Heal*. 2020;86(1):1–9.

2. Liew G, Michaelides M, Bunce C. A comparison of the causes of blindness certifications in England and Wales in working age adults (16-64 years), 1999-2000 with 2009-2010. *BMJ Open.* 2014;4(2):1–6.

3. Burgess PI, Msukwa G, Beare NAV. Diabetic retinopathy in sub-Saharan Africa: Meeting the challenges of an emerging epidemic. *BMC Med*. 2013;11(1):1.

4. Adriono G, Wang D, Octavianus C, Congdon N. Use of eye care services among diabetic patients in urban Indonesia. *Arch Ophthalmol* (Chicago, Ill 1960). 2011;129(7):930.

5. International Council of Ophthalmology. Number of Ophthalmologists in Practice and Training Worldwide [Internet]. 2012 [cited 2020 Aug 13]. Available from: http://www.icoph.org/ophthalmologists-worldwide.html

6. Gloucestershire Retinal Education Group. Diabetic Retinopathy Grading Course [Internet]. 2020. [cited 2021 April] Available from: https://drscreening.org/diabetic-retinopathy-grading-course




For peer review only - http://bmjopen.bmj.com/site/about/guidelines.xhtml

7. Widdowson S, Taylor D. The management of grading quality: Good practice in the quality assurance of grading. [Internet] 2015. [cited 2021 April] Available from: https://assets.publishing.service.gov.uk/government/uploads/system/uploads/attachment_data/file/512832/The_Management_of_Grading.pdf

8. Public Health England. NHS Diabetic Eye Screening Programme Grading definitions for referable disease. [Internet] 2021 (cited 2021 September] Available from: https://www.gov.uk/government/publications/diabetic-eye-screening-retinal-image-grading-criteria/nhs-diabetic-eye-screening-programme-grading-definitions-for-referable-disease

9. NHS DESP Core Team. Diabetic Eye Screening Programme Pathway for Adequate / Inadequate Images and where images cannot be taken Outlines pathway and business rules for image capture exceptions and ungradable images. [Internet] 2013.[cited 2021 April] Available from: https://www.gov.uk/government/publications/diabetic-eye-screening-pathway-for-images-and-where-images-cannot-be-taken

10. Curran K, Congdon N, Peto T, Dardis C, Nguyen NN, Hoange TT et al. Capturing the clinical decision-making processes of expert and novice diabetic retinal graders using a 'think-aloud' approach. *Eye*. 2022;36:1019-1026

11. Scanlon PH. The English national screening programme for diabetic retinopathy 2003–2016. *Acta Diabetol*. 2017;54(6):515–525.

12. Amirul Islam FM. Accuracy and reliability of retinal photo grading for diabetic retinopathy: Remote graders from a developing country and standard retinal photo grader in Australia. *PLoS One*. 2017;12(6):1–12.

13. Bhargava M, Cheung CYL, Sabanayagam C, Kawasaki R, Harper CA, Lamoureux EL, et al. Accuracy of diabetic retinopathy screening by trained non-physician graders using non-mydriatic fundus camera. *Singapore Med J.* 2012;53(11):715–9.



For peer review only - http://bmjopen.bmj.com/site/about/guidelines.xhtml

14, Piyasena MMPN, Yip JLY, MacLeod D, Kim M, Gudlavalleti VSM. Diagnostic test accuracy of diabetic retinopathy screening by physician graders using a hand-held non-mydriatic retinal camera at a tertiary level medical clinic. *BMC Ophthalmol.* 2019;19(1):89.

15. Thapa R, Bajimaya S, Pradhan E, Sharma S, Kshetri B, Paudyal G. Agreement on Grading Retinal Findings of Patients with Diabetes Using Fundus Photographs by Allied Medical Personnel when Compared to an Ophthalmologist at a Diabetic Retinopathy Screening Program in Nepal. *Clin Ophthalmol*. 2020;14:2731-2737.

16. McKenna M, Chen T, McAneney H, Membrillo MAV, Jin L, Xiao W, et al. Accuracy of trained rural ophthalmologists versus non-medical image graders in the diagnosis of diabetic retinopathy in rural China. *Br J Ophthalmol*. 2018;102(11):1471–6.

17. Rani PK, Peguda HK, Chandrashekher M, Swarna S, Jonnadula GB, James J et al. Capacity building for diabetic retinopathy screening by optometrists in India: Model description and pilot results. *Indian J Ophthalmol*. 2021.69:655-659.

18. Srinivasan S, Shetty S, Natarajan V, Sharma T, Raman R. Development and Validation of a Diabetic Retinopathy Referral Algorithm Based on Single-Field Fundus Photography. *PLoS One* 2016.11:e0163108.

19. Murthy GVS, John N, Shamanna BR, Pant HB. Elimination of avoidable blindness due to cataract: Where do we prioritize and how should we monitor this decade. *Indian J Ophthalmol.* 2012;60(5):438–45. (19)

Figure 1: Flow diagram to illustrate enrolment of patients and management of images in Phase I from August to October 2018 (Initial grading performance analysis). Level 1 and level 2 graders graded the same set of photographs and level 3 graders graded a subset of these photographs: All disagreements between Level 1 and 2 graders and a 40% random sample of all images.

For peer review only



For peer review only - http://bmjopen.bmj.com/site/about/guidelines.xhtml

Figure 2: Flow diagram illustrating the enrolment of patients and management of images included in Phase II from January 2020 to October 2020 (Follow-up grading performance analysis after re-training). Level 1 and level 2 graders graded the same set of photographs and level 3 graders graded a subset of these images.

For peer review only

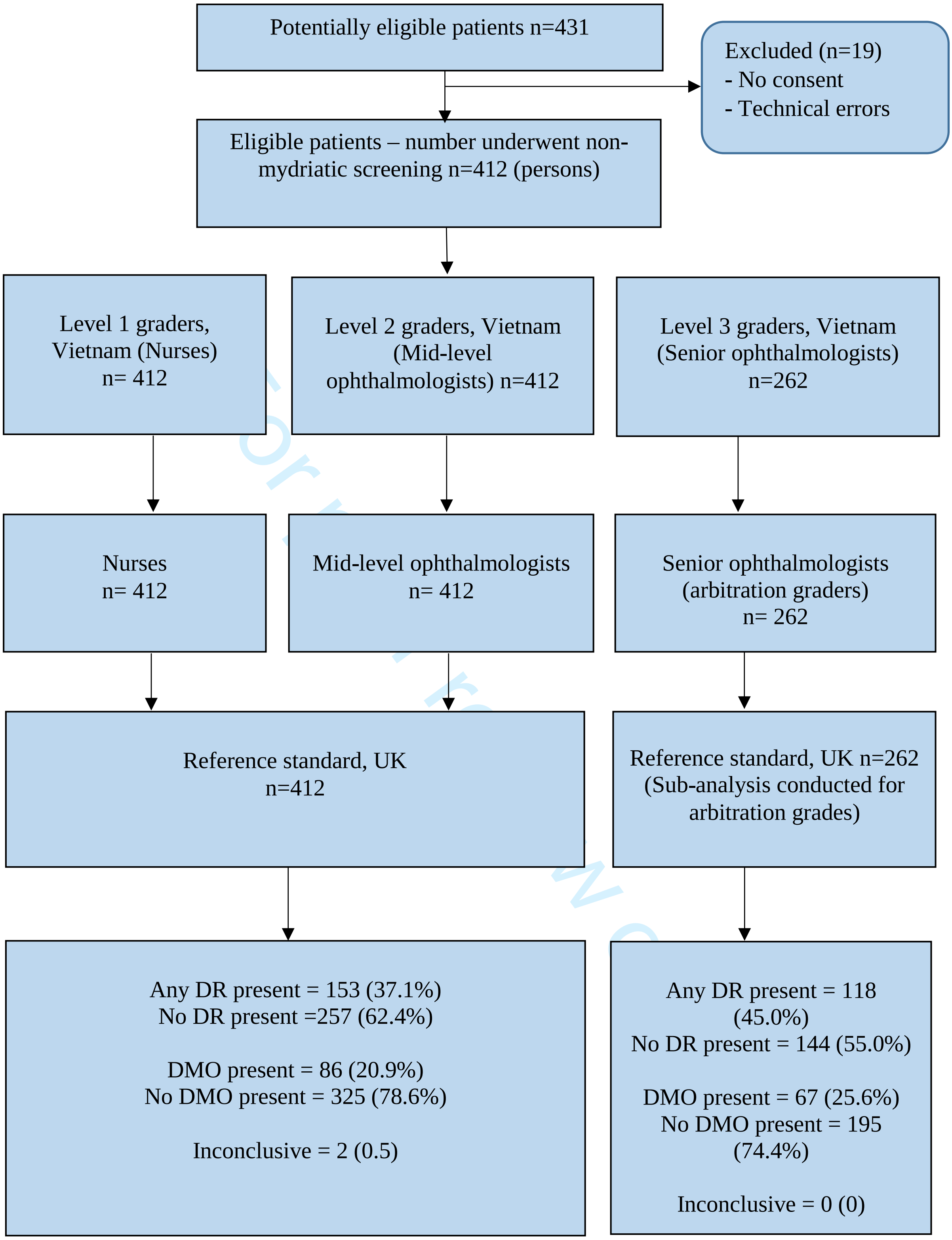
Potentially eligible patients n=431
Excluded (n=19)
- No consent
- Technical errors
Eligible patients – number underwent non-mydriatic screening n=412 (persons)
Level 1 graders, Vietnam (Nurses) n= 412
Level 2 graders, Vietnam (Mid-level ophthalmologists) n=412
Level 3 graders, Vietnam (Senior ophthalmologists) n=262
Nurses n= 412
Mid-level ophthalmologists n= 412
Senior ophthalmologists (arbitration graders) n= 262
Reference standard, UK n=412
Reference standard, UK n=262 (Sub-analysis conducted for arbitration grades)
Any DR present = 153 (37.1%)
No DR present =257 (62.4%)
DMO present = 86 (20.9%)
No DMO present = 325 (78.6%)
Inconclusive = 2 (0.5)
Any DR present = 118 (45.0%)
No DR present = 144 (55.0%)
DMO present = 67 (25.6%)
No DMO present = 195 (74.4%)
Inconclusive = 0 (0)

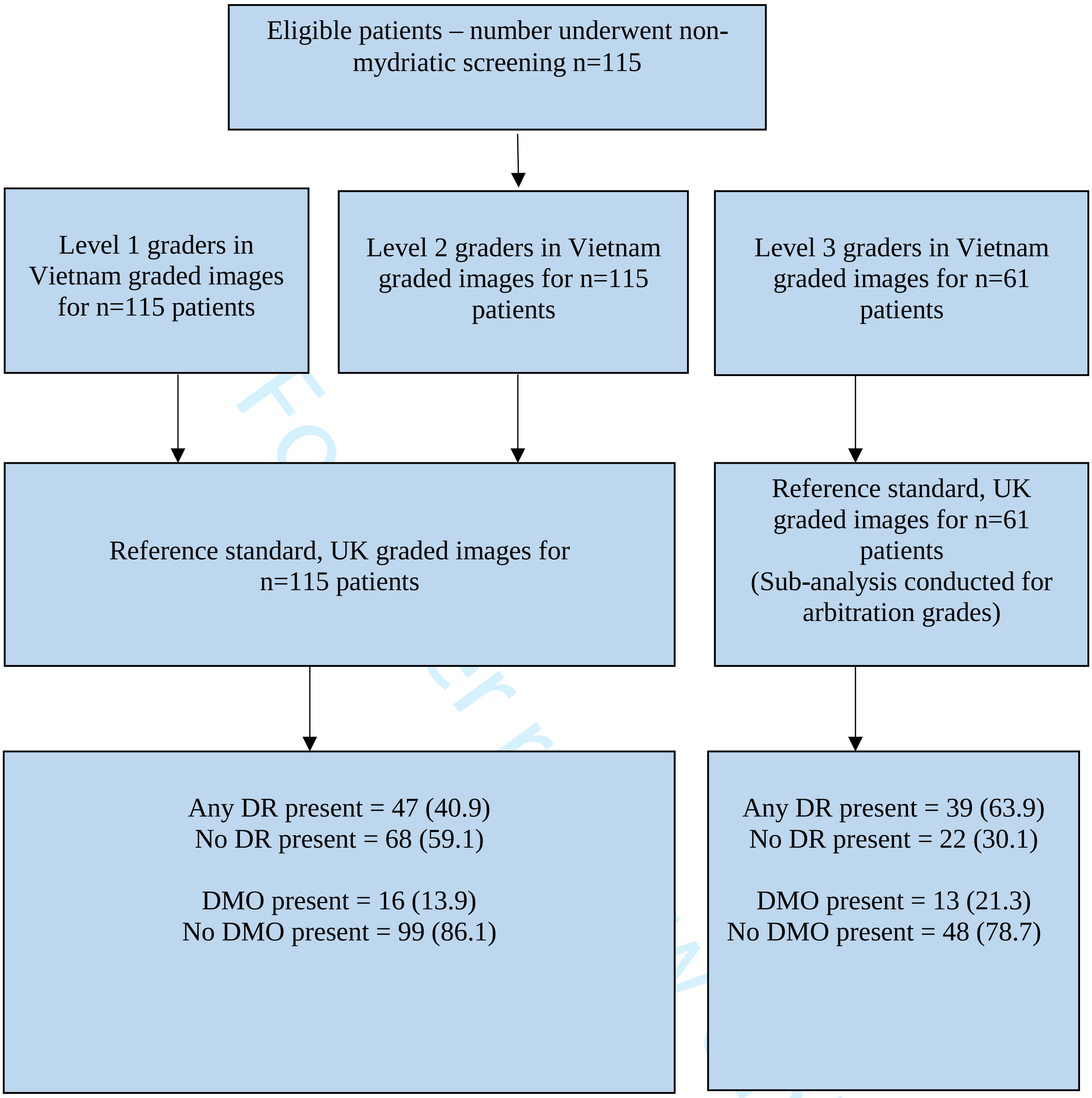
Eligible patients – number underwent non-mydriatic screening n=115
Level 1 graders in Vietnam graded images for n=115 patients
Level 2 graders in Vietnam graded images for n=115 patients
Level 3 graders in Vietnam graded images for n=61 patients
Reference standard, UK graded images for n=115 patients
Reference standard, UK graded images for n=61 patients (Sub-analysis conducted for arbitration grades)
Any DR present = 47 (40.9)
No DR present = 68 (59.1)
DMO present = 16 (13.9)
No DMO present = 99 (86.1)
Any DR present = 39 (63.9)
No DR present = 22 (30.1)
DMO present = 13 (21.3)
No DMO present = 48 (78.7)

**SUPPLEMENTARY MATERIAL**

**Figure S1: Training timeframe for graders in Vietnam**

**September 2017 (5 days)**

- Five Vietnamese doctors and medical administrators visited Northern Ireland to receive screener/graders training in administration and failsafe methods.
- These doctors delivered training to new graders in Vietnam following their visit to Northern Ireland.

**January 2018 (1 week) – Delivered in person in Vietnam by two UK graders (senior ophthalmic nurse and optometrist, certified in DR grading)**

- Observation in retina clinics.
- Hands on training with tabletop CR-2 Canon Fundus Cameras.
- Topics covered: ocular anatomy, retinal disease, DR signs, DR grading (based on the UK DESP grading classification system) and appropriate referral pathways and management (PowerPoint presentation and interactive sessions).
- All graders received a module workbook.
- Certifcation provided by Orbis.

**March 2018 - Grading began in Vietnam**

- Graders began grading as part of pilot DESP.
- Ongoing training was delivered by the Orbis team and the lead ophthalmologist for DR screening in Vietnam over the course of the following months.

**June 2018 (1-2 days) – Delivered by Orbis partners**

- UK graders developed a PowerPoint presentation based on DR case examples and this was delivered by Orbis.

**March 2019 (2 days) - Delivered by UK grader in Vietnam**

- More training on DR case examples.

**November 2019 (3 days) - Delivered in person in Vietnam by two UK graders (senior ophthalmic nurse and optometrist, certified in DR grading)**

- Refresher DR training, incorporating think-aloud techniques into practical teaching sessions.
- Pre and post training assessments.
- Encouraged use of international test and training (iTAT) for quality assurance purposes. Practical sessions on iTAT.

For peer review only

**Table S1: Diabetic retinopathy workshop for graders in Vietnam**

| | | |
|---|---|---|
| **AGENDA**<br><br>DAY 1-3 **Monday 29th January – Wednesday 31st January** | | |
| Visit diabetic retinopathy screening sites to observe and provide hands on training and support. | | UK graders and Orbis team |
| DAY 4- **Thursday 1st February** | | |
| **MORNING** | | |
| **9:00 - 9:30** | Check in | UK graders and Orbis team |
| **9:30 – 10.30** | Introduction on Diabetic Retinopathy | |
| **10.30-10.45** | Tea Break | |
| **10.45-11.30** | Basic Screening Component | |
| | Lunch | |
| **AFTERNOON** | | |
| **13.30-14:00** | NHS Grading System | UK graders and Orbis team |
| **14:00-14:45** | Image Quality | |
| **14:45-15:00** | Tea Break | |
| **15:00-16:00** | Image Grading | |
| **16:0-16:30** | Hospital pathway | |
| DAY 5- **Friday 2nd February** | | |
| **MORNING** | | |
| **9:00-9:30** | Other ocular findings | UK graders and Orbis team |
| **9:30-11:30** | Practice on grading | |
| **11:30-12:00** | Wrap up | |

For peer review only

**Table S2: Refresher training programme for DR graders in Vietnam (delivered by UK graders and Orbis) in November 2019**

| Time | Topics | Method | Who | Preparation |
|---|---|---|---|---|
| **DAY 1** | | | | |
| **8:45-9:15** | Introduction/ Pre course quiz | | Orbis VN/ UK graders | UK graders |
| **9:15-9:45** | **Diabetic Retinopathy (DR) - New Challenges of Blindness Prevention** *Objective: Understand the problem of DR and current efforts to manage vision loss. Aim to motivate graders to be involved in DESP* | Presentation | Orbis VN | Orbis |
| **9:45-10:15** | **Retina Anatomy** *Objective : Understand the pathobiology of diabetic complications and pathogenesis of retinal damage* | Presentation | Ho Chi Minh Eye Hospital | Orbis |
| **10:30-11:15** | **Diabetic Retinopathy (DR) Pathophysiology** *Objective : Understand Diabetic Retinopathy* | Presentation | UK graders | UK graders |
| **11:15-12:00** | **Grading system and DR Grading pathway ( UK system)/ How to systematically grade a retinal image** *Objective : Understanding the grading system and referral pathway ( UK standard)* | Presentation | UK graders | UK graders |
| **13:30-14:15** | **Image quality** *Objective : Understand the requirements/criteria of image quality for accurate grading* | Presentation | UK graders | UK graders |
| **14:15-14:45** | **Spectra Software** *Objective : How to use the current Spectra software for uploading, grading, and managing DR cases* | Demonstration | Senior graders of Tien Giang and Ho Chi Minh Eye Hospital | |

For peer review only

| | | | | |
|---|---|---|---|---|
| **15:00-17:00** | **<u>Practical Training</u>**<br>**Parallel session:**<br>**1- Taking retina images of the patients following the procedure ,and provide counseling to the patients**<br><br>*Objective: Practice experience of taking fundus images* | Practical training | Current graders of Tien Giang and Ho Chi Minh Eye Hospital, and UK graders | 2 fundus cameras:<br>4 Groups: make sure every participant is able to practice at least once |
| **15:00-17:00** | **Parallel session :**<br>**1- Grading DR in the Spectra**<br><br>*Objective: Practical experience of how to do DR grading* | Practical training | Current graders of Tien Giang and Ho Chi Minh Eye Hospital, and UK graders | 3-4 accounts of Spectra<br>4 Groups :<br>make sure every participant is able to practice at least once |
| | | **DAY 2** | | |
| **08:45-09:00** | *Recap of day 1/ introduction to day 2* | | Orbis | |
| **9:00-9:30** | **Analysis of retinal fundus images for grading of diabetic retinopathy severity**<br>Objective : How to read the image and protocol for retinal image analysis | | Ophthalmologist Ho Chi Minh Eye Hospital | |
| **9:30-10:15** | **DR Screening Procedure: Best Practice**<br>*Objective: Discuss how to build the "best screening procedures" into DESPs.* | Presentation | UK graders | UK graders |
| **10:30-11:15** | **Other Ocular Findings**<br><br>*Objective: Awareness of other ocular pathology during DR screening* | Presentation | UK graders | UK graders |
| **11:15-12:15** | **Image grading case studies competition** | Practical | UK graders | We need to organise people into groups of 3 with one experienced grader in each group |

| | | | | |
|---|---|---|---|---|
| **13:30-14:30** | **Counselling and delivering messages to patients during the DR screening**<br>*Objective: Important to provide counselling for the patients and deliver messages effectively.* | Presentation/ practical training | Orbis Vietnam | |
| **14:30-16:30** | **<u>Practical Training</u>**<br>**Parallel session:**<br>**1- Taking retina images of the patients following the procedure ,and provide counseling to the patients**<br><br>*Objective: Experience on how to take good fundus photographs* | Practical training | Current graders of Tien Giang and Ho Chi Minh Eye Hospital, and UK graders | 2 fundus camera<br>4 Group: make sure every participants are able to practice at least one time |
| | **Parallel session :**<br>**1- Grading DR in the Spectra**<br><br>*Objective: Practical experience on DR grading* | Practical training | Current graders of Tien Giang and Ho Chi Minh Eye Hospital, and UK graders | -4 accounts of Spectra<br>4 Groups: make sure every participant is able to practice at least one time. |
| **DAY 3**<br>**Part 1 (Final practical training)** | | | | |
| **08:45-9:00** | Recap of day 2/ introduction to day 3 | | | |
| **09:00-09:30** | **Quality Assurance in Diabetic Screening**<br>*Objective : Understand why quality assurance is important and the correct steps required to ensure good quality assurance procedures are in place* | Presentation | UK graders | UK graders |
| **09:30-10:30** | **Practice : Grading in iTAT**<br>*Objective: Know the Online training for DR grading and the importance of lifelong learning for DR grading* | Practice | Current graders of Tien Giang and Ho Chi Minh Eye Hospital, and support from UK graders | ITAT accounts for practicing |

| | | | | |
|---|---|---|---|---|
| **10:45-12:30** | **<u>Assessment</u>**<br><br>**Parallel session:**<br>**1- Taking retina images of the patients following the procedure ,and provide counseling to the patients**<br>**2- Grading DR in the Spectra** | Practical Training | Current graders of Tien Giang and Ho Chi Minh Eye Hospital, and UK graders | 2 fundus cameras<br>4 Group and 4 accounts of Spectra<br>4 Groups |
| **DAY 3**<br>**Part 2 (Future planning)** | | | | |
| **13:30-14:15** | **Post course Quiz and results** | | UK graders | UK graders |
| **14:15-15:00** | **Teaching Methodology for adults**<br>*Objective : How to train new graders effectively* | Think aloud work shop | Orbis VN Supported by UK graders | UK graders |
| **15:15-16:00** | **Supportive Supervision Methodology: Developing quality improvement**<br>*Objective: How to plan, implement the supervision trips to correct / improve other graders' performances. Provide checklist tools* | Think aloud work shop | Orbis VN Supported by UK graders | UK graders |
| **16:00-16:45** | **Feedbacks and Plan for next steps** | Discussion | | |
| **16:45-17:00** | **Certificates for Vietnamese graders in attendance** | | | |

For peer review only

**Table S3: UK DR Grading Classification Scale**

| NSC | International Term | Symptoms | Features | Action |
|---|---|---|---|---|
| **R0** | No DR | None | No signs of diabetic retinopathy | Annual rescreen |
| **RI** | Mild none-proliferative (mild pre-proliferative) | None | Haemorrhages & microaneurysms, only | Annual rescreen |
| **R2** | Moderate none-proliferative, moderate pre-proliferative | None | Extensive Microaneurysms, intraretinal haemorrhages, hard exudates, venous abnormalities, large blot haemorrhages, cotton wool spots (small infarcts), venous beading, venous loop, venous reduplication. | Refer routinely to HES |
| **R3s** | Stable proliferative diabetic retinopathy | | No haemorrhages or exudates or new vessels, laser scars | Annual rescreen |
| **R3a** | Active proliferative diabetic retinopathy | Floaters, central loss of vision | New vessel formation either at the disc (NVD) or elsewhere (NVE). Extensive fibrovascular proliferation, retinal detachment, pre-retinal or vitreous haemorrhage. | Urgent referral to HES |
| **M 0** | | | No maculopathy | Annual rescreen |
| **M 1** | Diabetic maculopathy | Blurred central vision | The macula is defined as a circle centred on the fovea, with a radius of the distance to the disc margin.<br>If the leakage involves or is near the fovea the condition is termed clinically significant macular oedema (CSME).<br><br>Exudative maculopathy presents with leakage, retinal thickening, microaneurysms, hard exudates at the macula. Ischaemic form can have a featureless macular with NVE and poor vision.<br>Milder forms:<br>• exudate < or = 1DD of centre of fovea<br>• circinate or group of exudates within macula<br>• any microaneurysm or haemorrhage < or = 1DD of centre of fovea only is associated with a best VA of < or = 6/12 retinal thickening < or = | Refer to HES |

| | | | | |
|---|---|---|---|---|
| | | | 1DD of centre of fovea (if stereos available) | |
| **P** | Photocoagulation | Reduced night vision, glare | Small retinal scars throughout the peripheral retina. | |
| **U** | Ungradable | | Ungradable is usually due to cataract, small pupils, other lesions usually referred for assessment | Refer for slit lamp examination |

**Abbreviations:** DR = diabetic retinopathy, NPDR = none-proliferative retinopathy, NVE = new vessels elsewhere, IRMAs = intraretinal microvascular abnormalities (part of severe pre-proliferative retinopathy, vessels will not leak with angiogram, otherwise they would be 'new vessels' making the condition 'proliferative'), MO=macular oedema, MA= microaneurysm, DD=disc diameter, HES= hospital eye service

For peer review only

**Table S4: Reference standards intra-rater agreement score using kappa statistic (first attempt versus second attempt)**

| | Intra-rater agreement (reference standard, UK), k (95% CI) (by eyes, n=106) | Intra-rater agreement (reference standard, UK), k (95% CI) (by worst eye, n=53) |
|---|---|---|
| **Overall Diabetic Retinopathy Grading:** | | |
| **Any DR** | 0.96 (0.91,1.00) | 0.92 (0.82, 1.00) |
| **Treatable DR** | 0.81 (0.60, 1.00) | 0.74 (0.47, 1.00) |
| **Referable Maculopathy** | 0.97 (0.92, 1.0)0 | 1.00 (1.00, 1.00) |
| Abbreviations: CI=confidence interval, k=kappa, DR=Diabetic retinopathy, DMO=diabetic macular oedema<br>Any DR defined as R1, R2, R3s, R3a and U<br>Treatable DR defined as R3a<br>Referable DMO defined as M1 and U | | |

**Table S5: Using kappa statistic to determine the inter-rater agreement between the reference standard and one senior grader from QUB grading centre**

| | Inter-rater agreement (reference standard vs a senior grader QUB), k (95% CI) (by eyes, n=106) | Inter-rater agreement (reference standard vs a senior grader QUB (by worst), k (95% CI) (by worst eye, n=53) |
|---|---|---|
| **Overall Diabetic Retinopathy Grading:** | | |
| **Any DR** | 0.79 (0.67, 0.91) | 0.74 (0.55, 0.92) |
| **Treatable DR** | 0.71 (0.48, 0.95) | 0.68 (0.39, 0.97) |
| **Referable Maculopathy** | 0.75 (0.61, 0.90) | 0.74 (0.55, 0.93) |
| Abbreviations: CI=confidence interval, k=kappa, DR=Diabetic retinopathy, DMO=diabetic macular oedema<br>Any DR defined as R1, R2, R3s, R3a and U<br>Treatable DR defined as R3a<br>Referable DMO defined as M1 and U | | |

**Intra and inter-grader agreement**

To ensure there was good intra-grader reliability as a reference standard, a stratified random sample of images were regraded. There was approximately one month between the first and second attempts to reduce the possibility of bias caused by memory. Additionally, inter-grader agreement was calculated using kappa to ensure there was good grading agreement between the reference standard and one senior grader from the Ophthalmic Reading Centre at QUB, Belfast. Any disagreements were discussed with

For peer review only

a retinal specialist until consensus was reached. Overall, the intra-grader agreement and inter-grader agreement ranged from substantial to almost perfect.

For peer review only

**Table S6. Diagnostic test accuracy of DR graders in Vietnam against a reference standard from the UK, excluding ungradable images (Phase I)**

| | **Level 1 graders (n=373 patient images)** | **Level 2 graders (n=373 patient images)*** | **Level 3 graders (n=235 patient images )†** |
|---|---|---|---|
| **Any DR** | | | |
| Sensitivity (%) (95% CI) | 47.9 (38.8, 57.2) | 50.8 (41.6, 60.0) | 49.0 (38.7, 59.3) |
| Specificity (%) (95% CI) | 89.7 (85.1, 93.0) | 98.8 (96.3, 99.7) | 100 (96.8, 100) |
| PPV (%) (95% CI) | 69.0 (57.9, 78.4) | 95.3 (86.2, 98.8) | 100 (90.6, 100) |
| NPV (%) (95% CI) | 78.2 (72.9, 82.7) | 80.9 (76.0, 85.0) | 74.6 (67.8, 80.5) |
| **Referable DR** | | | |
| Sensitivity (%) (95% CI) | 38.1 (19.0, 61.3) | 28.6 (12.2, 52.3) | 22.2 (7.4, 48.1) |
| Specificity (%) (95% CI) | 98.9 (96.9, 99.6) | 100 (98.7, 100) | 99.5 (97.1, 99.9) |
| PPV (%) (95% CI) | 66.7 (35.4, 88.7) | 100 (51.7, 100) | 80.0 (29.9, 98.9) |
| NPV (%) (95% CI) | 96.4 (93.8, 97.9) | 96.0 (93.3, 97.6) | 94.0 (89.9, 96.6) |
| **Referable DMO** | | | |
| Sensitivity (%) (95% CI) | 9.3 (3.0, 23.1) | 37.2 (23.4, 53.3) | 26.5 (13.5, 44.7) |
| Specificity (%) (95% CI) | 99.1 (97.0, 99.8) | 99.4 (97.5, 99.9) | 100 (97.6, 100) |
| PPV (%) (95% CI) | 57.1 (20.2, 88.2) | 88.9 (63.9, 98.1) | 100 (62.9, 100) |
| NPV (%) (95% CI) | 88.9 (85.1, 92.0) | 92.3 (88.9, 94.7) | 88.6 (83.5, 92.4) |

**Abbreviations:** UK = United Kingdom, DR = Diabetic Retinopathy, DMO = Diabetic Macular Oedema, CI = Confidence Intervals,
Grading criteria: UK National Diabetic Eye Screening Programme (NDESP) classification system (See supplementary material, Table S1 for more details).
Any DR, is defined as grades R1, R2, R3s and R3a.
Referable DR is defined as grades R2 and R3a.
Referable DMO is defined as grades M1
Sensitivity is the ability of a test to correctly identify patients with a disease and specificity is the ability of a test to correctly identify people without the disease
Positive predictive value (PPV) is the proportion of those who test positive who have the condition (true positives) and negative predictive value (NPV) is the proportion of those who test negative who do not have the condition (true negatives).

For peer review only

**Table S7: Diagnostic test accuracy of DR graders in Vietnam against a reference standard from the UK after additional DR training was delivered, excluding ungradable images (Phase II)**

| | Level 1 graders (n=115 patient images) | Level 2 graders (n=115 patient images) | Level 3 graders (n=62 patient images) |
|---|---|---|---|
| **Any DR** | | | |
| Sensitivity (%) (95% CI) | 97.6 (85.6, 99.9) | 72.5 (55.9 84.9) | 55.6 (38.1, 72.1) |
| Specificity (%) (95% CI) | 95.6 (86.8, 99.8) | 100 (93.5, 100) | 100 (84.6, 100) |
| PPV (%) (95% CI) | 93.0 (79.9, 98.2) | 100 (85.4, 100) | 100 (80.0, 100) |
| NPV (%) (95% CI) | 98.5 (90.7, 99.9) | 85.5 (75.2, 92.2) | 57.9 (10.9, 73.2) |
| **Referable DR** | | | |
| Sensitivity (%) (95% CI) | 88.9 (50.7, 99.4) | 55.6 (22.7, 84.7) | 77.8 (40.0, 97.2) |
| Specificity (%) (95% CI) | 90.0 (81.9, 94.8) | 96.9 (90.5, 99.2) | 100 (92.8, 100) |
| PPV (%) (95% CI) | 44.4 (22.4, 68.7) | 62.5 (25.9, 89.8) | 100 (56.1, 100) |
| NPV (%) (95% CI) | 98.9 (93.4, 99.9) | 95.9 (89.2, 98.7) | 96.1 (87.8, 98.8) |
| **Referable DMO** | | | |
| Sensitivity (%) (95% CI) | 90.0 (54.1, 99.5) | 60.0 (26.4, 86.3) | 80.0 (44.4, 97.5) |
| Specificity (%) (95% CI) | 97.0 (91.8, 99.2) | 97.9 (91.9, 99.6) | 100 (92.6, 100) |
| PPV (%) (95% CI) | 75.0 (42.8, 93.3) | 75.0 (35.6, 95.5) | 100 (59.8, 100) |
| NPV (%) (95% CI) | 99.0 (93.6, 99.9) | 95.9 (89.2, 98.7) | 96.0 (87.4, 99.6) |

**Abbreviations:** UK = United Kingdom, DR = Diabetic Retinopathy, DMO = Diabetic Macular Oedema, CI = Confidence Intervals,
Grading criteria: UK National Diabetic Eye Screening Programme (NDESP) classification system (See supplementary material, Table S1 for more details).
Criteria:
Any DR is defined as grades R1, R2, R3s, and R3a.
Referable DR is defined as grades R2 and R3a.
Referable DMO is defined as grades M1.
Sensitivity is the ability of a test to correctly identify patients with a disease and specificity is the ability of a test to correctly identify people without the disease
Positive predictive value (PPV) is the proportion of those who test positive who have the condition (true positives) and negative predictive value (NPV) is the proportion of those who test negative who do not have the condition (true negatives).

For peer review only

**Table S8: The prevalence of any diabetic retinopathy (DR), referable DR, any maculopathy and ungradable cases with the reference grader from Phase I and Phase II**

| Diabetic Retinopathy grades | Phase I | Phase II (post remedial training) | P-Value |
|---|---|---|---|
| R0 (n,%) | 257 (62.68) | 68 (59.13) | P=0.347 |
| R1 (n,%) | 100 (24.39) | 32 (27.83) | |
| R2 (n,%) | 11 (2.68) | 2 (1.74) | |
| R3a (n,%) | 10 (2.44) | 7 (6.09) | |
| R3s (n,%) | 1 (0.24) | 0 (0.00) | |
| U (n,%) | 31 (7.56) | 6 (5.22) | |
| **Any DR** | | | |
| - Yes (n,%) | 153 (37.32) | 47 (40.87) | P=0.488 |
| - No (n,%) | 257 (62.68) | 68(59.12) | |
| **Referable DR** | | | |
| - Yes (n,%) | 52 (12.68) | 15 (13.04) | P=0.918 |
| - No (n,%) | 358 (87.32) | 100 (86.96) | |
| **Any DMO** | | | |
| - M0 (n,%) | 324 (79.02) | 99 (86.09) | |
| - M1 (n,%) | 43 (10.49) | 10 (8.70) | P=0.173 |
| - U (n,%) | 43 (10.49) | 6 (5.22) | |
| **Abbreviations:** DR=diabetic retinopathy, DMO=Diabetic Macular Oedema, U=ungradable, Chi-Squares used to test significance. | | | |

For peer review only

| Section & Topic | No | Item | Reported on page # |
|---|---|---|---|
| **TITLE OR ABSTRACT** | | | |
| | **1** | Identification as a study of diagnostic accuracy using at least one measure of accuracy (such as sensitivity, specificity, predictive values, or AUC) | 1 |
| **ABSTRACT** | | | |
| | **2** | Structured summary of study design, methods, results, and conclusions (for specific guidance, see STARD for Abstracts) | 3-4 |
| **INTRODUCTION** | | | |
| | **3** | Scientific and clinical background, including the intended use and clinical role of the index test | 5-6 |
| | **4** | Study objectives and hypotheses | 5 |
| **METHODS** | | | |
| *Study design* | **5** | Whether data collection was planned before the index test and reference standard were performed (prospective study) or after (retrospective study) | 5 |
| *Participants* | **6** | Eligibility criteria | 6-7 |
| | **7** | On what basis potentially eligible participants were identified (such as symptoms, results from previous tests, inclusion in registry) | 6-7 |
| | **8** | Where and when potentially eligible participants were identified (setting, location and dates) | 6-7 |
| | **9** | Whether participants formed a consecutive, random or convenience series | 7 |
| *Test methods* | **10a** | Index test, in sufficient detail to allow replication | 6-7 |
| | **10b** | Reference standard, in sufficient detail to allow replication | 6 |
| | **11** | Rationale for choosing the reference standard (if alternatives exist) | 6 |
| | **12a** | Definition of and rationale for test positivity cut-offs or result categories of the index test, distinguishing pre-specified from exploratory | |
| | **12b** | Definition of and rationale for test positivity cut-offs or result categories of the reference standard, distinguishing pre-specified from exploratory | |
| | **13a** | Whether clinical information and reference standard results were available to the performers/readers of the index test | 6-7 |
| | **13b** | Whether clinical information and index test results were available to the assessors of the reference standard | 6-7 |
| *Analysis* | **14** | Methods for estimating or comparing measures of diagnostic accuracy | 12-14 |
| | **15** | How indeterminate index test or reference standard results were handled | 12-14 Supplementary file |
| | **16** | How missing data on the index test and reference standard were handled | 12-14 |
| | **17** | Any analyses of variability in diagnostic accuracy, distinguishing pre-specified from exploratory | 12-14 |
| | **18** | Intended sample size and how it was determined | NA |
| **RESULTS** | | | |
| *Participants* | **19** | Flow of participants, using a diagram | 10-11 |
| | **20** | Baseline demographic and clinical characteristics of participants | 9 |
| | **21a** | Distribution of severity of disease in those with the target condition | Supplementary file |
| | **21b** | Distribution of alternative diagnoses in those without the target condition | Supplementary file |
| | **22** | Time interval and any clinical interventions between index test and reference standard | Supplementary file |
| *Test results* | **23** | Cross tabulation of the index test results (or their distribution) by the results of the reference standard | 12-14 and supplementary |
| | **24** | Estimates of diagnostic accuracy and their precision (such as 95% confidence intervals) | 12-14 |
| | **25** | Any adverse events from performing the index test or the reference standard | NA |
| **DISCUSSION** | | | |
| | **26** | Study limitations, including sources of potential bias, statistical uncertainty, and generalisability | 17 |
| | **27** | Implications for practice, including the intended use and clinical role of the index test | 17-18 |
| **OTHER INFORMATION** | | | |
| | **28** | Registration number and name of registry | NA |
| | **29** | Where the full study protocol can be accessed | NA |

For peer review only - http://bmjopen.bmj.com/site/about/guidelines.xhtml

| | | | |
|---|---|---|---|
| | **30** | Sources of funding and other support; role of funders | 18 |

For peer review only
